# Macrophage-mediated IL-6 signaling drives ryanodine receptor–2 calcium leak in postoperative atrial fibrillation

**DOI:** 10.1172/JCI187711

**Published:** 2025-03-06

**Authors:** Joshua A. Keefe, Yuriana Aguilar-Sanchez, J. Alberto Navarro-Garcia, Isabelle Ong, Luge Li, Amelie Paasche, Issam Abu-Taha, Marcel A. Tekook, Florian Bruns, Shuai Zhao, Markus Kamler, Ying H. Shen, Mihail G. Chelu, Na Li, Dobromir Dobrev, Xander H.T. Wehrens

**Affiliations:** 1 Cardiovascular Research Institute,; 2 Department of Integrative Physiology, and; 3 Department of Medicine, Baylor College of Medicine, Houston, Texas, USA.; 4 Department of Cardiology, Angiology and Pneumology, University Hospital Heidelberg, Heidelberg, Germany.; 5 Institute of Pharmacology, West German Heart and Vascular Center, University Duisburg-Essen, Essen, Germany.; 6 Department of Thoracic and Cardiovascular Surgery, West German Heart and Vascular Center Essen, University Hospital Essen, Essen, Germany.; 7 Department of Surgery, Division of Cardiothoracic Surgery and; 8 Department of Internal Medicine, Division of Cardiology, Baylor College of Medicine, Houston, Texas, USA.; 9 Texas Heart Institute at Baylor St. Luke’s Medical Center, Houston, Texas, USA.; 10 Department of Medicine, Montreal Heart Institute and Université de Montréal, Montreal, Quebec, Canada.; 11 Department of Neuroscience,; 12 Department of Pediatrics, and; 13 Center for Space Medicine, Baylor College of Medicine, Houston, Texas, USA.

**Keywords:** Cardiology, Immunology, Arrhythmias, Calcium signaling, Macrophages

## Abstract

Postoperative atrial fibrillation (poAF) is AF occurring days after surgery, with a prevalence of 33% among patients undergoing open-heart surgery. The degree of postoperative inflammation correlates with poAF risk, but less is known about the cellular and molecular mechanisms driving postoperative atrial arrhythmogenesis. We performed single-cell RNA-seq comparing atrial nonmyocytes from mice with and without poAF, which revealed infiltrating CCR2^+^ macrophages to be the most altered cell type. Pseudotime trajectory analyses identified *Il-6* as a gene of interest driving in macrophages, which we confirmed in pericardial fluid collected from human patients after cardiac surgery. Indeed, macrophage depletion and macrophage-specific *Il6ra* conditional knockout (cKO) prevented poAF in mice. Downstream STAT3 inhibition with TTI-101 and cardiomyocyte-specific *Stat3* cKO rescued poAF, indicating a proarrhythmogenic role of STAT3 in poAF development. Confocal imaging in isolated atrial cardiomyocytes (ACMs) uncovered what we believe to be a novel link between STAT3 and CaMKII-mediated ryanodine receptor–2 (RyR2)-Ser(S)2814 phosphorylation. Indeed, nonphosphorylatable *RyR2^S2814A^* mice were protected from poAF, and CaMKII inhibition prevented arrhythmogenic Ca^2+^ mishandling in ACMs from mice with poAF. Altogether, we provide multiomic, biochemical, and functional evidence from mice and humans that IL-6-STAT3-CaMKII signaling driven by infiltrating atrial macrophages is a pivotal driver of poAF, which portends therapeutic utility for poAF prevention.

## Abstract 2

This study revealed that macrophages that infiltrate the atria following surgery release interleukin-6 which causes aberrant calcium release events and postoperative atrial fibrillation.

## Introduction

Postoperative atrial fibrillation (poAF) is transient AF that occurs most commonly within 2–4 days after cardiac surgery in 33% of patients (1) despite beta-blocker prophylaxis (2, 3). While transient, poAF increases the risk of stroke and mortality by 50% (4) and recurrent AF by 8-fold, indicating that current therapies do not adequately treat the postsurgical inflammatory changes that may lead to long-lasting sequelae (5). The degree of postsurgical inflammation directly correlates with poAF risk (6, 7), and we previously demonstrated greater NLRP3 inflammasome activity in the atria of poAF compared with sinus rhythm patients (8).

IL-6 is a key inflammatory cytokine downstream of the NLRP3 inflammasome that was also elevated in the sera (9) and atria (7) of patients with poAF, although these studies were focused on samples collected at the time of surgery and thus reflect the contribution of preexisting substrate rather than postsurgical inflammation. We and others have shown IL-6 to be elevated in poAF animal models at the time of arrhythmia on postoperative day 3 (10–13). However, less is known about the key cell type(s) involved in IL-6 signaling or how it mechanistically promotes atrial arrhythmogenesis. Biologically, IL-6 activates the JAK/STAT3 cascade upon binding to transmembrane subunit glycoprotein 130 (gp130) and the IL-6 receptor α (IL-6Rα), which is expressed by hepatocytes and leukocytes, predominantly of the myeloid lineage (14). Therefore, IL-6 signaling in most cell types (i.e., atrial cardiomyocytes [ACMs]) requires the presence of nearby leukocytes to generate IL-6Rα (15) through ADAM 10- and 17-mediated ectodomain cleavage in a process known as transsignaling (16).

Here, we demonstrate that infiltrating atrial macrophages are critical for IL-6 signaling in poAF through IL-6Rα shedding. We validated our findings in pericardial fluid samples collected from human patients after open-heart surgery as a surrogate of local cardiac inflammation, which addresses the fundamental limitation of prior studies relying on atrial samples harvested at the time of surgery. Downstream of IL-6Rα, STAT3 inhibition with the FDA-orphan drug designated phosphorylated-STAT3 inhibitor TTI-101 was sufficient to prevent poAF in mice through attenuation of STAT3-CaMKII signaling and downstream triggered activity driven by CaMKII-mediated RyR2-S2814 phosphorylation and arrhythmogenic sarcoplasmic reticulum (SR) Ca^2+^ leak.

## Results

### Macrophages are necessary for poAF.

**Figure 1:**
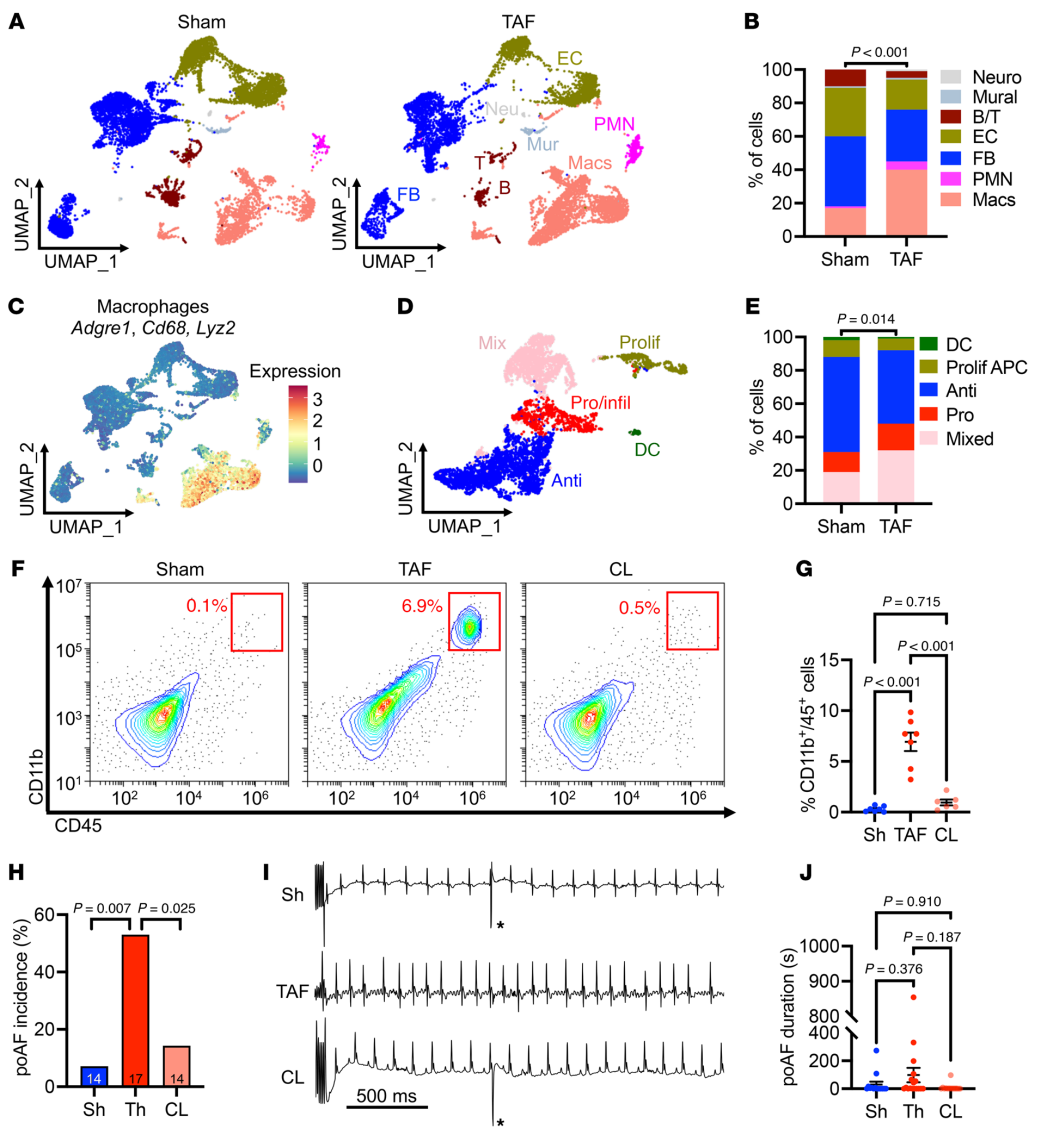
Single-cell landscape of poAF. (**A**) UMAP plots and (**B**) cell distributions of scRNAseq analysis of atrial nonmyocytes from sham (*n* = 3 mice, 9,090 cells) and TAF (*n* = 3 mice, 8,596 cells) mice. (**C**) Macrophage annotation and (**D**) reclustering of macrophages (*n* = 4,946 cells) with (**E**) quantification of macrophage subtypes. (**F**) Flow cytometry with (**G**) quantification showing increased atrial CD11b^+^ macrophages in TAF versus sham mice that was reversed by CL. Each dot represents 1 mouse. (**H**) Incidence of poAF was decreased after CL macrophage depletion. Number of mice per group is depicted. (**I**) Sample ECG tracings after atrial burst pacing. (**J**) poAF duration in Sh, TAF, and CL mice. Please note that these data show that macrophages are the most prominent cell type altered in poAF, and that macrophage depletion prevented poAF. *P* value in **B** was obtained from χ^2^ test comparing the proportion of macrophages in Sham versus TAF. *P* value in **E** was obtained from χ^2^ test comparing the proportion of proinflammatory/mixed versus nonproinflammatory/mixed macrophage subtypes in Sham versus TAF. *P* values in (**G** and **J**) were obtained from 1-way ANOVA followed by Tukey’s posthoc tests at α = 0.05. *P* values in **H** were obtained from χ^2^ tests comparing the proportion of positive poAF events. CL, clodronate liposome; Anti, anti-inflammatory; B, B cell; DC, dendritic cell, EC, endothelial cell; ECG, electrocardiogram; FB, fibroblast; Mac, macrophages; Mur, mural; PMN, polymorphonuclear neutrophil; poAF, postoperative atrial fibrillation; Pro/infil, proinflammatory/infiltrating; Prolif, proliferating; SMC, smooth muscle cell; T, T cell; Th, thoracotomy.

To induce poAF, a thoracotomy protocol previously described in detail (10) was performed while a sham surgery served as a control (see Supplemental Methods, Supplemental Figure 1A; supplemental material available online with this article; https://doi.org/10.1172/JCI187711DS1). All mice subsequently underwent programmed electrical stimulation (PES) using intracardiac catheter burst pacing on postoperative day 3 to identify mice with poAF after thoracotomy (i.e., thoracotomy AF [TAF]) (10). Representative surface and atrial electrograms showing sinus rhythm and poAF in sham and thoracotomy mice, respectively, are shown in Supplemental Figure 1B, with the full poAF episode shown in Supplemental Figure 1C. To assess the cell types involved in poAF pathogenesis, unbiased single-cell RNA-sequencing (scRNAseq) was performed comparing atrial nonmyocytes isolated from sham and TAF mice. UMAP clustering and annotation of cell types with commonly used markers (17–19) (Supplemental Figure 2, A and B) revealed a 2.4-fold increase (*P* < 0.001) in macrophages in the atria of TAF versus sham mice (Figure 1, A–C). IHC staining of mouse heart sections harvested on postoperative day 3 confirmed the presence of macrophages within the atria that was consistently increased in both the right and left atria of TAF versus sham mice (Supplemental Figure 3, A–D) with unaltered ventricular macrophage accumulation (Supplemental Figure 3E). Consistent with the lack of ventricular macrophage infiltration, ventricular tachycardia (VT) inducibility was unchanged 3 days after thoracotomy (Supplemental Figure 4A). Ventricular IL-6 mRNA and protein levels were increased in TAF versus sham mice, reflective of greater systemic inflammation after cardiac surgery, while ventricular IL-6Rα mRNA and protein levels were unaltered in TAF versus sham mice, consistent with the lack of ventricular macrophage accumulation given the selective expression of IL-6Rα in leukocytes (Supplemental Figure 4, B–D) (14). There was no evidence of an adaptive immune response (i.e., unchanged B and T cell numbers) (Figure 1B) or histologic atrial fibrosis in the left or right atria (Supplemental Figure 5). Thus, macrophage-mediated inflammation, not fibrosis, likely drives atrial arrhythmogenesis in our murine poAF model.

To further assess the differential contributions of macrophages to poAF pathogenesis, macrophages from our scRNAseq dataset were reclustered, which separated them into antiinflammatory, proinflammatory/infiltrating, mixed (pro/antiinflammatory), proliferating antigen-presenting cells, and dendritic cells using cell type–specific markers (Figure 1D and Supplemental Figure 2C) (18). Strikingly, TAF mice exhibited a 1.5-fold (*P* = 0.014) increase in proinflammatory and mixed macrophages (Figure 1E). To investigate possible chemoattractants driving this postsurgical increase in atrial macrophages, we assessed differential cell-cell crosstalk using CellChat, which models ligand-receptor interactions derived from scRNAseq data (20). We found a 2.5-fold increase in cell-cell communication pathways in TAF versus sham mice (Supplemental Figure 6, A and B) that was predominantly driven by an increase in macrophage-mediated signaling (Supplemental Figure 6C). A detailed analysis of the altered signaling pathways in macrophages revealed the CCL pathway mediated by CCR2 to be a top differentially upregulated pathway in the atria of TAF versus sham mice (Supplemental Figure 6, D and E). Indeed, flow cytometry confirmed a significant increase in infiltrating CCR2^+^ monocyte-derived macrophages in the atria of TAF versus sham mice (Supplemental Figure 6, F and G). Thus, our data show that chemokine signaling mediated by CCR2 drives the postsurgical infiltration of monocyte-derived macrophages into the atria.

Given the relevance of macrophages in poAF development, we sought to assess whether macrophages are necessary for poAF. To do this, macrophages were depleted in mice by intraperitoneal clodronate liposome (CL) injection 1 hour before cardiothoracic surgery (Supplemental Figure 7A), which reduced macrophage counts by 92% in the spleen (*P* < 0.001) 3 days after injection (Supplemental Figure 7B), consistent with prior studies (21). A similar reduction (86% decrease, *P* < 0.001) in atrial CD11b^+^ macrophages was seen using flow cytometry on postoperative day 3 (Figure 1, F and G). Strikingly, there was a 3.7-fold (*P* = 0.025) reduction in poAF inducibility (Figure 1, H and I) in macrophage-depleted versus placebo-treated mice after thoracotomy, with a concomitant trend toward decreased poAF duration (Figure 1J). The average AF frequency in WT mice after thoracotomy was 11.5 Hz, or 682 beats per minute, which is characteristic of the rapid ventricular response often seen in the human condition. There were no changes in electrocardiogram (ECG) parameters including RR, PR, and QRS intervals as well as sinus node recovery time (SNRT) and atrioventricular effective refractory period (AVERP) among groups that might affect poAF inducibility through enhanced sinus node activity and/or reentry-promoting substrate alterations (Table 1 and Supplemental Figure 8A). Thus, monocyte-derived atrial macrophages are fundamentally required for poAF development.

### Macrophages promote poAF through IL-6 signaling.

**Figure 2:**
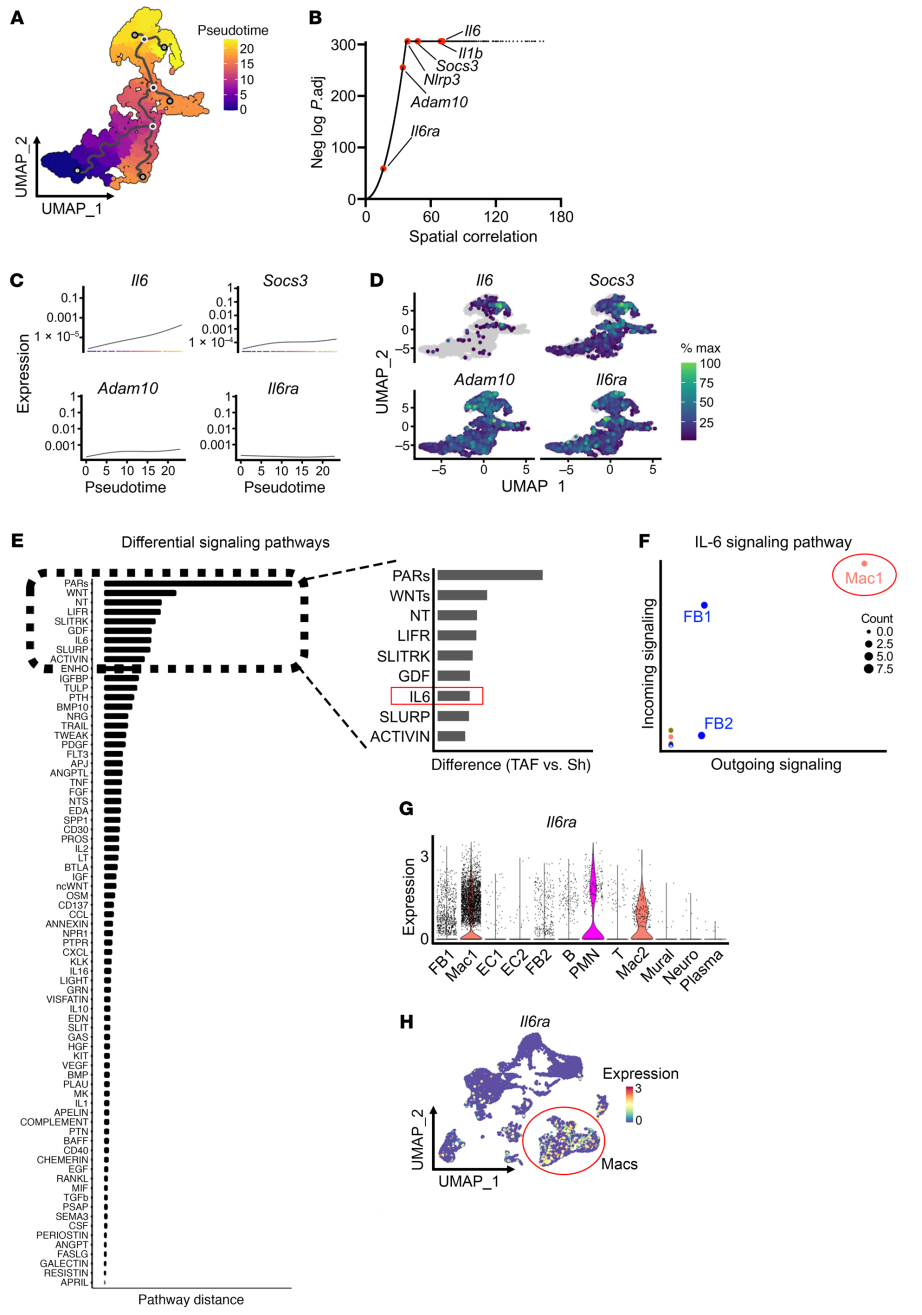
Macrophages promote poAF through IL-6 signaling. (**A**) Monocle 3 pseudotime trajectory analyses were conducted on macrophages, (**B**) which identified *Il6*, *Il1b*, and *Socs3* as top genes changing in pseudotime. (**C**) Corresponding pseudotime-gene expression plots and (**D**) UMAP plots of the top genes changing in pseudotime. (**E**) CellChat analyses conducted using our scRNAseq dataset to identify differential cell-cell interactions in sham versus TAF mice revealed the IL-6 pathway mediated by IL-6 binding IL-6Rα and gp130 to be among the top differentially upregulated pathways. (**F**) Macrophages were the top drivers of IL-6 signaling, (**G** and **H**) in large part through selective expression of *Il6ra*. Please note that these data show that IL-6 is the top cytokine pathway changing in pseudotime in macrophages as well as one of the top differentially upregulated cell-cell communication pathways in TAF versus sham mice. Adam10, A Disintegrin and metalloproteinase domain-containing protein 10; EC, endothelial cell; FB, fibroblast; GDF, growth differentiation factor; Il1b, interleukin 1 β; Il6, interleukin 6; Il6ra, interleukin 6 receptor α; LIFR, leukemia inhibitory factor receptor; Mac, macrophage; Nlrp3, NLR family pyrin domain containing 3; NT, neutrotrophin; PARs, proteinase activated receptors; PMN, polymorphonuclear neutrophil; Sh, sham; SLURP, secreted Ly6/uPAR-related protein; Socs3, suppressor of cytokine signaling 3; TAF, thoracotomy atrial fibrillation.

Given that the prominent macrophage subtype in TAF mice was a mixed pro/antiinflammatory class likely in a transitioning state, we conducted unbiased Monocle 3 pseudotime analyses (Figure 2A), which mathematically models dynamic changes in cell state by calculating cellular trajectories (22), to assess for key driver genes in this macrophage cell state transition. Strikingly, *Il6* was the most correlated cytokine in the macrophage cell state transition, exhibiting greater spatial correlation than *Il-1β* and *Nlrp3* (Figure 2B), which we previously demonstrated to play a role in poAF (8). Importantly, IL-6 signaling counterparts *Socs3*, *Adam10*, and *Il6ra* were also among the top genes driving the antiinflammatory to proinflammatory macrophage cell state transition (Figure 2, B–D). Parallel cell-cell communication analyses comparing differential cell-cell interactions in sham versus TAF mice revealed that IL-6 was indeed a top differentially upregulated signaling pathway in TAF versus sham mice (Figure 2E). Upon further analysis of the IL-6 pathway, we found that macrophages were the most prominent drivers of IL-6 signaling (Figure 2F), likely through expression of *Il6ra* (Figure 2, G and H). Thus, IL-6 signaling driven by atrial macrophages is central to poAF.

### IL-6 signaling is increased in poAF.

**Figure 3:**
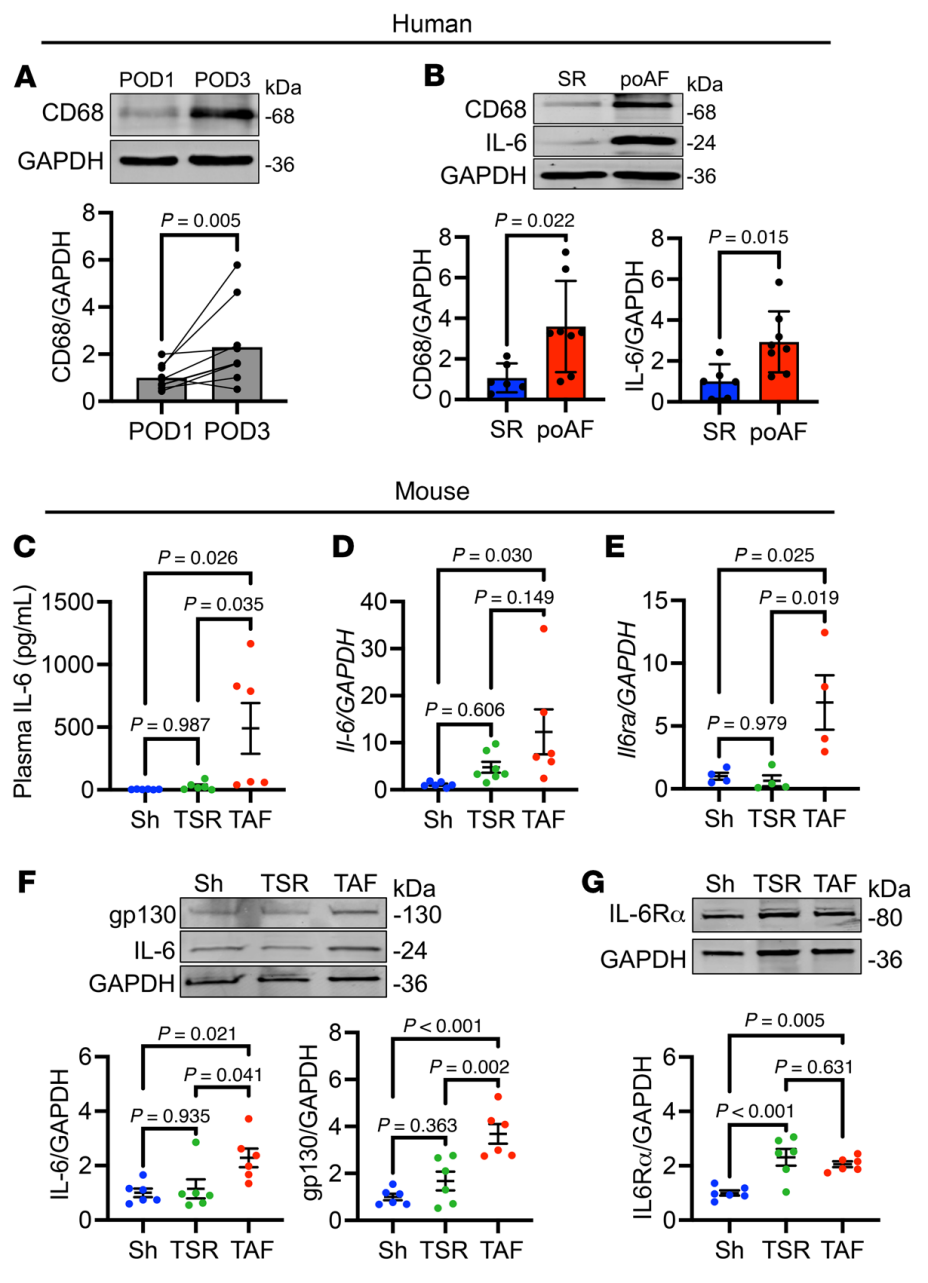
Atrial IL-6 signaling is increased in poAF. (**A**) Paired human PF samples collected on POD 1 and POD 3 after open-heart surgery underwent Western blotting for CD68. (**B**) Western blotting of human PF samples collected on POD 3 revealed increase CD68 and IL-6 protein levels in patients with poAF versus sinus rhythm. Each dot in **A** and **B** represents 1 patient. (**C**) Serum IL-6 was increased in plasma from TAF compared to TSR and sham mice on POD 3. RT-qPCR from whole atria harvested on POD 3 revealed increased (**D**) *Il6* and (**E**) *Il6ra* mRNA in TAF versus TSR and sham mice. (**F**) Western blotting revealed increased IL-6 and gp130 protein levels within the atria of TAF mice. (**G**) While both greater than sham, TAF and TSR mice exhibited no differences in IL-6Rα protein levels despite greater mRNA levels, suggesting active IL-6Rα shedding in TAF mice. Each dot in **C**–**G** represents 1 mouse. Please note that these data show that macrophages and IL-6 are greater in human patients and mice with poAF than without poAF. *P* value in **A** was from paired 2-tailed *t* test. *P* value in **B** was from 2-sample *t* test. *P* values in **C**–**G** were from 1-way ANOVA followed by Tukey’s post hoc test at α = 0.05. IL-6, interleukin 6; IL-6Rα, interleukin 6 receptor α; PES, programmed electrical stimulation; PF, pericardial fluid; POD, postoperative day; SR, sinus rhythm; TAF, thoracotomy atrial fibrillation; TSR, thoracotomy sinus rhythm.

To assess the translatability of our findings to the human condition, we collected pericardial fluid from patients after open-heart surgery as a surrogate measure of local cardiac inflammatory activity (as cardiac biopsy is not possible during the postoperative period). We first assessed whether macrophages infiltrated human hearts after cardiac surgery by collecting paired pericardial fluid samples from the same patients on postoperative days 1 and 3. Strikingly, CD68 protein levels, indicative of macrophages, were 2.1-fold (*P* = 0.005) greater on postoperative day 3 than day 1, indicating that macrophages do indeed infiltrate human hearts after cardiac surgery (Figure 3A). We then focused on pericardial fluid collected on postoperative day 3, which is the time of peak poAF occurrence, and found that patients with poAF had 3.6-fold (*P* = 0.022) and 2.9-fold (*P* = 0.015) greater CD68 and IL-6 protein, respectively, compared with patients in sinus rhythm (Figure 3B). Apart from rhythm status, there were no differences in clinical characteristics between patients with poAF and sinus rhythm (Supplemental Table 4).

Given our findings implicating altered macrophage-mediated IL-6 in human poAF, we assessed the atria for changes in IL-6 and its signaling counterparts in our poAF mouse model (10). After PES on postoperative day 3, mice were divided into sham (Sh), mice that underwent surgery but remained in sinus rhythm (thoracotomy sinus rhythm [TSR]), and mice with poAF (TAF). Plasma IL-6 and atrial *Il6* and *Il6ra* were greatest in TAF compared with TSR and sham mice (Figure 3, C–E). Consistent with a systemic inflammatory IL-6 response, ventricular IL-6 was increased at the mRNA and protein levels while ventricular IL-6Rα remained unchanged, reflecting the lack of ventricular macrophage infiltration (Supplemental Figure 3, D–F). Atrial IL-6 was significantly greater in TAF compared with TSR (2.0-fold, *P* = 0.041) and sham (2.29-fold, *P* = 0.026) mice (Figure 3F). Atrial gp130 protein levels were also increased in TAF versus TSR (2.2-fold, *P* = 0.002) and sham (3.7-fold, *P* < 0.001) mice. Interestingly, there were no differences in IL-6Rα between TAF and TSR (Figure 3G), in contrast with what was observed at the *Il6ra* mRNA level (Figure 3E). As IL-6Rα protein levels by Western blot were indicative of membrane-bound IL-6Rα, these results suggest an increase in shedding of membrane-bound IL-6Rα and greater proinflammatory IL-6 transsignaling. Indeed, atrial ADAM17 and ADAM10, the primary IL-6Rα sheddases (23), were greatest in TAF compared with TSR and sham mice at both the mRNA and protein levels (Supplemental Figure 9). Taken together, IL-6 and mediators of IL-6 transsignaling were greatest in TAF compared with sham and TSR mice. Even among age- and sex-matched mice that underwent the same cardiac surgery, only those with poAF (TAF) exhibited increased atrial IL-6, indicating that the postsurgical increase in atrial IL-6 is likely a direct cause of poAF rather than nonspecific consequence of postsurgical inflammation.

To assess the potential relevance of a preexisting IL-6–promoting substrate in poAF development, biochemical studies were conducted from right atrial appendages harvested at the time of cardiac surgery from patients who remained in sinus rhythm and those who developed poAF. The incidence of poAF, defined by a documented AF episode lasting at least 30 seconds (24), was monitored for up to 5-days after surgery. Clinical and demographic characteristics of patients who developed poAF versus sinus rhythm controls were similar (Supplemental Tables 5–7), with the exception of an association between older age and poAF status. There was a nominal increase of *IL6* mRNA and significant increase in IL-6 protein in the atria of patients with poAF versus sinus rhythm at the time of surgery (Supplemental Figure 10, A and B). Due to the association between older age and poAF, we performed linear regression between age and IL-6 protein levels and found a nominally positive association (R^2^ = 0.157, *P* = 0.084; Supplemental Figure 10C), indicating that the greater IL-6 protein levels at the time of surgery in poAF versus sinus rhythm patients is, in part, attributable to the older age of patients with poAF versus sinus rhythm. In contrast, IL-6 protein levels in pericardial fluid collected at the time of arrhythmia exhibited a much greater magnitude of increase in patients with poAF versus sinus rhythm (Figure 3B), indicating that postsurgical changes in IL-6 are more pathologically important than preexisting alterations. Like IL-6, the gp130 transmembrane subunit of the IL-6/IL-6Rα complex was elevated in the atria of patients with poAF compared with sinus rhythm (Supplemental Figure 10, G and H), although there was a trend between older age and atrial gp130 levels (R^2^ = 0.130, *P* = 0.142; Supplemental Figure 10I). Importantly, IL-6Rα was unchanged in human atrial biopsies at the time of surgery (Supplemental Figure 10, D and E) without an age correlation (R^2^ = 0.050, *P* = 0.344; Supplemental Figure 10F), indicating that the observed increases in atrial IL-6Rα mRNA and protein measured on postoperative day 3 in mice (Figure 3, E and G) occur after surgery. Taken together, differences in preexisting atrial IL-6 are minimal compared with those that occur after surgery. In particular, the significant change in IL-6Rα after surgery compared with that existing prior to cardiac surgery validates the observed changes in atrial immune cell profile given the selective expression of IL-6Rα in macrophages (Figure 2, G and H).

### Loss of IL-6Rα from macrophages is sufficient to rescue poAF.

**Figure 4:**
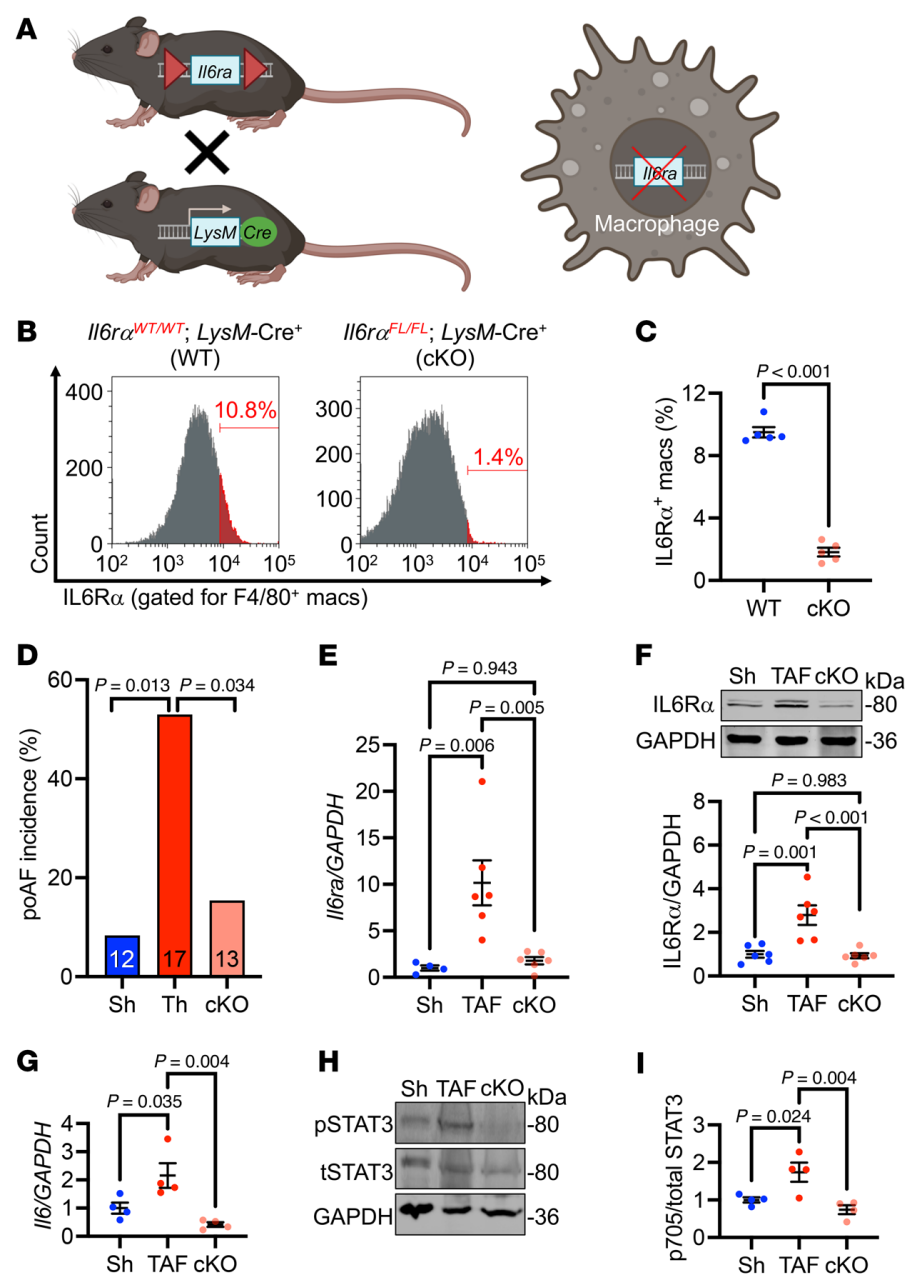
Loss of IL-6Rα from macrophages is sufficient to rescue poAF. (**A**) *Il6ra* was conditionally knocked out from macrophages by expressing Cre under the LysM promoter in *Il6ra*^fl/fl^ mice. (**B** and **C**) Validation of *Il6rα* cKO in macrophages via flow cytometry on mouse splenocytes. Each dot represents 1 mouse. (**D**) Incidence of poAF was decreased in *Il6rα* cKO compared with WT mice after cardiac surgery, with decreased atrial (**E**) *Il6rα* mRNA and (**F**) protein levels. Atrial (**G**) *Il-6* and (**H** and **I**) pY705-STAT3 were lower in *Il6ra* cKO compared with WT mice after thoracotomy, consistent with the hypothesis that IL-6Rα from macrophages is critical for IL-6 transsignaling in the atria. Each dot represents 1 mouse. Please note that these data show that selective inhibition of the IL-6 receptor in macrophages is sufficient to prevent poAF. The *P* value in **C** was obtained from a 2-sample 2-tailed *t* test. *P* values in **D** were obtained from χ^2^ tests comparing the proportion of positive poAF events. *P* values in **E**–**I** were obtained from 1-way ANOVA followed by Tukey’s post hoc test at α = 0.05. cKO, conditional knockout; IL-6Rα, interleukin 6 receptor α; Macs, macrophages; Th, thoracotomy; TAF, thoracotomy atrial fibrillation; LysM, lysozyme M; Sh, sham.

To further explore these findings, *Il6ra* was conditionally knocked out in macrophages by crossing *Il6ra^fl/fl^* and LysM-Cre mice (Figure 4A). Decreased IL-6Rα expression in F4/80^+^ splenic macrophages was validated by flow cytometry (Figure 4, B and C). We chose to express under the LysM promoter, which hits approximately 70% of neutrophils, (25) given that we observed neutrophils (PMNs), in addition to macrophages, to express *Il6ra* in the atria (Figure 2G). Thoracotomy followed by PES studies revealed a 71% (*P* = 0.034) reduction in poAF inducibility (Figure 4D), along with a 4.3-fold (*P* = 0.018) and 3.0-fold (*P* < 0.001) reduction in atrial IL-6Rα mRNA and protein, respectively, in *Il6ra*-cKO versus WT mice after thoracotomy (Figure 4, E and F). Importantly, atrial IL-6 and IL-6 pathway activation by STAT3-Y705 phosphorylation were significantly attenuated in *Il6ra* cKO versus WT mice after thoracotomy (Figure 4, G–I), demonstrating that IL-6Rα from macrophages is crucial for atrial IL-6 transsignaling during the perioperative period. There were no differences in baseline ECG parameters, SNRT, or AVERP among groups (Table 2 and Supplemental Figure 8B). These results demonstrate that targeting the IL-6 signaling axis, specifically IL-6Rα, produced by macrophages, is sufficient to rescue poAF.

### STAT3-CaMKII signaling is increased in poAF.

**Figure 5:**
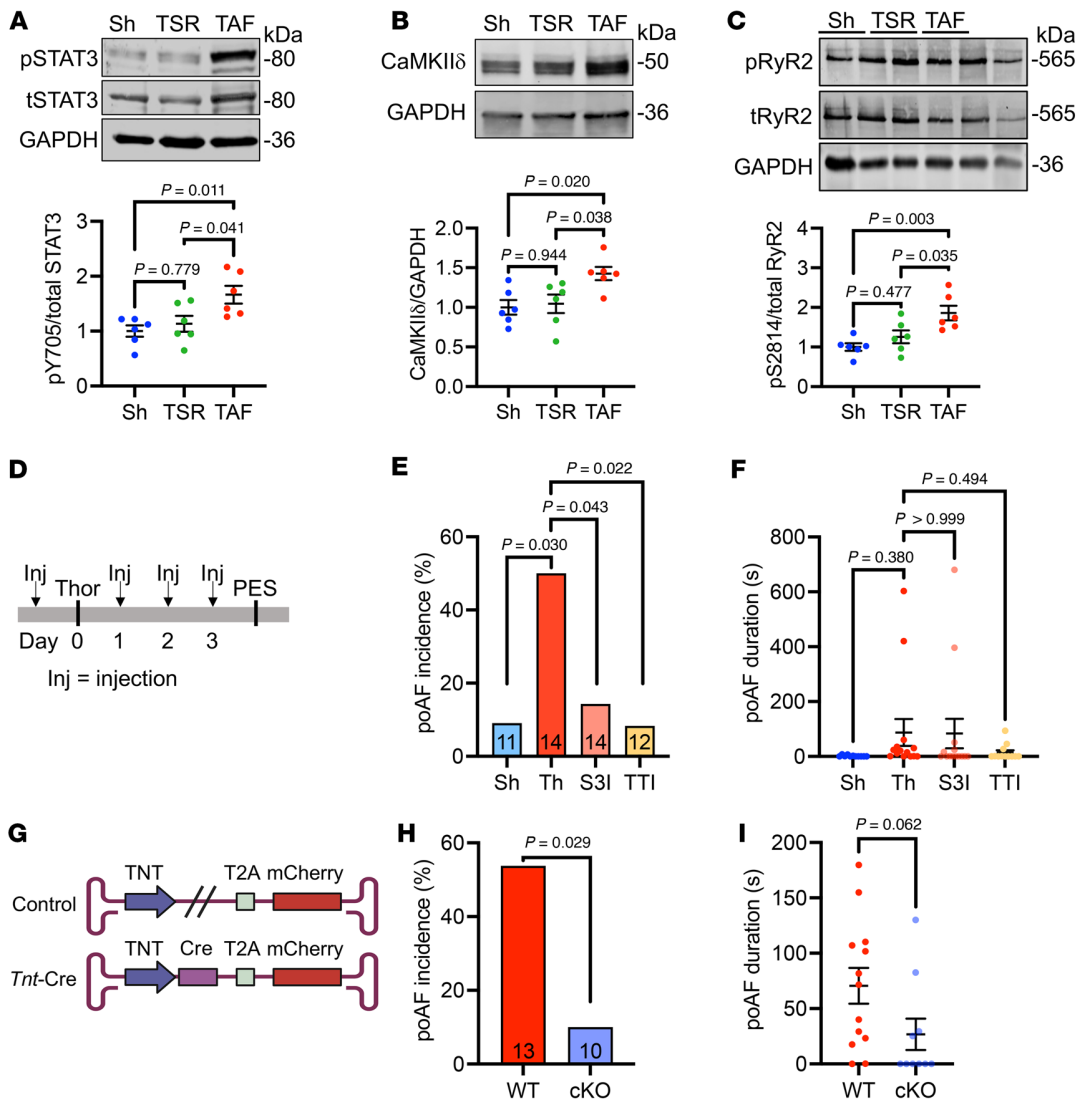
STAT3-CaMKII signaling is enhanced in poAF. Western blotting revealed increased (**A**) STAT3-Y705 phosphorylation, (**B**) CaMKIIδ, and (**C**) RyR2-S2814 phosphorylation in the atria of TAF versus TSR and sham mice. (**D**) STAT3 inhibition with S3I-201 (5 mg/kg) and TTI-101 (100 mg/kg) once a day for 3 days (**E**) reduced poAF incidence, (**F**) with a trend toward reduced poAF duration in the TTI-101 group. (**G**) Cardiomyocyte-specific *Stat3* cKO mice (*n* = 8) were generated by injecting AAV9-*Tnt*-Cre virus into *Stat3^FL/FL^* mice 4 weeks before cardiac surgery. Controls (*n* = 11) consisted of mice injected with control virus 4 weeks prior to cardiac surgery. *Stat3* cKO mice were protected from (**H**) poAF and (**I**) exhibited a nominal reduction in poAF duration. Please note that these data show that, among mice that underwent cardiac surgery, STAT3-CaMKII-RyR2 signaling was greatest in mice that developed poAF. Pharmacologic STAT3 inhibition and cardiomyocyte-specific *Stat3* cKO prevented poAF in mice, indicating that STAT3 plays a direct proarrhythmogenic role in cardiomyocytes. *P* values in **A**–**C** and **F** were from 1-way ANOVA followed by Tukey’s test for multiple correction at α = 0.05. *P* values in **E** and **H** were from χ^2^ tests. *P* value in **I** was from 2-sample 2-tailed *t* test. CaMKIIδ, calcium/calmodulin-dependent protein kinase II δ; cKO, conditional knockout; Inj, injection; PES, programmed electrical stimulation; pRyR2, phospho-RyR2-S2814; pSTAT3, phospho-Tyr705-STAT3; RyR2, ryanodine receptor 2; Sh, sham; SR, sinus rhythm; Thor, thoracotomy; TNT, troponin T; TSR, thoracotomy sinus rhythm; tSTAT3, total STAT3; TTI, TTI-101; TAF, thoracotomy atrial fibrillation; T2A, Thosea asigna virus 2A peptide.

To assess the downstream consequences of enhanced IL-6 signaling in poAF, Western blotting was performed on atrial tissue harvested from mice on postoperative day 3. Consistent with enhanced IL-6 signaling, a significant induction of STAT3 activation by STAT3-Y705 phosphorylation was noted in the atria of TAF compared with TSR and sham mice (Figure 5A). As we and others have previously shown that CaMKII-mediated RyR2 phosphorylation at Ser2814 is upregulated in patients with poAF (8, 11), we hypothesized that STAT3 downstream of IL-6 acts as a transcriptional activator of CaMKIIδ within cardiomyocytes, a biological pathway that was previously reported in endothelial cells (26). Consistent with this hypothesis, we found significantly greater CaMKIIδ protein and CaMKII-mediated RyR2-Ser(S)2814 phosphorylation (27) in the atria of TAF versus TSR and sham mice (Figure 5, B and C), suggesting that STAT3 activation downstream of IL-6 directly promotes proarrhythmogenic alterations in the atria.

To assess the importance of STAT3 in poAF, we pharmacologically inhibited STAT3 using S3I-201 and TTI-101, which is an FDA-orphan drug designated inhibitor of phosphorylated STAT3 (28, 29). We administered TTI-101 or S3I-201 1 hour before surgery and then once a day for 3 days until the assessment of poAF inducibility on postoperative day 3 (Figure 5D). Both STAT3 inhibitors effectively blunted STAT3-Y705 phosphorylation within the atria of mice after thoracotomy on postoperative day 3 (Supplemental Figure 11, A and B). STAT3 inhibition with S3I-201 decreased poAF inducibility by 3.5-fold (*P* = 0.043), whereas a robust 6.0-fold (*P* = 0.022) reduction in poAF incidence was observed after TTI-101 treatment (Figure 5E) without significant changes in poAF duration (Figure 5F). There were no changes in baseline ECG parameters, SNRT, or AVERP among groups (Table 3) that might affect these results.

To directly demonstrate that STAT3 activation within cardiomyocytes plays a proarrhythmogenic and fundamental role in poAF development, we generated cardiomyocyte-specific *Stat3* cKO mice by expressing Cre under the *Tnt* promoter via AAV9 in *Stat3*^FL/FL^ mice (Figure 5G). Effective *Stat3* knockdown was validated by Western blot showing a 1.9-fold (*P* = 0.017) decrease in atrial STAT3 (Supplemental Figure 11C). Strikingly, phospho-Y705 (i.e., activated) STAT3 exhibited a stronger decrease than total STAT3, suggesting that the major pool of activated STAT3 after thoracotomy lies within ACMs. Indeed, these *Stat3* cKO mice were protected from poAF incidence (5.4-fold reduction, *P* = 0.029; Figure 5H) and duration (2.6-fold decrease, *P* = 0.062; Figure 5I). No significant differences were observed in baseline ECG parameters (Supplemental Table 8) or inducibility of ventricular arrhythmias (data not shown). Taken together, IL-6 upregulates CaMKIIδ in a STAT3-dependent manner in cardiomyocytes, leading to RyR2-S2814 phosphorylation and triggered activity.

### IL-6 promotes arrhythmogenic RyR2-mediated SR Ca^2+^ leak.

**Figure 6:**
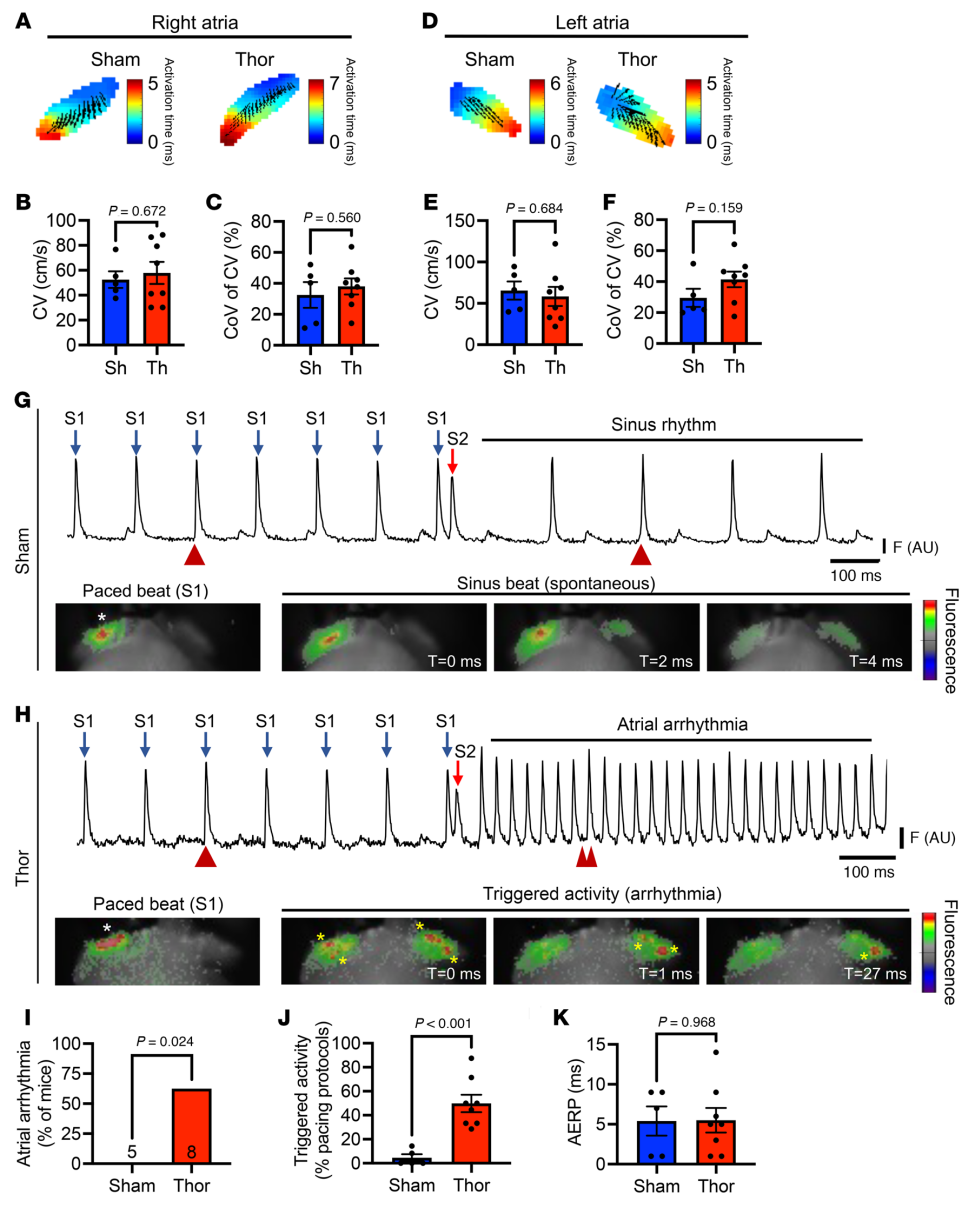
Optical mapping of mouse hearts in sham and thoracotomy mice. Langendorff-perfused mouse hearts from sham (*n* = 5) and thoracotomy (*n* = 8) mice were paced at 10 Hz, and conduction velocity and coefficient of variation were assessed in the (**A**–**C**) right and (**D**–**F**) left atria. Induction of atrial arrhythmias was assessed using an S1-S2 pacing protocol (see Supplemental Materials). Representative atrial electrograms and corresponding voltage-sensitive dye oscillations during and after S1-S2 pacing in (**G**) Sham and (**H**) thoracotomy mice. Blue arrows in **G** and **H** denote S1 pacing at 10 Hz while red arrows denote premature stimulus (S2). Red arrowheads in **G** and **H** denote time stamps at which the images below were taken. White asterisks in paced beat image denotes location of pacing electrode (right atrium) while yellow asterisks in **H** denote triggered activity. (**I**) Atrial arrhythmia incidence, defined as ≥ 2 positive atrial tachyarrhythmia events after S1-S2 pacing, and (**J**) the incidence of triggered activity, defined as the percent of total S1-S2 protocols that led to triggered activity, were significantly greater in thoracotomy versus sham mice. (**K**) AERP did not differ between groups. Please note that these data show that triggered activity is the primary arrhythmia mechanism in our murine poAF mouse model given the lack of reentry-driving substrate alterations. *P* values in **B, C**, **E**, **F**, **J**, and **K** were derived from 2-sample 2-tailed *t* tests. *P* value in **I** was derived from Fisher’s exact test. AERP, atrial effective refractory period; CoV, coefficient of variation; CV, conduction velocity; DADs, delayed afterdepolarizations; Sh, sham; SR, sinus rhythm; Th, thoracotomy.

To definitively show that ectopic (triggered) activity, not reentry, was the predominant driver of atrial arrhythmogenesis in poAF, we conducted optical mapping on ex-vivo Langendorff-perfused mouse hearts. Conduction velocity and coefficient of variation measured at 10 Hz pacing did not significantly differ in thoracotomy versus sham mice in the right (Figure 6, A–C) or left (Figure 6, D–F) atria, consistent with a lack of reentrant-prone substrate alterations. In contrast, we observed a significant increase in triggered activity without evidence of reentry (i.e., rotors) originating from ectopic foci within the atria distant from the pacing site, following S1-S2 pacing (Figure 6, G and H). Consistent with triggered activity as the key driver of atrial arrhythmogenesis in poAF, ex-vivo hearts from mice after thoracotomy had a significantly greater incidence of atrial tachyarrhythmias (Figure 6I) and triggered activity (Figure 6J) without changes in atrial refractoriness (Figure 6K).

**Figure 7:**
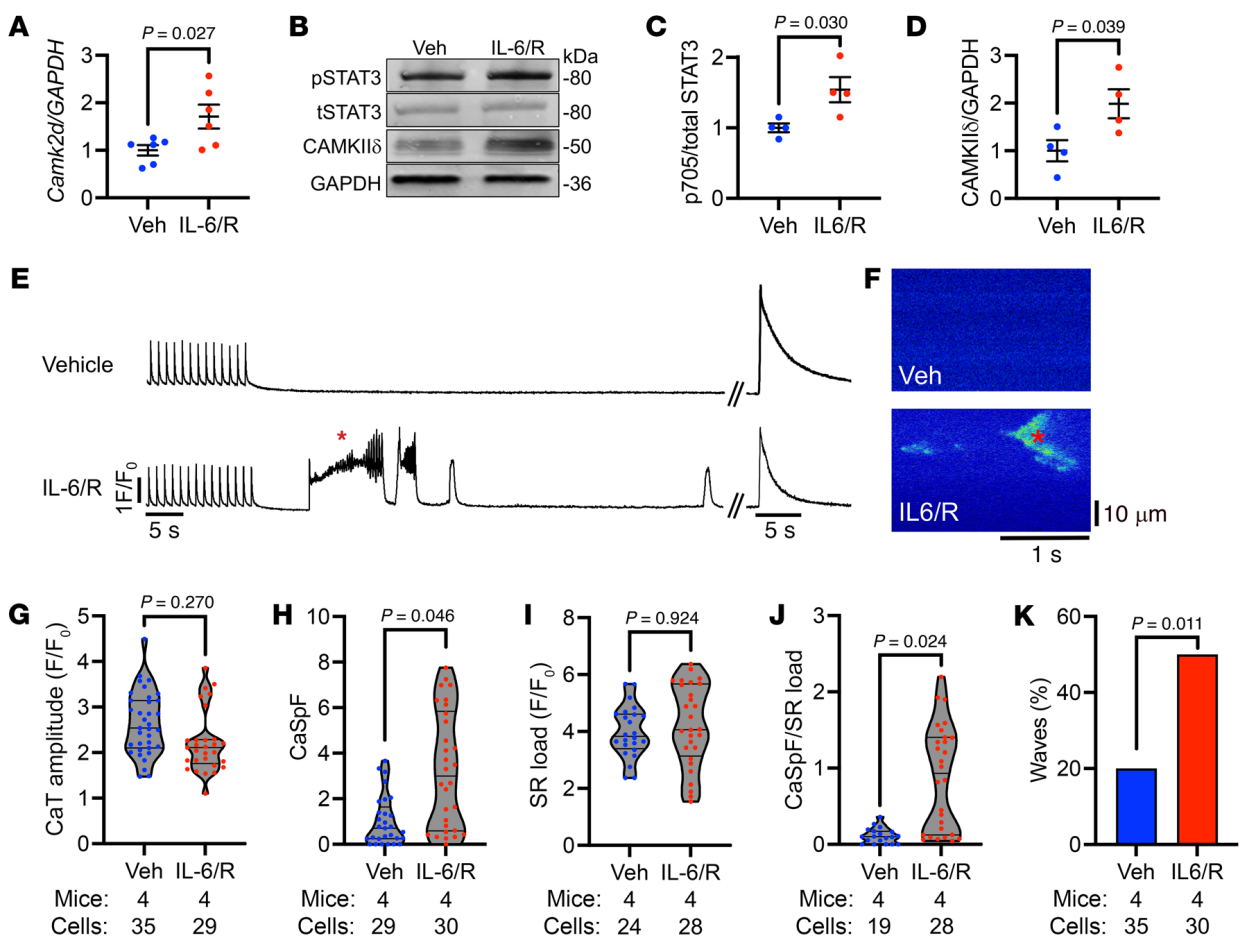
IL-6 is sufficient to induce arrhythmogenic Ca^2+^ mishandling in ACMs. Primary WT mouse cardiomyocytes were incubated with IL-6 (200 ng/mL) + IL-6Rα (100 ng/mL), followed by (**A**) RT-qPCR for *Camk2d* after 30 minutes of treatment and (**B**) Western blotting for (**C**) STAT3-Y705 phosphorylation and (**D**) CaMKIIδ protein after 2 hours of treatment. Atrial cardiomyocytes (ACMs) isolated from WT mice were incubated with IL-6 (200 ng/mL) + IL-6Rα (100 ng/mL) or vehicle (PBS) for 1.5 hours before confocal imaging of SR Ca^2+^ handling. (**E**) ACMs exposed to vehicle (top) or IL-6/R (bottom) were paced at 1-Hz through field stimulation, followed by a 60 second diastolic pause to assess Ca^2+^ sparks and waves prior to rapid 10 mM caffeine exposure to assess SR Ca^2+^ load. (**F**) Representative Ca^2+^ spark images obtained after the pacing train in ACMs exposed to vehicle (top) or IL-6/R (bottom). The asterisk indicates the corresponding Ca^2+^ wave in tracing (**E**) and spark image (**F**). Quantification of (**G**) paced Ca^2+^ transient amplitude, (**H**) CaSpF, (**I**) SR Ca^2+^ load, (**J**) CaSpF/SR Ca^2+^ load, and (**K**) Ca^2+^ wave incidence (cells with waves/total cells) revealing increased CaSpF, CaSpF/SR Ca^2+^ load, and waves after IL-6/R treatment. Number of mice and ACMs are denoted under each figure panel. *P* values in **A**–**D** were obtained from 2-sample 2-tailed *t* tests. *P* values in **G**–**J** were from nested 2-tailed *t* tests to account for clustering of data by mouse and treatment group. *P* value in **K** was from χ^2^ test. Ca^2+^, calcium ion; CaSpF, calcium spark frequency; CaT, calcium transient; IL-6/R, interleukin-6 + interleukin-6 receptor α; SR, sarcoplasmic reticulum; TAF, thoracotomy atrial fibrillation; Veh, vehicle.

Next, to assess the molecular mechanisms by which IL-6 leads to triggered activity, we challenged primary WT mouse cardiomyocytes with IL-6 + IL-6Rα (IL-6/R), which increased *Camk2d* mRNA by 1.71-fold (*P* = 0.027) after 30 minutes (Figure 7A) and CaMKIIδ protein by 1.99-fold (*P* = 0.039) after 2 hours (Figure 7, B–D), consistent with the hypothesized transcriptional upregulation of CaMKIIδ by STAT3. To determine whether this STAT3-mediated CaMKII upregulation was proarrhythmogenic, we performed Ca^2+^ imaging in ACMs isolated from WT mice that were treated with vehicle or IL-6/R for 1.5 hours. ACMs were conditioned by pacing at 1 Hz followed by a 60-second pause during which Ca^2+^ sparks and Ca^2+^ waves, defined as greater than 25% of the Ca^2+^ transient amplitude, were measured. The protocol was finished by assessing SR Ca^2+^ load via acute 10 mM caffeine exposure (Figure 7, E and F). The amplitude of systolic paced Ca^2+^ transients trended lower after IL-6/R treatment (*P* = 0.270, Figure 7G), suggestive of enhanced diastolic Ca^2+^ leak and consistent with prior studies (30). Importantly, there was a 3.2-fold (*P* = 0.046) increase in diastolic Ca^2+^ spark frequency in IL-6/R versus vehicle-treated ACMs (Figure 7H). As SR Ca^2+^ load was unaffected by IL-6/R treatment (*P* = 0.924, Figure 7I), the increase in Ca^2+^ spark frequency remained significant after normalization by SR Ca^2+^ load (*P* = 0.024, Figure 7J). Indeed, the incidence (2.5-fold, *P* = 0.011; Figure 7K) and duration (2.9-fold, *P* = 0.031; Supplemental Figure 12A) of spontaneous Ca^2+^ waves, which are strongly correlated with greater arrhythmogenic potential (31), were greater in IL-6/R versus vehicle-treated ACMs despite no differences in wave latency (Supplemental Figure 12B). Altogether, these results indicate that CaMKII-mediated RyR2 dysfunction was the primary arrhythmogenic driver downstream of IL-6.

### CaMKII inhibition rescues arrhythmogenic SR Ca^2+^ leak.

**Figure 8:**
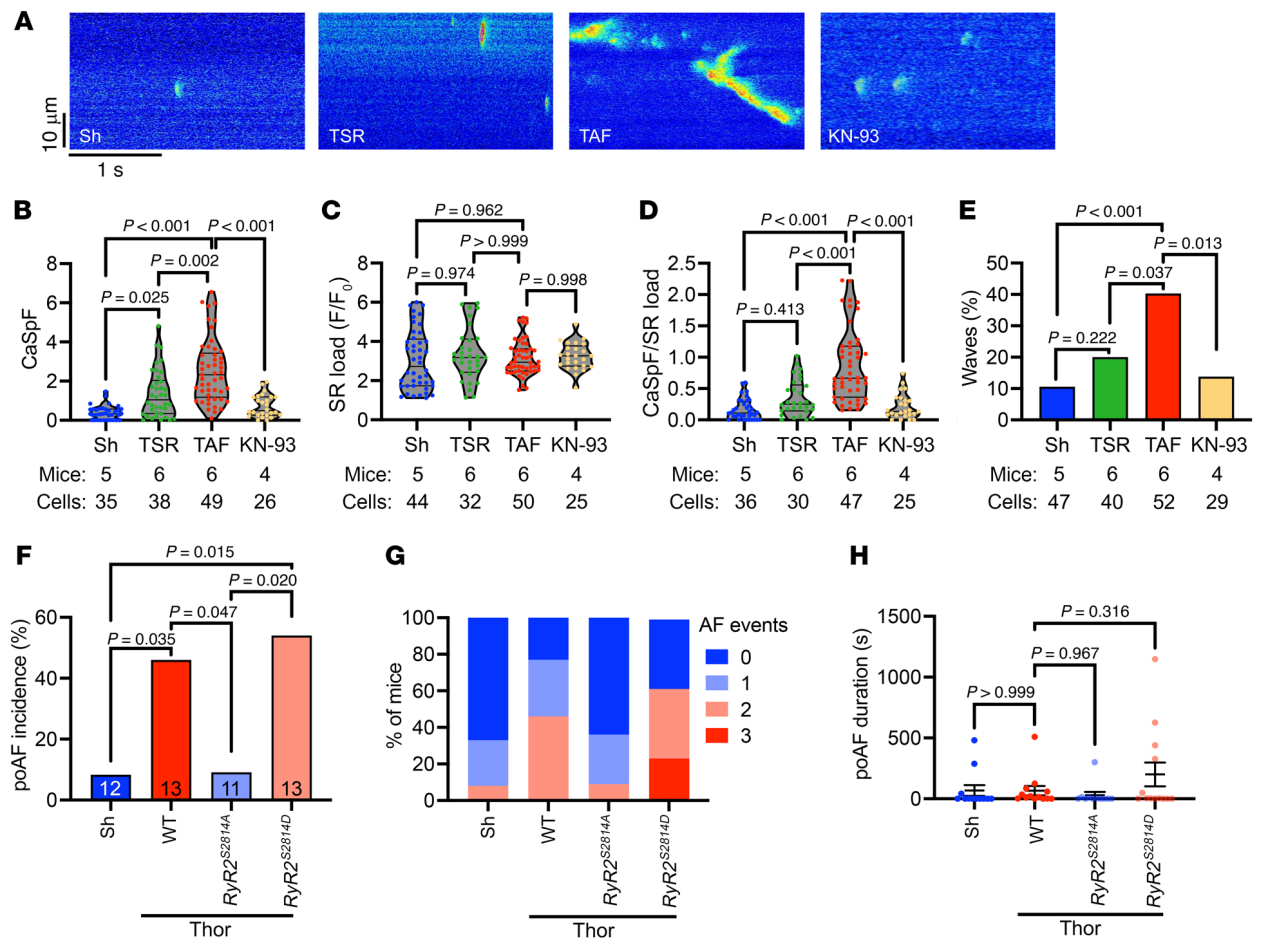
CaMKII inhibition rescues Ca^2+^ mishandling in poAF. ACMs were isolated from sham, TSR, and TAF mice. A subgroup of ACMs from TAF mice were pretreated with CaMKII inhibitor KN-93 for 30 minutes before confocal Ca^2+^ imaging. (**A**) Representative diastolic Ca^2+^ spark images after 1-Hz field pacing of ACMs isolated from Sh, TSR, TAF, and TAF ACMs pretreated with KN-93. Quantification of (**B**) CaSpF, (**C**) SR Ca^2+^ load, (**D**) CaSpF/SR Ca^2+^ load, and (**E**) spontaneous Ca^2+^ wave incidence. Next, nonphosphorylatable *RyR2^S2814A^*, phosphomimetic *RyR2^S2814D^* mice, and WT littermates underwent sham or thoracotomy. (**F**) Incidence of poAF, (**G**) number of poAF events, and (**H**) poAF duration were decreased in *RyR2^S2814A^* compared with WT mice. Please note that these data show TAF ACMs have greater arrhythmogenic Ca^2+^ sparks and waves compared with TSR and that CaMKII inhibition is sufficient to reverse these changes. Indeed, CaMKII phosphorylation at RyR2-S2814 is a necessary action of CaMKII in poAF as *RyR2^S2814A^* were protected from poAF. *P* values in **B**–**E** were from nested 1-way ANOVA to account for clustering of data by mouse and treatment group. *P* value in **F** was from χ^2^ test. *P* value in **H** was from 1-way ANOVA followed by Tukey’s test to correct for multiple testing at α = 0.05. Sh, sham; TSR, thoracotomy sinus rhythm; TAF, thoracotomy atrial fibrillation; Thor, thoracotomy; KN-93, N-[2-[N-(4-chlorocinnamyl)-N-methylaminomethyl]phenyl]-N-(2-hydroxyethyl)-4-methoxybenzenesulfonamide; RyR2, ryanodine receptor type-2.

To assess whether enhanced CaMKII signaling was responsible for the observed changes in ACM Ca^2+^ handling, ACMs were isolated from sham and TAF mice, and confocal imaging of Ca^2+^ handling was performed. Paced Ca^2+^ transient amplitude did not differ among groups (Supplemental Figure 13, A and B), although there was a trend toward decreased transient amplitude after KN-93 treatment as expected with CaMKII inhibition (*P* = 0.677, Supplemental Figure 13B). No differences in Ca^2+^ transient decay were observed among groups, suggesting that alterations in SERCA2a-mediated Ca^2+^ reuptake likely play a minor role in poAF (Supplemental Figure 13C). We then assessed arrhythmogenic diastolic Ca^2+^ sparks after 1-Hz field pacing (Figure 8A). ACMs from TAF mice exhibited 1.9-fold (*P* = 0.002) and 6.6-fold (*P* < 0.001) greater Ca^2+^ spark frequency compared with ACMs from TSR and Sh mice, respectively (Figure 8B). Importantly, KN-93 pretreatment of TAF ACMs was sufficient to reverse these changes (3.5-fold decrease, *P* < 0.001), indicating that CaMKII is necessary for arrhythmogenic Ca^2+^ sparks in poAF (Figure 8B). As SR Ca^2+^ load was unchanged among groups (Figure 8C), similar trends held for Ca^2+^ spark frequency after normalization to SR Ca^2+^ load (Figure 8D), implicating RyR2 Ca^2+^ leak as the primary proarrhythmogenic mechanism in poAF. Indeed, the incidence (Figure 8E) and duration (Supplemental Figure 12C) of spontaneous Ca^2+^ waves were greatest in ACMs from TAF compared with Sh and TSR mice. Interestingly, wave latency was similarly decreased in ACMs from TSR and TAF mice compared with Sh mice, suggesting that faster wave onset may be a nonspecific consequence of cardiac surgery rather than a direct cause of poAF (Supplemental Figure 12D). These findings were unaffected by KN-93 inactive analogue, KN-92, different doses (1 μM versus 2.5 μM) of KN-93 (Supplemental Figure 14, A and B), or pacing at 2 Hz versus 1 Hz (Supplemental Figure 15, A and B). Thus, arrhythmogenic RyR2 Ca^2+^ sparks and waves are directly associated with poAF development as they were absent in ACMs from TSR and Sh mice, and CaMKII inhibition was sufficient to revert this proarrhythmogenic Ca^2+^ mishandling phenotype.

Lastly, to definitively show that RyR2-S2814 phosphorylation by CaMKII and subsequent Ca^2+^ mishandling is necessary for poAF, we performed cardiac surgeries in nonphosphorylatable *RyR2^S2814A^* mice and phosphomimetic *RyR2^S2814D^* mice. Baseline ECG parameters including SNRT and AVERP did not differ among groups, although there was a trend toward faster heart rate in RyR2 phosphomutant mice (Table 4 and Supplemental Figure 8, C and D). Strikingly, nonphosphorylatable *RyR2^S2814A^* mice were protected from poAF compared with their WT littermates (80% decrease, *P* = 0.047; Figure 8, F and G) independent of atrial IL-6 protein levels (Supplemental Figure 16, A and B), demonstrating that RyR2-S2814 phosphorylation is downstream of IL-6. While *RyR2^S2814D^* mice did not exhibit greater poAF inducibility, likely a result of RyR2-S2814 phosphorylation levels already exerting a near-maximal proarrhythmogenic effect after cardiac surgery, these phosphomimetic *RyR2^S2814D^* mice exhibited a trend toward longer poAF duration (mean poAF duration 200s in *RyR2^S2814D^* versus 66s in WT; Figure 8H). Taken together, the phosphorylation of RyR2-S2814 by CaMKII, as opposed to CaMKII effects on other ion channels such as the late sodium current (32, 33), is a necessary action of CaMKII in poAF development.

## Discussion

Our study is the first, to our knowledge, to assess the single-cell transcriptomic landscape of poAF in a clinically relevant mouse model using scRNAseq. Moreover, our studies in pericardial fluid collected from human patients after open-heart surgery address a fundamental knowledge gap in the current understanding of poAF, as prior studies have relied on human atrial tissue collected at the time of surgery (8, 34–37) and thus reflect preexisting substrate rather than the contribution of surgical-induced inflammation, which is more amenable to therapeutic intervention given its acute and transient nature. Our translational findings center around observations after cardiac surgery in human patients and mice and show that infiltrating atrial macrophages are fundamentally required for poAF development through generation of the IL-6 receptor. Downstream of IL-6, STAT3 inhibition with FDA-orphan drug TTI-101 and cardiomyocyte-specific *Stat3* cKO prevented poAF, indicating that activated STAT3 specifically within ACMs plays a direct proarrhythmogenic role in poAF. At the single ACM level, IL-6/R directly induced arrhythmogenic Ca^2+^ mishandling through RyR2 dysfunction in a CaMKII-dependent manner, leading to triggered activity and poAF. Thus, our data suggest that targeting of the IL-6 receptor and downstream signaling in atrial macrophages may represent a novel therapeutic approach for the prevention of poAF.

### Macrophages infiltrating the atria play a central role in poAF.

Our results implicate, in an unbiased manner, infiltrating monocyte-derived macrophages as a central cell type contributing to poAF (Figure 1, A and B and Supplemental Figure 6, F and G). We have previously demonstrated increased macrophages in the atria of patients with and without poAF, although these samples were importantly taken at the time of surgery (8) and thus do not reflect the contribution of surgery-mediated inflammation. We have built upon this prior finding using pericardial fluid collected after cardiac surgery to show that a greater quantity of macrophages infiltrates the heart after cardiac surgery in patients with versus patients without poAF (Figure 3, A and B). Our novel findings in the perioperative period of patients who have undergone cardiac surgery confirm that the results we and others (11, 38) have shown regarding the importance of macrophages in small animal poAF models translates to the human condition.

Furthermore, we build upon the prior literature by demonstrating that macrophage depletion is sufficient to prevent poAF (Figure 1H). We identified CCR2 as a likely upstream chemoattractant receptor driving atrial macrophage infiltration (Supplemental Figure 6, D–G). Indeed, MCP-1, the ligand for CCR2, was increased in the pericardial effluent of patients 48 hours after cardiac surgery (39). Consistent with the role of MCP-1 in macrophage recruitment, we found that macrophages infiltrated human hearts after cardiac surgery to a greater extent in patients with poAF versus sinus rhythm (Figure 3, A and B). Downstream of atrial macrophage infiltration, we utilized pseudotime trajectory analyses to identify *Il-6* as a central regulatory gene of macrophage cell state (Figure 2B), which we confirmed to be upregulated in patients with poAF (Figure 3B). Indeed, CellChat revealed that macrophages were the predominant cell type driving upregulated IL-6 signaling in poAF (Figure 2F). Moreover, by comparing human atrial tissue collected at the time of surgery to mouse atria collected at the time of arrhythmia, we found that IL-6Rα was the only protein in the IL-6 signaling cascade that progressed from unchanged at the time of surgery (Supplemental Figure 10E) to increase at the time of arrhythmia (Figure 3G), suggesting that IL-6Rα may be amenable to therapeutic intervention.

### IL-6 receptor signaling in macrophages is essential for poAF development.

To test whether IL-6 receptor signaling in macrophages is essential for poAF development, we generated macrophage-specific *Il6ra* conditional knockout mice. These *Il6ra-*cKO mice were protected against the development of inducible poAF after thoracotomy (Figure 4G). Indeed, mice that underwent thoracotomy did not exhibit greater ventricular tachycardia inducibility compared with sham mice, consistent with the lack of macrophage-dependent IL-6Rα production and subsequent IL-6 transsignaling (Supplemental Figure 4). Prior studies focused on the ligand (IL-6) and demonstrated that global *Il-6* knockout (13) and IL-6 pharmacologic inhibition (11) protected against poAF in rat sterile pericarditis models (Supplemental Table 9). However, these studies, in particular global *Il6* knockout, are confounded by indirect proarrhythmogenic alterations such as metabolic dysfunction (14, 40). Moreover, nonspecific inhibition of IL-6 blunts classical IL-6 signaling, which is antiinflammatory, and thus may aggravate the proinflammatory postsurgical response, making this approach clinically undesirable (41). Indeed, a recent preclinical study of chronic AF in mice demonstrated that selective neutralization of IL-6 transsignaling via soluble gp130 ameliorated AF inducibility induced by chronic pressure overload (42). However, this paper focused on the role of IL-6 signaling in chronic atrial substrate alterations through connexin and fibrotic remodeling, which our data did not reveal to change in our 72-hour poAF mouse model (Supplemental Figure 5 and Supplemental Table 9).

IL-6 receptor signaling involves the STAT3 pathway, which was elevated in TAF mice compared with those that underwent cardiac surgery but remained in sinus rhythm (i.e., TSR; Figure 5A). We then performed in vivo studies to test the hypothesis that STAT3 inhibition could prevent poAF in our mouse model. Indeed, our studies revealed that STAT3 inhibition with TTI-101, an inhibitor of phosphorylated STAT3 (28), prevented poAF in mice (Figure 5E). These findings suggest that drug repurposing might be a future option for TTI-101, which is currently in phase 2 clinical trials for hepatocellular carcinoma (NCT05440708), metastatic breast cancer (NCT05384119), and idiopathic pulmonary fibrosis (NCT05671835).

### IL-6 receptor-dependent arrhythmogenic mechanisms in poAF.

STAT3 is a transcription factor, widely known for its role in fibrosis in the heart (12). Nonetheless, consistent with prior findings in patients with poAF (8) and animal models (11), we did not find evidence of a change in fibroblast proliferation (Figure 1, A and B) nor evidence of histologic fibrosis (Supplemental Figure 5) in our poAF mouse model. Regardless, the use of antifibrotic therapies in patients during the perioperative period would likely be contraindicated due to impaired wound healing and risk of infection (43, 44). Taken together with the lack of changes in atrial conduction velocity in mice after thoracotomy versus sham surgery (Figure 6, A–F), our data show that reentry is unlikely to be the primary arrhythmogenic mechanism in poAF, although our optical mapping studies were limited by 2-dimensional recordings and we did not pace at supraphysiologic frequencies, which may have been necessary to uncover heterogenous conduction. Nonetheless, our data point toward triggered activity as the primary driver of atrial arrhythmogenesis in poAF (Figure 6, G–J). Molecularly, we found that IL-6–mediated STAT3 activation plays a direct proarrhythmogenic role in cardiomyocytes through CaMKIIδ upregulation (Figure 7, A–D), as cardiomyocyte-specific *Stat3* cKO prevented poAF in mice (Figure 5H). CaMKIIδ is the predominant CaMKII isoform in the heart (45), which is activated during the normal cardiac stress response (46, 47). When overactive, however, CaMKII can cause arrhythmia, particularly AF (48, 49), through RyR2-S2814 hyperphosphorylation (27) and resultant arrhythmogenic Ca^2+^ sparks (50, 51). Importantly, Ruxolitinib, originally discovered as a JAK inhibitor upstream of STAT3, was recently shown to possess CaMKII inhibitory properties and protect against ventricular arrhythmias (52). Our findings implicate enhanced STAT3-CaMKII signaling in poAF development (Figure 5, A–C), thus providing evidence that Ruxolitinib may be a potential candidate for drug repurposing for poAF.

Functionally, we found that IL-6 (or IL-6 receptor activation) recapitulates the Ca^2+^ mishandling observed in poAF, as IL-6/R treatment alone was sufficient to increase SR load-normalized Ca^2+^ sparks and Ca^2+^ waves in isolated ACMs (Figure 7J), pointing to RyR2 dysfunction as the likely arrhythmogenic driver. However, it is important to consider the role of other known drivers of SR Ca^2+^ mishandling such as stress kinase JNK2 (c-Jun N-terminal kinase isoform 2), which is currently being explored a novel gene therapy for primary AF (53). Our results are consistent with prior studies demonstrating cardiac Ca^2+^ mishandling in patients with poAF (8, 54) and in rats (11), although these studies were conducted at the time of arrhythmia or at the whole heart level, respectively, and thus may not completely delineate the molecular Ca^2+^ mishandling phenotype present at the time of arrhythmia at the single ACM level, as we have done in our study (Supplemental Table 9). We further demonstrate that ACMs from TAF mice exhibited significantly greater arrhythmogenic Ca^2+^ sparks and waves compared with those from TSR and that CaMKII inhibition was sufficient to ameliorate these arrhythmogenic changes (Figure 8, B–E). Lastly, to definitely show that the actions of CaMKII on RyR2-S2814 phosphorylation were central to poAF pathophysiology as opposed to CaMKII effects on other ion channels, such as the late sodium current (32, 33), we demonstrated that nonphosphorylatable *RyR2^S2814A^* mice were protected from poAF (Figure 8F). Taken together, our data show that aberrant SR Ca^2+^ release via RyR2-S2814 hyperphosphorylated channels promotes the development of cellular triggered activity in poAF and that these arrhythmogenic events are downstream of IL-6–mediated STAT3 activation in ACMs.

### Potential limitations.

Our study has several limitations. Our studies of human atrial tissue from patients with poAF were cross sectional in nature. Therefore, longitudinal associations between baseline atrial biochemical measurements and incident clinical sequalae could not be derived. We observed a marked induction of *Il6ra* expression in neutrophils as well as macrophages. Although the LysM-Cre transgenic mouse we used reportedly expresses in up to 70% of neutrophils (25), we cannot exclude that the remaining neutrophil-expressed IL-6Rα had subclinical effects on atrial arrhythmogenesis, although we detected a robust reduction in total atrial IL-6Rα mRNA and protein in *Il6ra* cKO mice (Figure 4, E and F) and macrophage-specific *Il6ra* cKO attenuated IL-6 downstream STAT3 activation (Figure 4, H and I). In addition, it is possible that neutrophils activate the IL-6 pathway through indirect effects such as IL-1β–mediated IL-6 activation (55) or activation of Oncostatin-M (56). Future studies would benefit from utilizing neutrophil-specific Cre lines such as Ly6G-Cre to dissect the differential contributions of neutrophils versus macrophages in poAF pathogenesis (25). Indeed, neutrophil-derived myeloperoxidase, in part promoted by reactive oxygen species (57), was shown to promote AF through atrial fibrotic remodeling (58). Moreover, CaMKII activation via oxidation is a well-known driver of AF (59, 60), and we found CaMKII-mediated RyR2-S2814 phosphorylation to play a fundamental role in atrial arrhythmogenesis in our murine poAF model. It is also critical to note that CaMKII phosphorylation of other ion channels, in particular sodium channels (61, 62), could play a key role in poAF initiation and maintenance. In addition, another limitation of our studies is the use of subphysiologic pacing frequencies (i.e., 1–2 Hz) in our confocal Ca^2+^ imaging experiments of isolated ACMs. While these frequencies were sufficient to bring out significant differences in spontaneous Ca^2+^ sparks and waves in ACMs from TAF versus Sham mice (Figure 8, B–E), it is possible that field pacing at more “physiologic” rates (i.e., 10 Hz) could have brought out even greater differences, although the rapid decay of functionality of ex-vivo ACMs limited our ability to conduct such studies. We did not directly assess inflammation-driven fibroblast activation in our poAF mice. While we did not observe differences in histologic atrial fibrosis (Supplemental Figure 5), it is possible that activated fibroblasts may portend proarrhythmogenic effects independent of collagen deposition, such as direct electrical coupling to cardiomyocytes or release of proinflammatory cytokines (63, 64). Lastly, the mice used in our study (12–15 weeks old) were relatively young and disease free, in contrast to human patients that undergo cardiac surgery and often have multiple comorbidities. Thus, poAF in our mouse model required atrial burst pacing to induce episodes of AF, like other preclinical poAF models (11, 12). However, it is important to note that our mouse studies build upon prior studies (11–13) by including the clinically relevant comparison between mice that underwent surgery and remained in sinus rhythm (TSR) versus those that developed poAF (TAF). Most animal studies of poAF (11–13) include 2 groups: sham versus surgery, which prevents parsing out nonspecific consequences of cardiac surgery versus direct causes of poAF. Therefore, while our mouse model may not fully recapitulate the pathophysiology of spontaneous poAF seen in patients, we believe that our results are clinically translatable to the human condition.

### Conclusions.

Taken together, our study demonstrates what we believe to be a novel paradigm of poAF, whereby macrophages infiltrate damaged atria after cardiac surgery, releasing soluble IL-6Rα and driving a surge in IL-6–mediated STAT3 activation within ACMs. STAT3 upregulates CaMKIIδ, leading to phosphorylation of RyR2 at Ser2814, SR Ca^2+^ leak, and ultimately poAF. We believe that our study has the potential to spark the design of future clinical trials and drug repurposing, as we provide robust mechanistic insights on a targetable molecular pathway with current FDA-approved therapies, such as IL-6Rα monoclonal antibodies (Tocilizumab), CaMKII inhibitors (Ruxolitinib), and RyR2 antagonists (flecainide), or investigational drugs such as STAT-3 inhibitor TTI-101, which may be used for poAF prevention and treatment.

## Methods

### Sex as a biological variable.

For all human and murine studies, equal numbers of male and female participants and animals were used when possible. For mice, controls consisted of WT littermates when possible.

### Mouse procedures.

Our mouse model of poAF was designed to recapitulate cardiac surgery as described in detail (see Supplemental Materials) (10). In vivo electrophysiology studies for poAF assessment were performed as previously described (see Supplemental Materials) (10, 65).

### scRNAseq.

scRNAseq comparing atrial nonmyocytes from sham and TAF mice was performed as described in detail in the Supplemental Materials.

### Flow cytometry.

Flow cytometry was conducted as described in detail in the Supplemental Materials using the antibodies list in Supplemental Table 1.

### Mouse plasma collection.

Mouse blood was collected by cardiac puncture with 25G needle/syringe coated with heparin (McKesson Corporation). Whole blood was allowed to equilibrate at room temperature for 30 minutes before spinning at 2000*g* for 15 minutes at 4°C. The supernatant (plasma) was removed and stored at –80°C.

### Real-time quantitative polymerase chain reaction.

Total RNA was isolated from atrial tissues by TRIzol (15596, Life technologies), and 500 μg of RNA was reverse transcribed by iScript (1708841, Bio-Rad). The iTaq Universal SYBR Green Supermix (Thermo Fisher Scientific) with 1 μM primer and diluted cDNA 1:25 was used for the quantitative PCR. Thermocycler conditions were 40 cycles of denaturation at 95°C for 15 seconds and annealing and extension step of 60°C for 60 seconds. The ΔΔCT method was used to calculate relative quantities normalized to GAPDH. Primers used are listed in Supplemental Table 2. Digital PCR (dPCR) analyses of human right atrial appendages was performed as described in the Supplemental Methods.

### Western blotting.

Western blotting was performed as described in detail in the Supplemental Methods using the antibodies listed in Supplemental Table 3.

### IHC.

IHC was performed as described in detail in the Supplemental Materials.

### Ca^2+^ imaging studies.

Ca^2+^ imaging studies in isolated ACMs were performed as described in detail the Supplemental Materials.

### Statistics.

All studies and analyses were performed in a blinded manner when possible. Statistics were performed with Prism version 10.1.1 (GraphPad). Continuous data are expressed as mean ± SEM. *P* values less than 0.05 were considered statistically significant. The statistical test used for each figure panel described within each corresponding figure legend. The tests used were two sample 2-tailed *t* test, Chi-square test, Fisher’s exact test, 1-way ANOVA followed by Tukey’s post-hoc test for multiple correction, nested two sample 2-tailed *t* tests, and nested 1-way ANOVA followed by Tukey’s post-hoc test for multiple correction. Please refer to the Supplemental Methods for more details.

### Study approval.

All animal studies were performed according to protocol and approved by the Institutional Animal Care and Use Committee of Baylor College of Medicine conforming to the Guide for the Care and Use of Laboratory Animals published by the US National Institutes of Health (Publication no. 85-23, revised 1996). *Il6ra^fl/fl^*, *LysM*-Cre, and *Stat3^fl/fl^* mice were acquired from Jackson Labs. All mice included in this study were 12–15 weeks of age and of a C57BL/6J background. Collection of human pericardial fluid was approved by the local institutional review board at the Baylor College of Medicine, Houston, Texas, United States (Protocol #H-46755). Written informed consent to participate in the study was obtained from every human participant prior to pericardial fluid collection. Collection of human right atrial appendages at the time of surgery was approved by the local ethical review board of the University Duisburg-Essen, Germany (Protocol #12-5268-BO). Written informed consent to participate in the study was obtained from each patient prior to cardiac surgery. See the Supplemental Materials for more details on participant selection criteria.

### Data availability.

All data in this study are available from the corresponding author upon reasonable request. Supporting Data Values for each individual data point are available for download. Data from scRNAseq experiments are publicly available in the National Center for Biotechnology Information BioProject Repository (PRJNA1220606).

## Author contributions

JAK, YAS, and XHTW designed the research studies. JAK, YAS, JANG, IO, LL, AP, IAT, MAT, FB, and SZ conducted the experiments. JAK, YAS, JANG, IO, AP, IAT, MT, FB, MK, MGC, LL, and NL acquired data. JAK, YAS, IO, AP, IAT, MAT, FB, and HYS analyzed data. JAK, YAS, YHS, DD, and XHTW wrote the manuscript.

## Supplementary Material

Supplemental data

Unedited blot and gel images

Supporting data values

## Figures and Tables

**Table 3:**
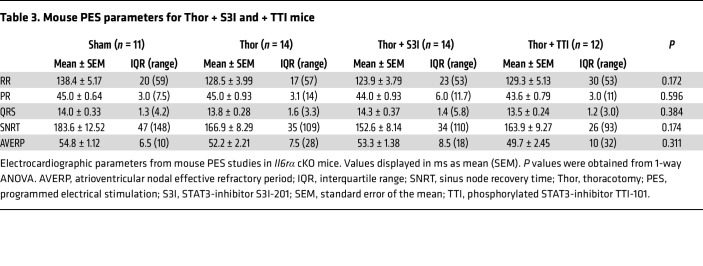
Mouse PES parameters for Thor + S3I and + TTI mice

**Table 4:**
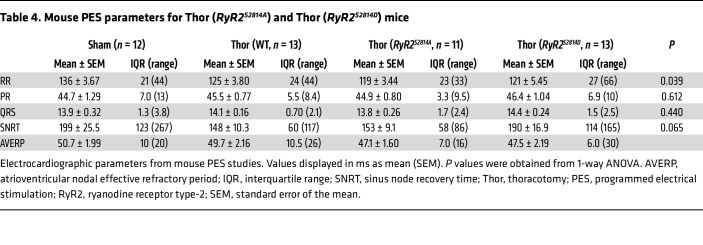
Mouse PES parameters for Thor (*RyR2^S2814A^*) and Thor (*RyR2^S2814D^*) mice

**Table 2:**
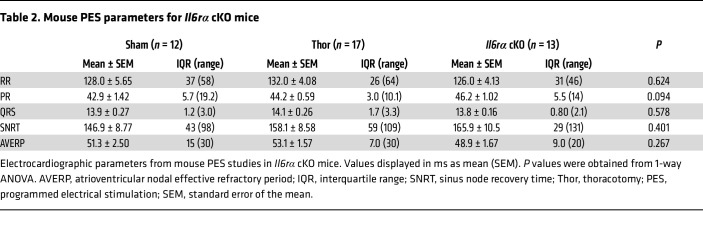
Mouse PES parameters for *Il6rα* cKO mice

**Table 1:**
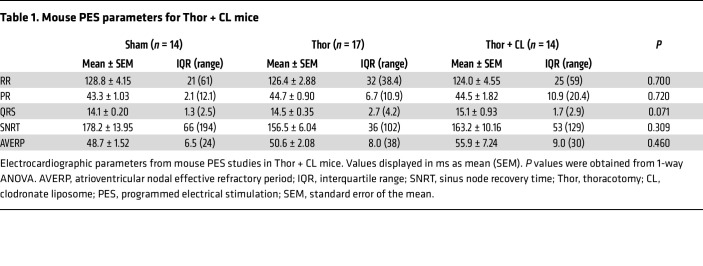
Mouse PES parameters for Thor + CL mice

## Version 1 03/06/2025

In-Press Preview

## Version 2 05/01/2025

Electronic publication
