# Supplementary material for: Macrophage-mediated IL-6 signaling drives ryanodine receptor–2 calcium leak in postoperative atrial fibrillation
Source: J Clin Invest. 2025 Mar 6;135(9):e187711. doi: 10.1172/JCI187711 (PMC12043083; doi:10.1172/JCI187711)
Supplement: Supplemental data [file jci-135-187711-s153.pdf]

Supplemental Material

Macrophage-Mediated Interleukin-6 Signaling Drives Ryanodine Receptor-2 Calcium Leak in Postoperative Atrial Fibrillation

Joshua A. Keefe[1,2], Yuriana Aguilar-Sanchez[1,2], J. Alberto Navarro-Garcia[1,2], Isabelle Ong[1,2], Luge Li[3], Amelie Paasche[1,2,4], Issam Abu-Taha[5], Marcel Tekook[4], Florian Bruns[5], Shuai Zhao[1,2], Markus Kamler[6], H. Ying Shen[7], Mihail G. Chelu[1,8,9], Na Li[1,3], Dobromir Dobrev[2,5,10], Xander H. T. Wehrens[1,2,3,11,12,13]

[1]Cardiovascular Research Institute, [2]Department of Integrative Physiology, [3]Department of Medicine, Baylor College of Medicine, Houston, TX 77030, USA.

[4]Department of Cardiology, Angiology and Pneumology, University Hospital Heidelberg, 69120 Heidelberg, Germany.

[5]Institute of Pharmacology, West German Heart and Vascular Center, University Duisburg-Essen, 45147 Essen, Germany.

[6]Department of Thoracic and Cardiovascular Surgery, West German Heart and Vascular Center Essen, University Hospital Essen, 45122 Essen, Germany.

[7]Department of Surgery, Division of Cardiothoracic Surgery, Baylor College of Medicine, Houston, TX 77030, USA.

[8]Department of Internal Medicine, Division of Cardiology, Baylor College of Medicine, Houston, TX 77030, USA.

[9]Texas Heart Institute at Baylor St. Luke's Medical Center, Houston, TX 77030, USA.

[10]Department of Medicine, Montreal Heart Institute and Université de Montréal, Montreal, Quebec, Canada.

[11]Department of Neuroscience, [12]Department of Pediatrics, [13]Center for Space Medicine, Baylor College of Medicine, Houston, TX 77030, USA.

SUPPLEMENTAL METHODS

Mouse open heart surgery. Open heart surgery was performed as described in detail previously (1). Briefly, mice were given preprocedural analgesia with extended-release buprenorphine (Wedgewood Connect, San Jose, CA) at 1.0 mg/kg at least one hour prior to surgery. Mice were intubated and ventilated at a tidal volume of 150 μL and respiratory rate of 175 breaths/minute. Anesthesia was maintained using 2.5% v/v isoflurane (11695067771, Covetrus, Houston, TX) in 100% oxygen, and body temperature was maintained at 37.0±0.5ºC using a heating pad connected to a rectal thermometer (Rodent Surgical Monitor+, Indus Instruments, Webster, TX). The thoracic cavity was exposed through the 2nd or 3rd intercostal space medial of the midclavicular line. Bi-atrial pericardiectomy and cross-clamping of the thoracic aorta for 20 seconds were performed. For the sham (Sh) procedure, endotracheal intubation and skin and pectoral muscle dissection were performed, but the chest cavity was not entered. Pharmacologic intervention in mice was administered as follows. Clodronate liposome (NC0337390, Thermo Fisher Scientific, Waltham, MA), used to deplete macrophages in mice, was administered intraperitoneally (IP) at 100μL/10g body weight one hour prior to open heart surgery. S3I-201 (573130, Sigma Aldrich, Burlington, MA), a selective STAT3 inhibitor (2), was administered at 5 mg/kg. TTI-101, a selective inhibitor of phosphorylated STAT3 (at Tyr705) (3), was acquired as a generous gift from Tvardi Therapeutics (Houston, TX) and administered at 100 mg/kg. Both S3I-201 and TTI-101 were administered IP one hour prior to surgery and then every 24 hours after surgery for three days. Cardiomyocyte-specific *Stat3* conditional knockout (cKO) mice were generated by injecting homozygous $Stat3^{FL/FL}$ (JAX strain #016923) with adeno-associated virus 9 (AAV9) expressing Cre under the *Tnt* promoter. Controls consisted with age- and sex-matched wildtype mice injected with AAV9-Tnt-Cre-mCherry or AAV9-Tnt-mCherry. All AAV9s were injected 4-weeks prior to cardiothoracic surgery.

Programmed Electrical Stimulation (PES). Electrophysiology studies were performed as previously described (1, 4). Briefly, mice were anesthetized using 2% v/v isoflurane/oxygen, and a 1.1F octopolar catheter (EPR-800, Millar, Houston, TX) was advanced through the right external jugular vein into the right atrium and ventricle. The octopolar catheter leads were connected to an external stimulator in recording mode (STG3008, MultiChannel Systems, Reutlingen, Germany), and the signals together with surface ECG leads were acquired using the IOX2.4 acquisition software (Emka Technologies, Sterling, VA). Proper catheter positioning was verified by the waveforms of the 4 intracardiac electrocardiograms, as previously described (4), and by looking for proper deflections in the P wave after atrial pacing and QRS complex after ventricular pacing. All PES protocols were performed at 1.75% v/v isoflurane/oxygen and rectal temperature between 37.0±0.5°C. Following baseline recordings, the sinus rode recovery time (SNRT) was calculated as the time to first spontaneous sinus beat after right atrial pacing at a basic cycle length of 100 milliseconds (ms) for 15s. The atrioventricular effective refractory period (AVERP) was determined by applying a series of right atrial pacing trains (i.e., S1) at a BCL of 100 ms, after which a premature stimulus known as S2 was applied. The S1-S2 interval was decreased by 2 ms from 70 ms to 20 ms, and the AVERP was defined as the shortest S1-S2 interval in which S2 propagated into the ventricles (resulting in ventricular depolarization). AF inducibility was determined by performing a series of 2s bursts starting at a BCL of 40 ms. The BCL decreased by 2 ms for each 2s burst, starting at a BCL of 40 ms and ending at 20 ms. Burst pacing was performed in triplicate for each mouse, and AF was defined as the presence of an irregularly irregular rhythm without discernable P waves for at least 1 second on at least two out of three atrial burst pacing protocols (1). For assessment of ventricular tachycardia, burst pacing from the ventricular lead was repeated twice at a cycle threshold of 60 ms and twice at a cycle threshold of 40 ms. VT was defined as at least 10 consecutive ventricular beats at rate faster than 600 bpm after any of the four burst pacing protocols (5).

Single-cell RNA sequencing (scRNAseq). Atrial non-myocytes were isolated after PES studies by mechanical and enzymatic digestion. Enzymatic digestion for scRNAseq was achieved via 450 U/mL collagenase II (C2-28, Sigma-Aldrich, St. Louis, MO), 125 U/mL collagenase XI (C7657, Sigma-Aldrich, St. Louis, MO), 60 U/mL DNase (D45131VL, Sigma-Aldrich, St. Louis, MO), and 60 U/mL hyaluronidase (5030-9954, Bio-Rad, Hercules, CA), dissolved in Hanks Balanced Salt Solution (HBSS) with 1.26 mM Ca^{2+} and 0.9 mM Mg^{2+}. Atrial tissue pieces were placed in prewarmed digestion buffer in a water bath at 37.0ºC and triturated every 10 minutes for 30 minutes. The digestion reaction was quenched using 10% FBS in HBSS without Ca^{2+} and Mg^{2+}. Cells were filtered through a 70-µm filter and spun at 340 x g for 7 minutes at 4ºC. Cells were resuspended in RBC lysis buffer for 5 minutes on ice and then spun at 340 x g for 7 minutes at 4 ºC. Cells were resuspended in 5% FBS in HBSS without Ca^{2+} and Mg^{2+}. Single cell suspensions were loaded onto a 10X Genomics Chromium Controller using 10X Single Cell RNA reagents v3.1 (6). DNA libraries were generated following the Chromium scRNA-seq v3.1 protocol. DNA libraries were sequenced using a Next Generation Sequencer NovaSeq 6000, aiming for ~30,000 reads per cell for scRNA-seq libraries. All scRNA-seq, library preparation, and sequencing were performed at the Baylor College of Medicine Single Cell Genome Sequencing Center. To analyze scRNAseq data, raw FASTQ files were demultiplexed and aligned to mouse reference genome (mm10) using Cell Ranger v7.1.0. Count files from Cell Ranger were imported into Seurat for quality control and subsequent analyses as reported in detail (6, 7). Briefly, cells were filtered by >200 unique genes, <10,000 number of RNA molecules, <30% mitochondrial genes, <3% Malat1 expression, and <0.01% Hba expression. Seurat objects were merged, normalized, and integrated using FindIntegrationAnchors. Cell clusters were identified by dimensionality reduction with principal component analysis (RunPCA) and shared nearest neighbor (SNN) analysis and visualized on UMAP plots. After UMAP clustering, a small (n=576 out of 18,262 cells) cluster of low-quality cells resembling atrial myocytes were removed, and the remaining cells were re-clustered to provide higher resolution separation of immune cell clusters. Further characterization of the functional features of macrophage subclusters were based on the enrichment of genes involved in specific functions. Phenotypic

transitions of macrophages from TAF mice were examined by cell trajectory analysis using Monocle 3 v1.3.4 (8). Cell-cell communications of atrial non-myocytes from TAF atria were analyzed using CellChat v1.6.1 (9).

Flow cytometry. Atrial non-myocytes were isolated after terminal PES studies by mechanical and enzymatic digestion with 320 U/mL Collagenase IV (C4-22, Sigma-Aldrich, St. Louis, MO), 1 U/mL Dispase II (D4693, Sigma-Aldrich, St. Louis, MO), and 60 U/mL DNase (D45131VL, Sigma-Aldrich, St. Louis, MO) dissolved in PBS with 0.9 mM Ca^{2+} and 0.5 mM Mg^{2+}. Atria were placed in digestion buffer in a 37ºC water bath and triturated every 5 minutes for 20-30 minutes until sufficient enzymatic digestion was achieved. The digestion reaction was quenched using 2% fetal bovine serum (FBS; TMS-016, Sigma-Aldrich, St. Louis, MO) and 60 U/mL DNase in PBS. Cells were filtered through a 70-μm filter and spun at 340 x g for 7 minutes at 4°C. Red blood cells were lysed (00-4300-54, Thermo Fisher Scientific, Waltham, MA), and cells were incubated in live/dead stain at room temperature for 15 minutes. Cells were resuspended in flow cytometry buffer (0.1% gelatin, 0.05% NaN_3 in PBS without Ca^{2+}/Mg^{2+}) and incubated in 0.01 μg/μL Fc block (553142, BD Biosciences, Franklin Lakes, NJ) for 5 minutes on ice. Fluorophore-conjugated flow antibodies at the appropriate concentrations (**Table S1**) were added to the cell suspension and incubated at 4°C on a shaker for 45 minutes prior to imaging on a Beckman Coulter CytoFLEX flow cytometer. Data were analyzed using CytoFlex software. Debris was gated out using forward and side scatter area, followed by gating for singlets using forward scatter area and height. Compensation was applied using singly-stained controls, and proper gating was determined using fluorescence minus one (FMO) controls.

Digital polymerase chain reaction (dPCR) of human atrial samples. Total RNA was extracted from right atrial appendages (RAA) lysates obtained from sinus rhythm and poAF patients using the RNeasy Mini Kit (74104, Qiagen, Hilden, Germany) according to the manufacturer's instructions. Next,140 ng

RNA was transcribed into cDNA using the reverse transcription kit (4368814, Applied Biosystems, Thermo Fisher Scientific, Waltham, MA) according to the manufacturer's instructions. dPCR was carried out with TaqMan probes from ThermoFisher (**Table S2**). Reactions were run on a QIAcuity Digital PCR System (911001, Qiagen, Hilden, Germany) using a 96-well Nanoplate with 8,500 partitions and the QIAcuity master mix according to the manufacturer's instructions. The following cycling program was used for the reactions: 2 minutes at 50°C, followed by 10 minutes at 95°C, a total of 35 cycles (15s at 95°C and 1 minute at 60°C), and 30s at 60°C. Relative mRNA levels were calculated from copies/µl values and normalized to HMBS, B2M, GATA4, and GAPDH.

Western blotting. Mouse atrial tissue was snap-frozen in liquid nitrogen. 70 µL of RIPA lysis buffer containing 1% CHAPS, Phos-STOP (4906837001, Sigma-Aldrich, St. Louis, MO) and complete mini protease inhibitor cocktail (4693124001, Sigma-Aldrich, St. Louis, MO), 20mM sodium fluoride (NaF), 1mM Na_3VO_4 was added, and atrial tissue was homogenized with steel beads using a homogenizer (Tissue Lyser LT, Qiagen, Germantown, MD) at 50-Hz for 5 minutes. Samples were sonicated 3 times for 2s each and centrifuged at 14,000 rpm for 20 mins at 4ºC. Supernatants were collected as protein lysates, and protein concentration was measured using a NanoDrop spectrophotometer (Thermo Fisher Scientific, Waltham, MA). 60 µg protein was loaded into each well of a 10-12% polyacrylamide gel and run at 100 Volts for ~2-h. Proteins were transferred onto 0.45-µm polyvinylidene fluoride (PVDF) membranes for 16-h at 20 Volts in a 4°C cold room. Membranes were blocked for 1-h at room temperature (20-314, Genesee Scientific, El Cajon, CA) and incubated overnight at 4°C with primary antibodies (**Table S3**) diluted in blocking buffer. Membranes were washed 3 times with TBST (0.1% tween-20) for 10 minutes each and incubated with secondary antibody (**Table S3**) for 1-h at room temperature. After washing 3x10 minutes in TBST, membranes were developed using LICOR Odyssey infrared imager (LICOR, Lincoln, NE). Bands were quantified using the ImageJ software and normalized to GAPDH.

Immunohistochemistry. Mouse hearts were harvested on postoperative day three immediately following PES and cannulated for retrograde perfusion of 30 mM KCl to arrest the heart in diastole. Hearts were fixed in 10% formalin (HT501128, Sigma-Aldrich, St. Louis, MO) for 48 hours, washed in PBS twice for 30 minutes, and stored in 70% ethanol at 4°C. Hearts were embedded in paraffin and sectioned at 7 μm. Sectioned tissue samples were deparaffinized in xylene and underwent gradient ethanol rehydration to dH_2O. Immunohistochemical DAB staining for F4/80 (1:500; D2S9R, Cell Signaling, Danvers, MA) was performed as recommended by the manufacturer. Blocking buffer consisted of 3% normal goat serum, 0.1% BSA, 0.1% Triton X-100 in PBS. Slides were imaged on a Zeiss Axiocam and quantified by using FIJI, and total atrial F4/80+ macrophages were normalized to atrial area quantified using FIJI (10).

Human Pericardial Fluid Samples and Patient Population. All experimental protocols were approved by the Baylor College of Medicine and Baylor St. Luke's Medical Center (BSLMC) Institutional Review Board (IRB, #H-46755). All pericardial fluid samples were collected from patients undergoing open-heart surgery at BSLMC and written informed consent to participate in the study was obtained from every patient. The charts of patients in the cardiovascular intensive care unit were reviewed. Patients aged 18 or older, undergoing open-heart surgery, and with a chest-tube in place for at least 72 hours after surgery were candidates for pericardial fluid collection at 36-72 hours after surgery. Patients with a prior history of atrial fibrillation, on chronic antiarrhythmic drug therapy, undergoing surgery that requires immunosuppression postoperatively (e.g., heart transplant), on immunosuppressive therapy for other conditions, or those with postoperative mediastinal infections were excluded. In addition, samples containing grossly hemolyzed pericardial fluid were excluded from downstream analyses. In total, pericardial fluid samples were acquired from 32 patients, from which age, sex, and comorbidity-matched poAF cases and controls were selected as shown in **Table S4**.

Pericardial fluid was spun for 5 minutes at 800 x *g* at 4C. The supernatant was frozen at -80C and the cell pellet underwent RBC lysis (ThermoFisher #00-4300-54) for 5 minutes on ice. The suspension was spun at 800 x *g* for 5 minutes at 4C prior to storage of the remaining cell pellet at -80C. Protein isolation and western blotting were conducted as described in the Western blotting section. The following western blot antibodies were used: CD163 (Proteintech #16646-1-AP, 1:1000), CD68 (Proteintech #25747-1-AP, 1:1000), CCR2 (GTX109684, 1:1000), IL-6 (Proteintech #21865-1-AP, 1:1000) and GAPDH (Proteintech #60004-1-Ig, 1:5000).

Optical mapping of ex-vivo mouse hearts. Optical mapping was performed as previously described (11). Briefly, mouse hearts were cannulated via the aorta and perfused retrograde and superperfused with Tyrode's solution containing 1.8 mM $CaCl_2$ at 2–5 mL/min. Di4-ANEPPS (Invitrogen, Thermo Fisher Scientific, 0.1 mmol/L, 1 mL) was slowly injected into a drug port to load the heart with the voltage-sensitive dye. Blebbistatin (MilliporeSigma, 6.8 mmol/L, 0.1 mL) was delivered to the heart via the drug port to uncouple electrical and mechanical activation. The emission Vm-signal was fed through a long-pass filter (>700 nm) and acquired via a MiCAM CMOS camera (SciMedia, USA) at a sampling rate of 1 kHz and a pixel size of 100 mm/pixel. Right atrial epicardial pacing and ex-vivo electrograms were recorded by a PowerLab 26 T stimulator (AD Instruments). ElectroMap was used to analyze conduction velocity from the average of 10 consecutive beats at 10 Hz pacing for each mouse. The average conduction velocity of 3 independent 10 Hz pacing trains was taken as the conduction velocity for each mouse. The incidence of atrial arrhythmias was defined on a per-mouse basis as at least two atrial tachyarrhythmias after S1-S2 pacing at a cycle length of 100 ms and S2 intervals of 20 - 1 ms (12). The incidence of delayed afterdepolarizations (DADs) was defined as the percent of pacing S1-S2 pacing trains followed by emergence of triggered activity. Atrial effective refractory period was assessed by S1-S2 pacing as previously described (12).

Mouse atrial cardiomyocyte (ACM) isolation. Mouse hearts were excised, cannulated, and perfused retrogradely using a heated Langendorff system via the aorta, as described (13). The perfusion buffer consisted of Ca^{2+}-free Tyrode solution containing (mM): 140 NaCl, 5.4 KCl, 1 $MgCl_2$, 5 HEPES, and 10 Glucose, pH 7.4, supplemented with 22 µg/mL Liberase TH (5401119001, Sigma-Aldrich, St. Louis, MO). After digesting, hearts were perfused with 5mL Kraft-Brühe (KB) solution containing (mM): 90 KCl, 30 K_2HPO_4, 5 $MgSO_4$, 5 pyruvic acid, 5 B-hydroxybutyric acid, 5 creatine, 20 taurine, 0.5 EGTA, 5 HEPES, 10 glucose, pH 7.2. Atrial appendages were cut, mechanically dissociated, and strained through a 250µm nylon mesh to isolate single cells. Atrial cardiomyocytes (ACMs) were re-adapted to 1.8mM Ca^{2+} as described (14) prior to loading for 30 minutes with 4 µM Cal-520AM (Santa Cruz Biotechnology, Dallas, TX, US) (15). Cells were plated onto laminin-coated (354232, Corning, Corning, NY) coverslips and imaged using an LSM880 confocal microscope (Carl Zeiss, Thornwood, NY) in line scan mode with 1024 pixels per line at 135Hz acquisition using the 40X objective. Functional ACMs, defined by appropriate morphology (i.e., elongated, rectangular) and response to pacing, were paced at 1-Hz or 2-Hz for 30s, unstimulated for 60s, and then perfused with 10mM caffeine to assess SR Ca^{2+} load. Pacing was conducted at sub-physiologic frequencies (i.e., <10 Hz) to prevent metabolic degradation and subsequent reactive oxygen species generation as well as rapid pacing-induced CaMKII activation (16) that would have confounded our measurements. Moreover, our Ca^{2+} confocal experiments are conducted at room temperature, in part to mitigate the temperature-dependent extrusion of Ca^{2+}-sensitive dyes. At such temperatures, the "physiologic" frequencies of isolated ACMs would tend to decrease in comparison to those at body temperature. Ca^{2+} transient amplitude and kinetics were analyzed using pCLAMP10 (Molecular Devices, San Jose, CA). The Ca^{2+} spark frequency (CaSpF) was analyzed using the SparkMaster ImageJ plugin (17). Ca^{2+} waves were defined as spontaneous positive oscillations >25% of the paced Ca^{2+} transient amplitude in the absence of field pacing. In vitro pharmacologic treatment of ACMs was done as follows: wild-type ACMs were incubated with 200 ng/mL IL-6 (406-ML, R&D systems, Minneapolis, MN) and 100 ng/mL IL-6Rα (1830-SR, R&D

systems, Minneapolis, MN). For KN-93 experiments, ACMs from TAF mice were pre-treated in 2.5 μM KN-93 (422711, Sigma-Aldrich, St. Louis, MO) for 30 minutes prior to confocal Ca^{2+} imaging.

Statistical Analysis. Statistics were performed with Prism version 10.1.1 (GraphPad, La Jolla, CA). Continuous data are expressed as mean ± standard error of the mean (SEM). All data points represent biological replicates unless otherwise stated. The D'Agostino-Pearson normality test was used to confirm normality. Unpaired t-tests or ANOVA were used for parametric continuous data, and Mann-Whitney test or Kruskal-Wallis tests were used for non-parametric data. Categorical variables were evaluated with chi-square tests. Fisher's exact tests were used when expected counts were less than 5 in at least 80% of groups. For multiple group comparison, 1-way ANOVA or Kruskal-Wallis followed by Tukey or Dunn's post-hoc tests were used to adjust for multiple testing with alpha=0.05. For Ca^{2+} imaging studies, generalized estimating equations were used to account for clustering of the data by mouse and treatment group. $P<0.05$ was considered statistically significant. Outliers were calculated by the ROUT method (18) with a false discovery rate of 1%. Power analyses were conducted pre-hoc to determine sufficient sample size to detect statistically significant differences at alpha=0.05 with 80% power.

SUPPLEMENTAL TABLES

Table S1. Flow cytometry antibodies.

Antibody	Company	Catalogue #	Concentration	Channel
CCR2 (CD192)	BD Biosciences	747970	0.125 µg/test	BV510
CD11b	ThermoFisher	12-0112-81	0.125 µg/test	PE
CD45	ThermoFisher	15-0451-81	0.06 µg/test	PE-Cy5
F4/80	ThermoFisher	56-4801-80	0.25 µg/test	AF-700
IL6Rα (CD126)	ThermoFisher	12-1261-80	0.125 µg/test	PE
Live/dead	ThermoFisher	L34959	1 µL/300 µL	KO

Table S2. RT-qPCR and dPCR primers.

Primer	Sequence (5'-to-3')
ADAM10 Fwd	GGGGAAGATGGTGTTGCCGA
ADAM10 Rev	GGCACGCTGGTGTTTTTGGT
ADAM17 Fwd	AGAGAGCCATCTGAAGAGTTTGT
ADAM17 Rev	TGGTGCTCTCTTCTCCACGG
Camk2d Fwd	GCTAGGGACCATCAGAAACTG
Camk2d Rev	GTCTTCAAACAGTTCGCCAC
GAPDH Fwd	AGGTCGGTGTGAACGGATTTG
GAPDH Rev	TGTAGACCATGTAGTTGAGGTCA
IL-6 Fwd	TGTGCAATGGCAATTCTGAT
IL-6 Rev	GGTACTCCAGAAGACCAGAGGA
IL6Ra tv2 Fwd	AGGAAGCTTGGCGTTTGGGT
IL6Ra tv2 Rev	CCAAGGAATACGGTGGGGGT
SOCS3 Fwd	ACCTTCAGCTCCAAAAGCGAGTAC
SOCS3 Rev	CGCTCCAGTAGAATCCGCTCTC
gp130 (human samples)	TaqMan probes from ThermoFisher: Hs00174360_m1
IL-6 (human samples)	TaqMan probes from ThermoFisher: Hs00174131_m1
IL-6Ra (human samples)	TaqMan probes from ThermoFisher: Hs01075664_m1

Abbreviations: dPCR – digital polymerase chain reaction, RT-qPCR – reverse-transcription quantitative polymerase chain reaction

Table S3. Western blot antibodies.

Protein	Company	Catalogue #	Dilution	Host
ADAM10	Abcam	ab124695	1,000	rabbit
ADAM17	Sigma	19027	1,000	rabbit
CaMKIIδ	Abcam	ab181052	1,000	rabbit
GAPDH	Proteintech	60004-1-Ig	5,000	mouse
Goat anti-Mouse IgG(H+L) Alexa Fluor™ Plus 680	Invitrogen	A32729	10,000	goat
Goat anti-Rabbit IgG(H+L) Alexa Fluor™ Plus 800	Invitrogen	A32735	10,000	goat
gp130 (mouse samples)	Cell Signaling	3732	1,000	rabbit
IL-6 (mouse samples)	GeneTex	GTX110527	1,000	rabbit
IL-6Rα (mouse samples)	Proteintech	23457-1-AP	1,000	rabbit
RyR2-pS2814	YenZym	Custom	2,000	rabbit
RyR2-total	Invitrogen	MA3-916	1,000	mouse
STAT3-pY705	Cell Signaling	9145	1,000	rabbit
STAT3-total	Cell Signaling	9139	1,000	mouse
gp130 (human samples)	Alomone	ALR-023	500	rabbit
IL-6 (human samples)	Abcam	ab259341	1,000	rabbit
IL-Rα (human samples)	ThermoFisher	PA5-102425	1,000	rabbit

Table S4. Patient characteristics for pericardial fluid samples.

Characteristics of paired pericardial fluid samples	
poAF, n(%)	6 (60)
Female/Male	7/3
Age, y (mean±SD)	62.9 ± 1.6
CABG, n(%)	7 (70)
Aortic or valvular surgery, n(%)	3 (30)
Beta blocker (ppx), n(%)	1 (10)
Amiodarone (ppx), n(%)	1 (10)
Beta blockers (AMB), n(%)	8 (80)
Statins (AMB), n(%)	7 (70)
ASA (AMB), n(%)	6 (60)
DHP CCB (AMB), n(%)	3 (30)
ACEi/ARB (AMB), n(%)	6 (60)
Diuretic (AMB), n(%)	4 (40)
HTN, n(%)	8 (80)
HLD, n(%)	7 (70)
DM, n(%)	6 (60)
CAD, n(%)	6 (60)
MR, n(%)	3 (30)

Characteristics of SR vs. poAF patients				
	**SR**	**poAF**	***P* value**	**Test**
Patients, n	6	8	N/A	N/A
Female/Male	4/2	4/4	0.533	Fisher's
Age, y (mean±SD)	59.3 ± 11.12	65.9 ± 8.52	0.235	T-test
CABG, n(%)	4 (67)	6 (75)	>0.999	Fisher's
Aortic or valvular surgery, (%)	2 (33)	2 (25)	>0.999	Fisher's
Beta blocker (ppx), n(%)	1 (17)	2 (25)	>0.999	Fisher's
Amiodarone (ppx), n(%)	1 (17)	1 (13)	>0.999	Fisher's

Beta blockers (AMB), n(%)	6 (100)	5 (63)	0.209	Fisher's
Statins (AMB), n(%)	4 (67)	6 (75)	>0.999	Fisher's
ASA (AMB), n(%)	5 (83)	5 (63)	0.580	Fisher's
DHP CCB (AMB), n(%)	2 (33)	4 (50)	0.627	Fisher's
ACEi/ARB (AMB), n(%)	2 (33)	3 (38)	>0.999	Fisher's
Diuretic (AMB), n(%)	2 (33)	4 (50)	0.627	Fisher's
HTN, n(%)	5 (83)	6 (75)	>0.999	Fisher's
HLD, n(%)	5 (83)	7 (88)	>0.999	Fisher's
DM, n(%)	2 (33)	3 (38)	>0.999	Fisher's
CAD, n(%)	5 (83)	5 (63)	0.580	Fisher's
MR, n(%)	1 (17)	3 (38)	0.580	Fisher's
Cardioversion with IV metoprolol	N/A	3 (38)	N/A	N/A
Cardioversion with IV amiodarone	N/A	5 (62)	N/A	N/A
DC Cardioversion	N/A	0 (0)	N/A	N/A

Abbreviations: ACEi - angiotensin converting enzyme inhibitor, AMB - ambulatory, ARB - angiotenin receptor blocker, CABG - coronary artery bypass grafting, CAD - coronary artery disease, CCB - calcium channel blocker, DC - direct current, DHP - dihydropyridine, DM - diabetes mellitus, HLD - hyperlipidemia, HTN - hypertension, IV - intravenous, MR - mitral regurgitation, poAF - postoperative atrial fibrillation, ppx - prophylaxis, SD - standard deviation, SR - sinus rhythm

Table S5. Patient characteristics for IL-6 and IL-6Rα protein expression in human atrial lysates.

	SR	poAF	P-value	Test
Patients, n	11	9	N/A	N/A
Gender, M/F	8/3	6/3	>0.999	Fisher's exact test
Age, y	61.8 ± 9.3	71.1 ± 6.6	0.023	Mann Whitney test
Body mass index, kg/m^2	27.4 ± 2.3	27.6 ± 3.0	0.586	Mann Whitney test
CAD, n (%)	4 (36)	1 (11)	0.319	Fisher's exact test
AVD/MVD, n (%)	2 (18)	5 (56)	0.160	Fisher's exact test
CAD+AVD/MVD, n (%)	5 (45)	3 (33)	0.670	Fisher's exact test
Hypertension, n (%)	10 (91)	7 (78)	0.566	Fisher's exact test
Diabetes, n (%)	3 (27)	5 (56)	0.362	Fisher's exact test
Hyperlipidemia, n (%)	7 (64)	5 (56)	>0.999	Fisher's exact test
LVEF, %§	54.4 ± 13.8	56.0 ± 11.0	0.905	Mann Whitney test
LA, mm$^\#$	37.4 ± 7.8	40.2 ± 5.8	0.556	Mann Whitney test
Digitalis, n (%)	0 (0)	0 (0)	>0.999	Fisher's exact test
ACEi OR ARB, n (%)	8 (73)	7 (78)	>0.999	Fisher's exact test
β-Blockers, n (%)	6 (55)	5 (56)	>0.999	Fisher's exact test
Dihydropyridines, n (%)	2 (18)	2 (22)	>0.999	Fisher's exact test
Diuretics, n (%)	5 (45)	5 (56)	>0.999	Fisher's exact test
Nitrates, n (%)	1 (9)	0 (0)	>0.999	Fisher's exact test
Lipid-lowering drugs, n (%)	7 (64)	7 (78)	0.747	Fisher's exact test

Abbreviations: ACEi – angiotensin converting enzyme inhibitor, ARB – angiotensin II receptor blocker, AVD – aortic valve disease, CAD – coronary artery disease, dPCR – digital polymerase chain reaction, LA – left atrium, LVEF – left ventricular ejection fraction, MVD – mitral valve disease, N/A – not applicable, poAF – postoperative atrial fibrillation, SR – sinus rhythm

**P*<0.05 *versus* SR for categorical variables. ‡*P*<0.05 *versus* SR for continuous variables. §Data were not available for 1 SR and 2 poAF. $^\#$Data were not available for 6 SR, and 5 poAF. Continuous variables are presented as mean ± standard deviation.

Table S6. Patient characteristics gp130 protein expression in human atrial lysates.

	SR	poAF	P-value	Test
Patients, n	10	8	N/A	N/A
Gender, M/F	9/1	6/2	0.559	Fisher's exact test
Age, y	65.6 ± 8.2	73.8 ± 6.5	0.036	Mann Whitney test
Body mass index, kg/m^2	28.1 ± 3.5	27.6 ± 4.2	0.447	Mann Whitney test
CAD, n (%)	4 (40)	2 (25)	0.638	Fisher's exact test
AVD/MVD, n (%)	3 (30)	4 (50)	0.631	Fisher's exact test
CAD+AVD/MVD, n (%)	3 (30)	2 (25)	>0.999	Fisher's exact test
Hypertension, n (%)	9 (90)	6 (75)	0.559	Fisher's exact test
Diabetes, n (%)	2 (20)	5 (62)	0.145	Fisher's exact test
Hyperlipidemia, n (%)	5 (50)	5 (62)	0.664	Fisher's exact test
LVEF, %[§]	56.2 ± 7.1	58.7 ± 11.5	0.488	Mann Whitney test
LA, mm[#]	43.4 ± 10.1	39.3 ± 6.4	0.786	Mann Whitney test
Digitalis, n (%)	0 (0)	0 (0)	>0.999	Fisher's exact test
ACEi OR ARB, n (%)	8 (80)	6 (75)	>0.999	Fisher's exact test
β-Blockers, n (%)	5 (50)	4 (50)	>0.999	Fisher's exact test
Dihydropyridines, n (%)	3 (30)	2 (25)	>0.999	Fisher's exact test
Diuretics, n (%)	3 (30)	3 (38)	>0.999	Fisher's exact test
Nitrates, n (%)	0 (0)	0 (0)	>0.999	Fisher's exact test
Lipid-lowering drugs, n (%)	8 (80)	5 (62)	0.608	Fisher's exact test

Abbreviations: ACEi – angiotensin converting enzyme inhibitor, ARB – angiotensin II receptor blocker, AVD – aortic valve disease, CAD – coronary artery disease, dPCR – digital polymerase chain reaction, LA – left atrium, LVEF – left ventricular ejection fraction, MVD – mitral valve disease, N/A – not applicable, poAF – postoperative atrial fibrillation, SR – sinus rhythm

*$P<0.05$ *versus* SR for categorical variables. [‡]$P<0.05$ *versus* SR for continuous variables. [§]Data were not available for 2 SR and 2 poAF. [#]Data were not available for 5 SR, and 5 poAF. Continuous variables are presented as mean ± standard deviation.

Table S7. Patient characteristics for dPCR of human atrial lysates.

	SR	poAF	P-value	Test
Patients, n	8	8	N/A	N/A
Gender, M/F	6/2	5/3	>0.999	Fisher's exact test
Age, y	64.6 ± 7.6	72.6 ± 11.6	0.122	Mann Whitney test
Body mass index, kg/m^2	25.0 ± 5.0	29.1 ± 7.9	0.239	Mann Whitney test
CAD, n (%)	6 (75)	6 (75)	>0.999	Fisher's exact test
AVD/MVD, n (%)	2 (25)	1 (12)	>0.999	Fisher's exact test
CAD+AVD/MVD, n (%)	0 (0)	1 (12)	>0.999	Fisher's exact test
Hypertension, n (%)	3 (38)	7 (88)	0.119	Fisher's exact test
Diabetes, n (%)	0 (0)	2 (25)	0.467	Fisher's exact test
Hyperlipidemia, n (%)	2 (25)	3 (38)	>0.999	Fisher's exact test
LVEF, %[§]	48.7 ± 16.4	49.0 ± 12.2	0.915	Mann Whitney test
LA, mm[#]	39.0 ± 10.3	34.5 ± 0.71	0.585	Mann Whitney test
Digitalis, n (%)	0 (0)	0 (0)	>0.999	Fisher's exact test
ACEi OR ARB, n (%)	6 (75)	6 (75)	>0.999	Fisher's exact test
β-Blockers, n (%)	2 (25)	5 (62)	0.315	Fisher's exact test
Dihydropyridines, n (%)	1 (12)	3 (38)	0.569	Fisher's exact test
Diuretics, n (%)	4 (50)	4 (50)	>0.999	Fisher's exact test
Nitrates, n (%)	0 (0)	1 (12)	>0.999	Fisher's exact test
Lipid-lowering drugs, n (%)	4 (50)	3 (38)	>0.999	Fisher's exact test

Abbreviations: ACEi – angiotensin converting enzyme inhibitor, ARB – angiotensin II receptor blocker, AVD – aortic valve disease, CAD – coronary artery disease, dPCR – digital polymerase chain reaction, LA – left atrium, LVEF – left ventricular ejection fraction, MVD – mitral valve disease, N/A – not applicable, poAF – postoperative atrial fibrillation, SR – sinus rhythm

*$P<0.05$ *versus* SR for categorical variables. ‡$P<0.05$ *versus* SR for continuous variables. [§]Data were not available for 2 SR and 1 poAF. [#]Data were not available for 3 SR, and 6 poAF. Continuous variables are presented as mean ± standard deviation.

Table S8. Mouse PES parameters for cardiomyocyte-specific *Stat3* cKO studies.

	WT (N=13)		cKO (N=10)		*P*
	Mean ± SEM	IQR (range)	Mean ± SEM	IQR (range)	
RR	124.2 ± 3.61	15 (47)	122.0 ± 3.57	17 (39)	0.679
PR	44.6 0.80	5.3 (8.7)	47.3 ± 1.26	5.0 (14)	0.078
QRS	13.1 ± 0.24	1.4 (2.6)	13.8 ± 0.35	1.4 (4.0)	0.119
SNRT	187.1 ± 13.0	94 (122)	177.8 ± 12.5	39 (129)	0.621
AVERP	53.4 ± 1.02	7.0 (12)	53.3 ± 1.26	5.0 (12)	0.976

Electrocardiographic parameters from mouse PES studies. Values displayed in ms as mean (SEM). *P* values were obtained from two-sample t-test. *Abbreviations*: AVERP - atrioventricular effective refractory period, cKO - conditional knockout, IQR - interquartile range, SEM - standard error of the mean, SNRT - sinus node recovery time, Thor - thoracotomy, WT - wild-type.

Table S9. Novelty of current study compared to prior studies.

Prior finding	Ref	Novelty of current study
IL-6 inhibition ↓ poAF in animal models	(19-21)	IL-6 was pharmacologically (globally) inhibited in these studies. Our study is the first to show that macrophage depletion and conditional knockout of IL-6 receptors in macrophages alone were sufficient to protect against poAF, indicating that IL-6 trans-signaling driven by myeloid cells is the critical aspect of IL-6 signaling in poAF.
STAT3 inhibition ↓ poAF in animal models through ↓ fibrosis	(19)	We did not observe histologic atrial fibrosis in our poAF mouse model, which is not surprising given the short postoperative period of 72-h. Indeed, conditional *Stat3* knockout in cardiomyocytes was sufficient to prevent poAF, indicating that STAT3 activation in cardiomyocytes is necessary for poAF. We have provided further translational findings by demonstrating that FDA-orphan designated phospho-STAT3 inhibitor TTI-101 reduced poAF incidence in mice.
IL-6 trans-signaling inhibition with soluble gp130 ↓ chronic AF in mice	(22)	A prior study of chronic, not postop, AF demonstrated that IL-6 trans-signaling led to AF via fibrosis, although no ACM Ca^{2+} imaging studies were conducted. We did not observe atrial fibrosis in our poAF mouse model but rather a robust arrhythmogenic STAT3-driven Ca^{2+} mishandling phenotype in both mouse and human ACMs. Moreover, we build upon this study by demonstrating that inhibition of the myeloid contribution (as opposed to global pharmacologic inhibition) to IL-6 trans-signaling is protective against poAF.
IL-6 ↑ Ca^{2+} alternans in isolated rat hearts	(20)	Study utilized optical mapping in isolated whole rat hearts, not individual ACMs, and does not provide a mechanistic pathway driving arrhythmogenesis. Our study mechanistically demonstrates in individual mouse and human ACMs that IL-6 induces an arrhythmogenic response characterized by CaMKII-dependent RyR2 Ca^{2+} sparks.
IL-6 ↓ CaT amplitude and SR Ca^{2+} load in iPSC-CMs	(23)	A reduction in CaT amplitude and SR Ca^{2+} load suggest diastolic SR Ca^{2+} leak, which most directly relates to arrhythmogenic potential, although this was not directly shown in this study. We show that IL-6 increased RyR2-dependent pro-arrhythmogenic Ca^{2+} sparks. Moreover, we used primary cells isolated from mouse atria, which better recapitulates *in vivo* pathophysiology compared to cell lines.

ROS-mediated CaMKII activation ↑ late Na^+ current in PV CMs	(24)	While augmentation of late Na^+ current may play a role in poAF, our study is the first to show that CaMKII-mediated phosphorylation of RyR2 at S2814 is a necessary action of CaMKII in poAF development as non-phosphorylatable $RyR2^{S2814A}$ mice were protected from poAF.
ACMs from poAF patients had ↓ CaT amplitude, ↑ SR Ca^{2+} leak compared to ACMs from sinus rhythm patients	(25)	Study reveals possible presence of pre-existing pro-arrhythmogenic substrate. Our study focuses on the role of IL-6-mediated pro-arrhythmogenic changes occurring *after* surgery in poAF. Indeed, we collected pericardial fluid during the perioperative period to show that macrophages infiltrate human hearts after cardiac surgery and that this infiltration is greater in poAF versus sinus rhythm patients.

Abbreviations: CaMKII – Ca^{2+}/calmodulin-dependent protein kinase II, CaT – Ca^{2+} transient, CM – cardiomyocyte, PV – pulmonary vein, ROS – reactive oxygen species, RyR2 – ryanodine receptor-2, SR – sarcoplasmic reticulum, TAC – thoracic aortic constriction

Figure S1. Representative ECG traces. (A) Studies of poAF in mice were performed as previously described (1). Briefly, mice underwent thoracotomy followed by PES on postoperative day three to assess poAF inducibility. **(B)** Representative surface and atrial electrogram traces showing the end of atrial burst pacing followed by sinus rhythm and poAF in sham and thoracotomy mice. **(C)** Surface ECG tracing showing the full length of the poAF episode in the same thoracotomy mouse used in (B). Red arrow denotes the spontaneous conversion to sinus rhythm. *Abbreviations*: Px - pericardiectomy, PES - programmed electrical stimulation, Thor - thoracotomy, AF - atrial fibrillation, SR - sinus rhythm.

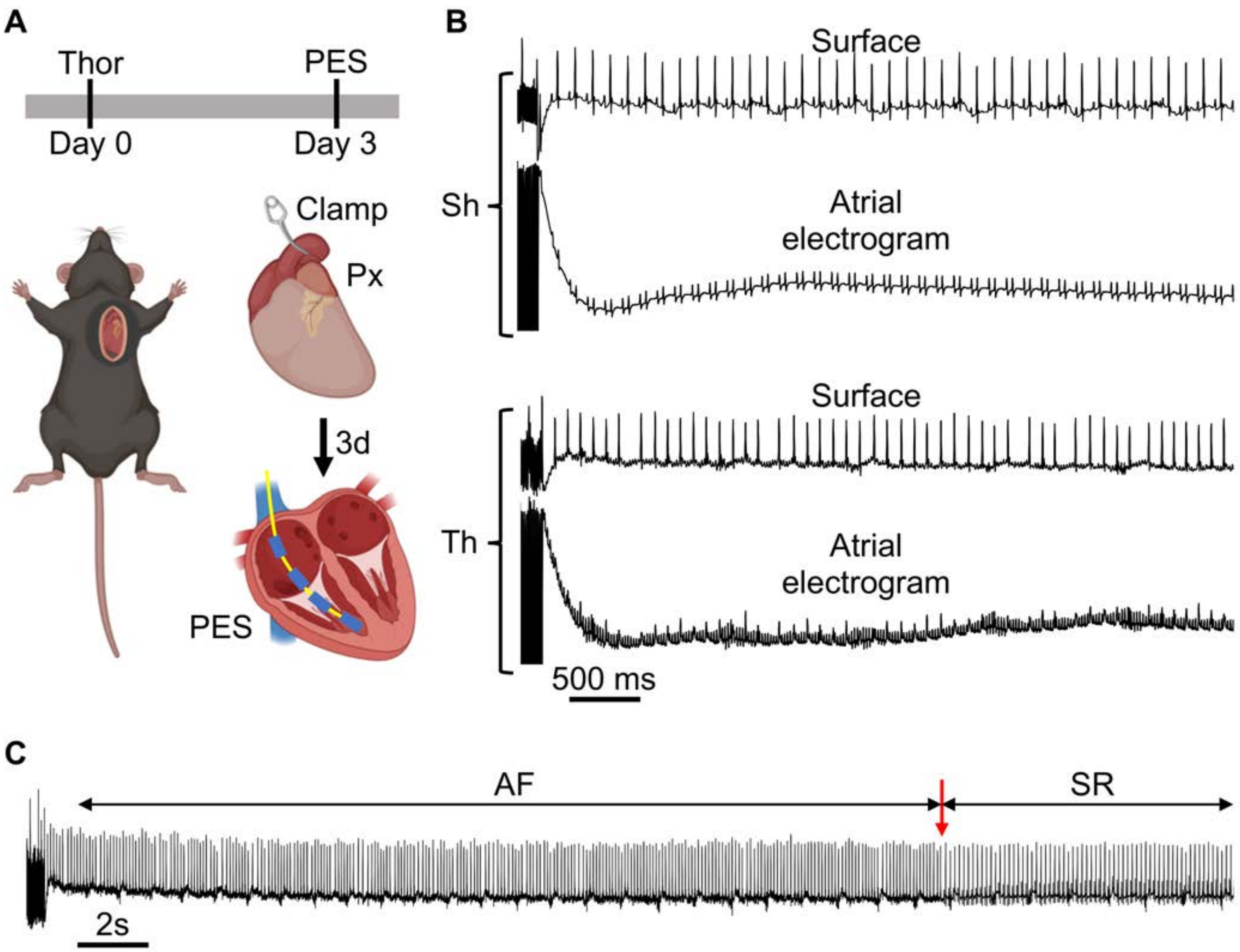

Figure S2. Mouse scRNAseq cell clustering. Atrial non-myocytes from sham (Sh) (n=3) and TAF (n=3) mice were analyzed by scRNAseq. (**A**) Cells were clustered by UMAP dimensional reduction and (**B**) labeled according to common cell markers, which revealed the presence of 9 cell types (FB, Mac, EC, B, PMN, T, Mural, Neuro, and Plasma). (**C**) Macrophage subclusters were labeled by common markers for anti-inflammatory, mixed, pro-inflammatory/infiltrating macrophages, proliferating, and DCs, demonstrating the presence of 4 macrophage subclusters and 1 DC cluster. *Abbreviations*: Anti - anti-inflammatory, B - B cell, DC - dendritic cell, EC - endothelial cell, FB - fibroblast, Macs - macrophages, PMN - polymorphonuclear neutrophil, Pro/infil - pro-inflammatory/infiltrating, Prolif - proliferating, SMC - smooth muscle cell, T - T cell, TAF - thoracotomy atrial fibrillation.

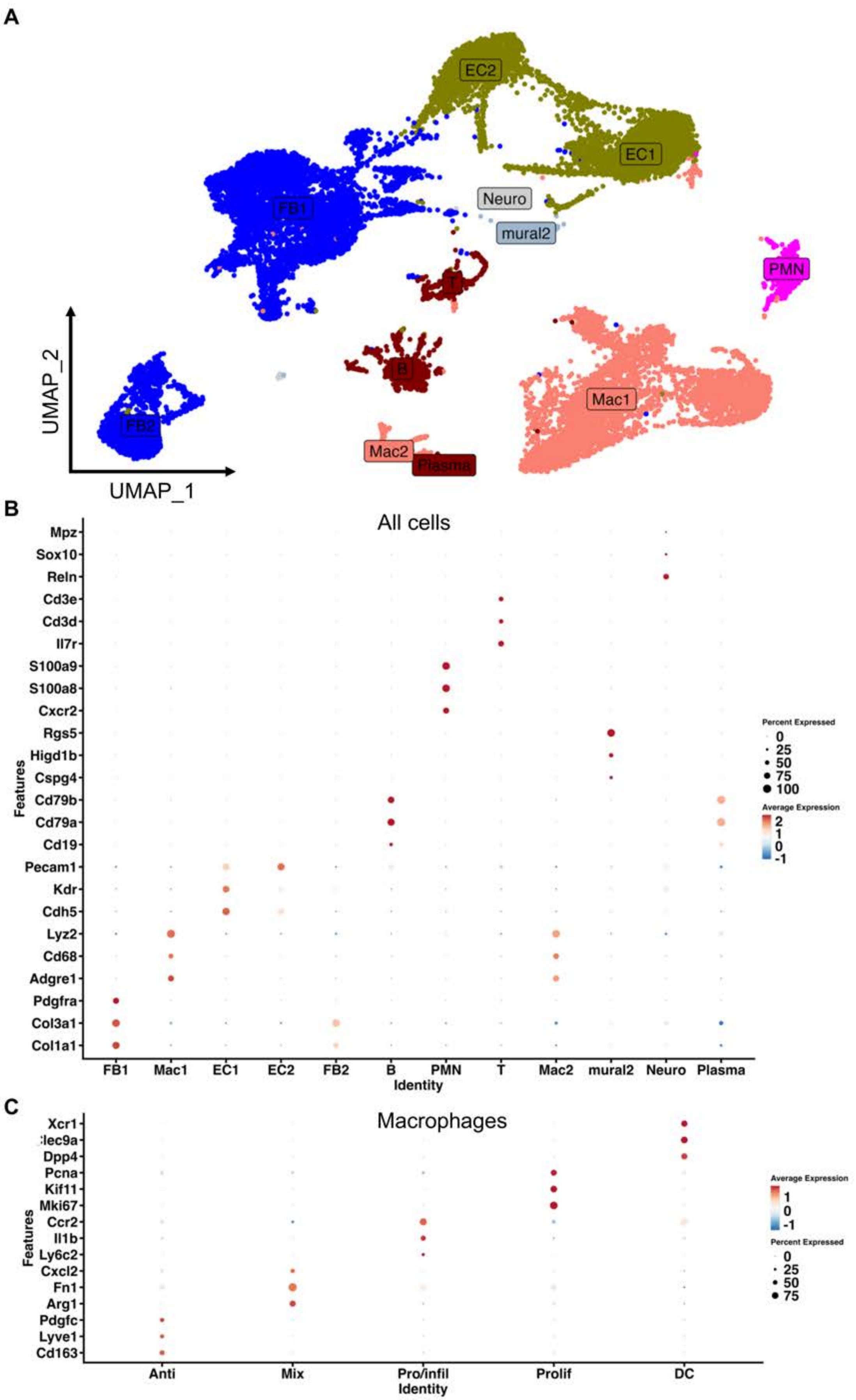
A
EC2
EC1
FB1
Neuro
mural2
PMN
T
B
Mac1
FB2
Mac2
Plasma
UMAP_2
UMAP_1
B
All cells
Mpz
Sox10
Reln
Cd3e
Cd3d
Il7r
S100a9
S100a8
Cxcr2
Rgs5
Higd1b
Cspg4
Cd79b
Cd79a
Cd19
Pecam1
Kdr
Cdh5
Lyz2
Cd68
Adgre1
Pdgfra
Col3a1
Col1a1
Features
FB1 Mac1 EC1 EC2 FB2 B PMN T Mac2 mural2 Neuro Plasma
Identity
Percent Expressed
0
25
50
75
100
Average Expression
2
1
0
-1
C
Macrophages
Xcr1
Dpp4
Pcna
Kif11
Mki67
Ccr2
Il1b
Ly6c2
Cxcl2
Fn1
Arg1
Pdgfc
Lyve1
Cd163
Features
Anti Mix Pro/infil Prolif DC
Identity
Average Expression
1
0
-1
Percent Expressed
0
25
50
75

1

Figure S3. Immunohistochemical staining of macrophages in the atria. (**A**) Immunohistochemistry for F4/80$^+$ macrophages using DAB staining on paraffin-embedded four-chamber heart sections harvested from sham (Sh; n=4) and thoracotomy atrial fibrillation (TAF; n=4) mice on postoperative day three. Quantification of the number of F4/80$^+$ cells per mm^2 in (**B**) both atria, (**C**) right atria, and (**D**) left atria. (**E**) Quantification of ventricular F4/80$^+$ macrophages. Red arrows indicate macrophages. Please note that these data show that F4/80$^+$ macrophages infiltrate the atria, not ventricles, after thoracotomy. *P*-values in (B-E) were obtained from two-sample t-tests. *Abbreviations*: Sh - sham, TAF - thoracotomy atrial fibrillation.

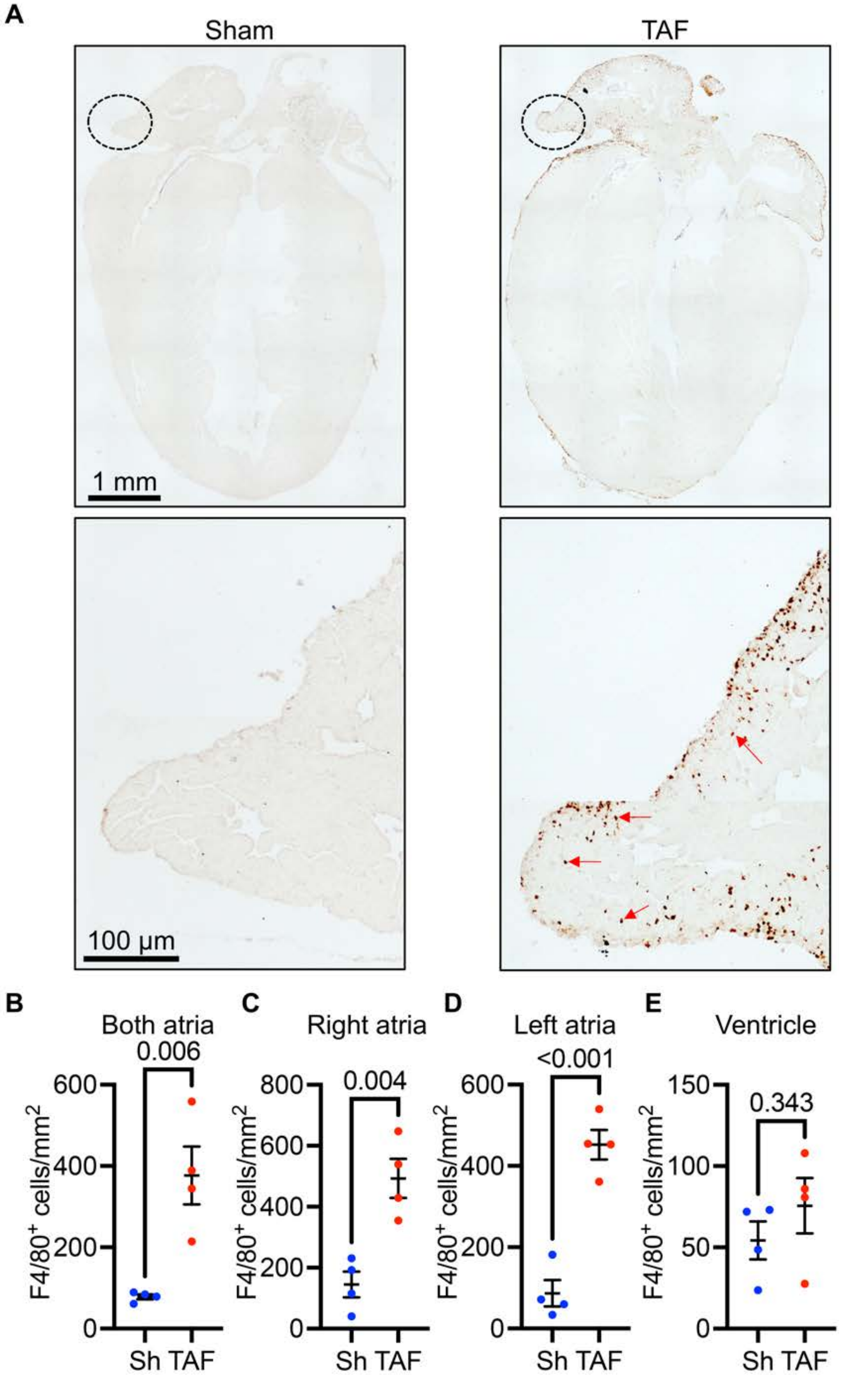
A
Sham
TAF
1 mm
100 µm
B
Both atria
0.006
F4/80+ cells/mm2
600
400
200
0
Sh TAF
C
Right atria
0.004
800
600
400
200
0
F4/80+ cells/mm2
Sh TAF
D
Left atria
<0.001
600
400
200
0
F4/80+ cells/mm2
Sh TAF
E
Ventricle
0.343
150
100
50
0
F4/80+ cells/mm2
Sh TAF

0

Figure S4. Ventricular arrhythmias are unaltered in mice after thoracotomy. (A) VT incidence, assessed by ventricular burst pacing at cycle thresholds of 60 ms and 40 ms (see supplementary methods), in sham and thoracotomy mice. **(B)** Ventricular *Il6* was increased while **(C)** *Il6ra* was unchanged in TAF versus Sh mice. **(D)** Similar findings were observed at the IL-6 and IL-6Rα protein levels. Please note that the greater ventricular IL-6 is consistent with the increase in circulating (i.e., systemic IL-6) while unchanged IL-6Rα expression is consistent with the unaltered ventricular macrophage infiltration and VT incidence. *P* value in (A) was derived from Fisher's exact test. *P* values in (B-D) were derived from two-sample t-test. Each dot in B-D represents one mouse. *Abbreviations*: Sh - sham, TAF - thoracotomy atrial fibrillation, VT - ventricular tachycardia.

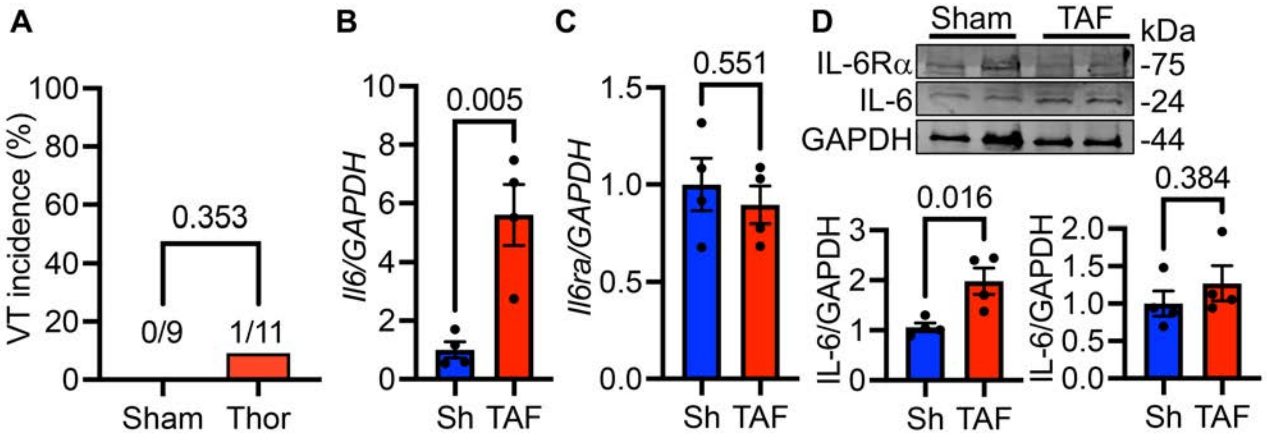

Figure S5. Atrial fibrosis does not play a role in poAF. (**A**) Whole hearts from sham (n=5) and TAF (n=5) mice were sectioned in 4-chamber view and stained for fibrosis with picrosirius red. (**B**) Quantification of atrial fibrosis averaged over both atria and individually in right and left atria. Please note that these data show that significant atrial fibrosis does not occur in our poAF mouse model over a postoperative period of 72-h. *P*-values in (B) was obtained from two-sample t-test.

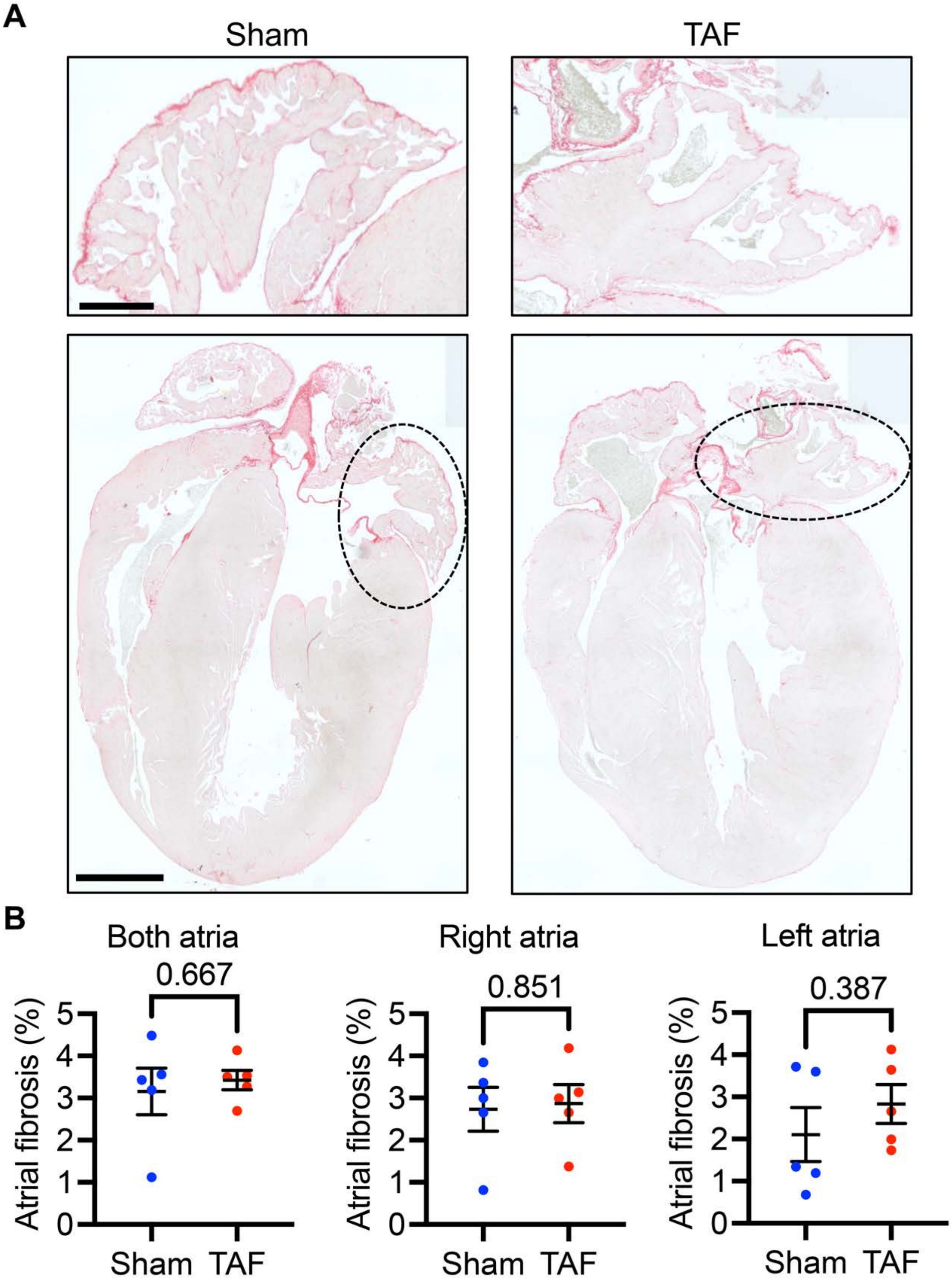
A
Sham
TAF
B
Both atria
0.667
Right atria
0.851
Left atria
0.387
Atrial fibrosis (%)
Sham
TAF

6

Figure S6. CCR2^{+} monocytes infiltrate the atria after cardiac surgery. CellChat was conducted using our scRNAseq dataset to compare differential cell-cell interactions in sham vs TAF mice. (**A**) The total number and weight of cell-cell interactions was increased in TAF mice, (**B**) primarily driven by an increase in macrophage-mediated signaling. (**C**) Indeed, macrophage cluster 1 was the cell population with the greatest number of differential signaling pathways between sham and TAF, and (**D**) the CCL pathway driven by (**E**) CCR2 was one of the top pathways upregulated in these macrophages. (**F-G**) These findings were confirmed by flow cytometry showing a significant increase in CCR2^{+} monocytes in the atria of TAF versus sham mice. Please note that these data TAF mice have a significant increase in intercellular signaling primarily driven by macrophages, and that CCR2 is a likely receptor mediating atrial macrophage infiltration. Scale in (B) indicates normalized relative strength of each interaction pathway. *Abbreviations*: FB - fibroblast, Mac - macrophage, MIF - macrophage migration inhibitory factor, Mur - mural, Neu - neuron, PMN - polymorphonuclear neutrophil, Sh - sham, SPP1 - secreted phosphoprotein 1, TAF - thoracotomy atrial fibrillation.

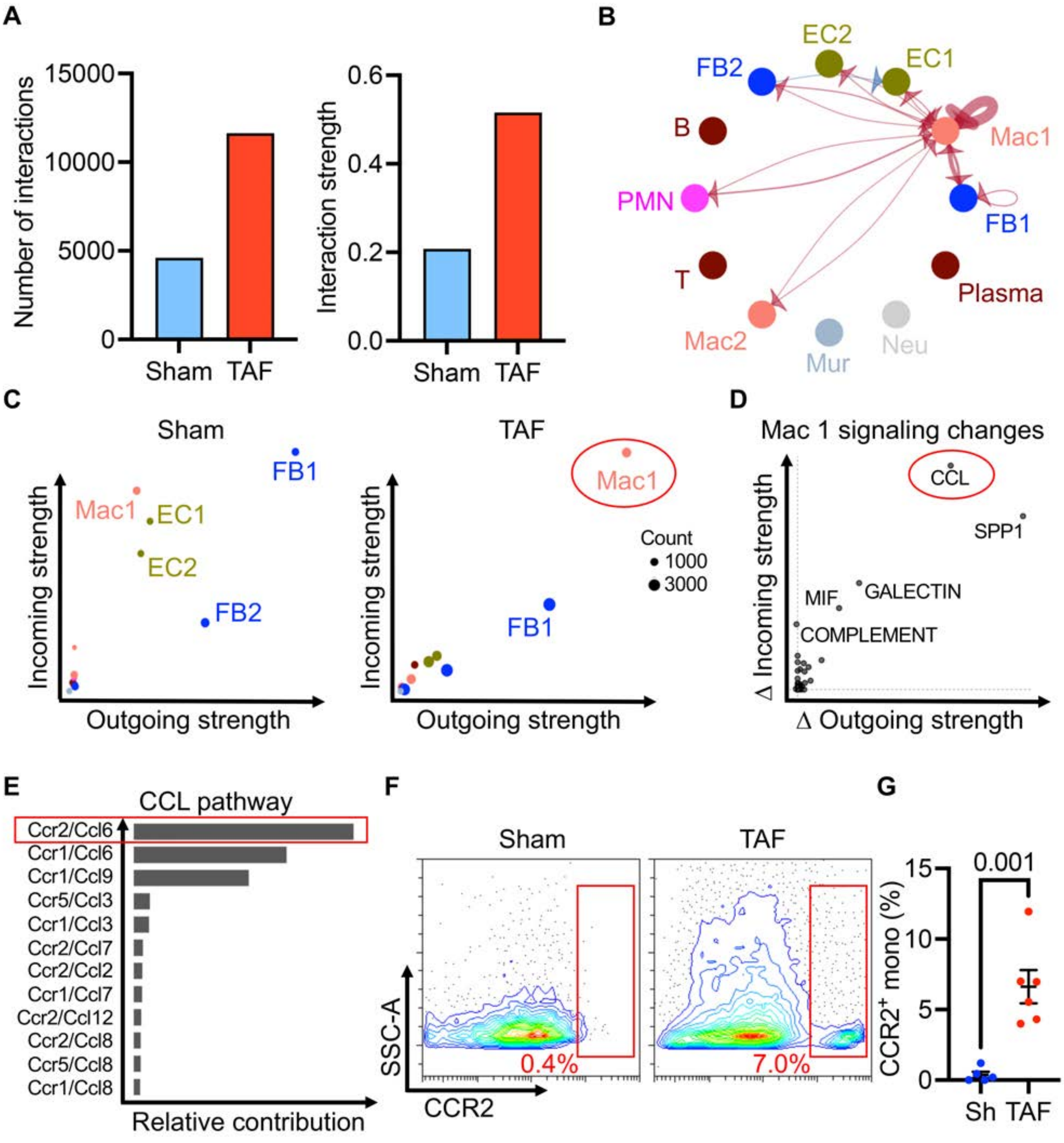
A
Number of interactions
15000
10000
5000
0
Sham
TAF
Interaction strength
0.6
0.4
0.2
0.0
B
EC2
EC1
FB2
B
Mac1
PMN
FB1
T
Plasma
Mac2
Mur
Neu
C
Sham
TAF
FB1
Mac1
EC1
EC2
FB2
Incoming strength
Outgoing strength
Count
1000
3000
D
Mac 1 signaling changes
CCL
SPP1
GALECTIN
MIF
COMPLEMENT
Δ Incoming strength
Δ Outgoing strength
E
CCL pathway
Ccr2/Ccl6
Ccr1/Ccl6
Ccr1/Ccl9
Ccr5/Ccl3
Ccr1/Ccl3
Ccr2/Ccl7
Ccr2/Ccl2
Ccr1/Ccl7
Ccr2/Ccl12
Ccr2/Ccl8
Ccr5/Ccl8
Ccr1/Ccl8
Relative contribution
F
Sham
TAF
SSC-A
CCR2
0.4%
7.0%
G
0.001
CCR2+ mono (%)
15
10
5
0
Sh
TAF

Figure S7. Clodronate liposome macrophage depletion. (**A**) CL was injected one hour prior to cardiac surgery, followed by programmed electrical stimulation (PES) studies on postoperative day three. (**B**) Validation of CL macrophage depletion three days after injection by flow cytometry showing decreased F4/80+ splenic macrophages. Please note that these data show that CL prevents the infiltration of macrophages into the atria. *P*-value in (B) was obtained from two-sample t-test. *Abbreviations*: CL - clodronate liposome, TAF - thoracotomy atrial fibrillation, Veh - vehicle.

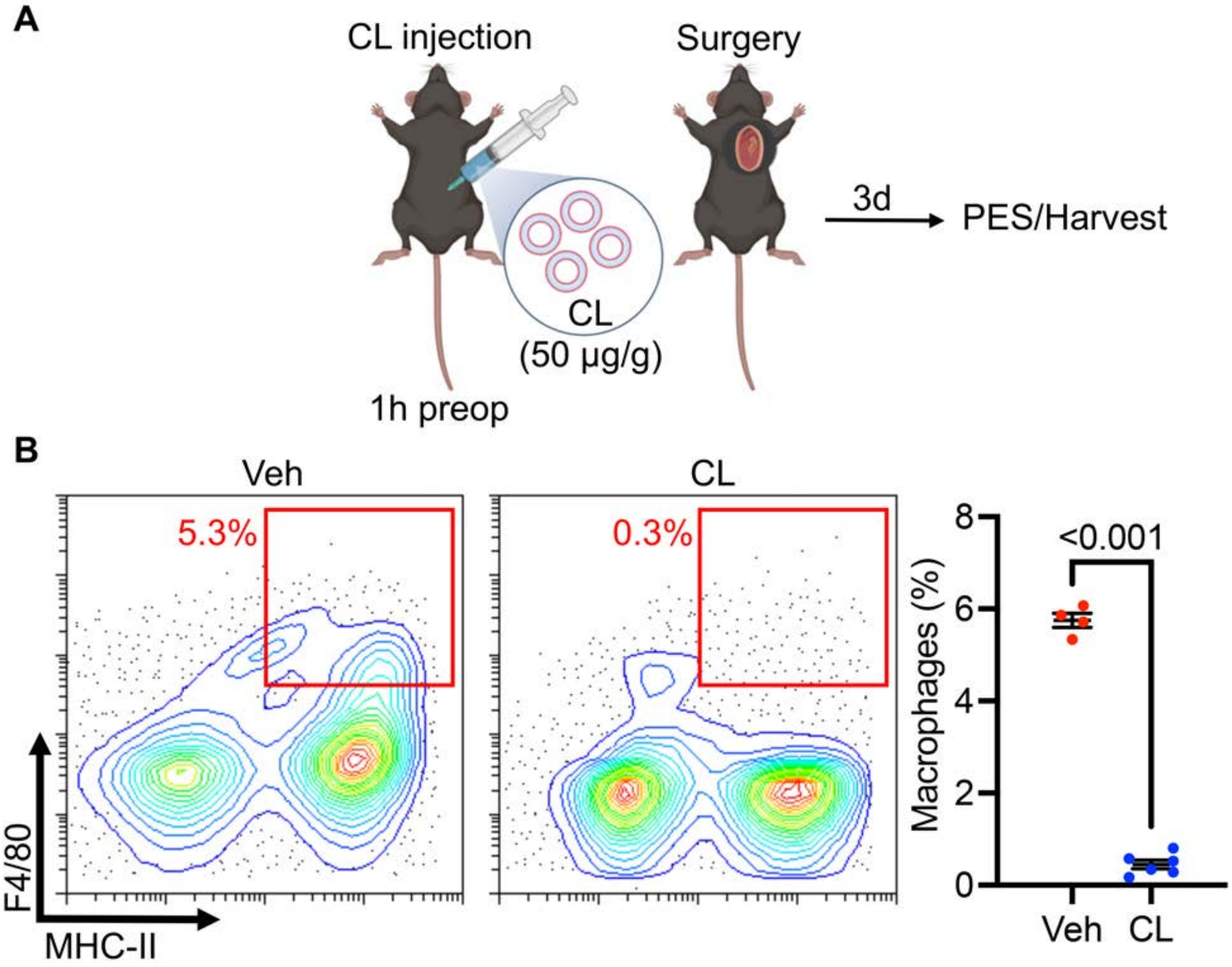

Figure S8. Baseline ECG parameters in mouse cohorts. Baseline RR interval, PR interval, QRS duration, SNRT, and AVERP all mouse cohorts listed in Table 1 including (**A**) CL, (**B**) *Il6ra* cKO, (**C**) STAT3 inhibition, and (**D**) RyR2-S2814 phosphomutants. SNRT was unable to be obtained for n=1 Sh, n=1 Th, n=1 S3I in (C) and n=2 Sh in (D). AVERP was unable to be obtained for n=2 CL in (A), n=1 Sh, n=2 Th in (C), and n=1 Sh, n=1 Th, n=2 S14A, n=2 S14D in (D). All *P* values were derived from one-way ANOVA followed by Tukey's post-hoc test at alpha=0.05. Each dot represents one mouse. *Abbreviations*: AVERP - atrioventricular effective refractory period, cKO - conditional knockout, CL - clodronate liposome, Sh - sham, SNRT - sinus node recovery time, Th - thoracotomy.

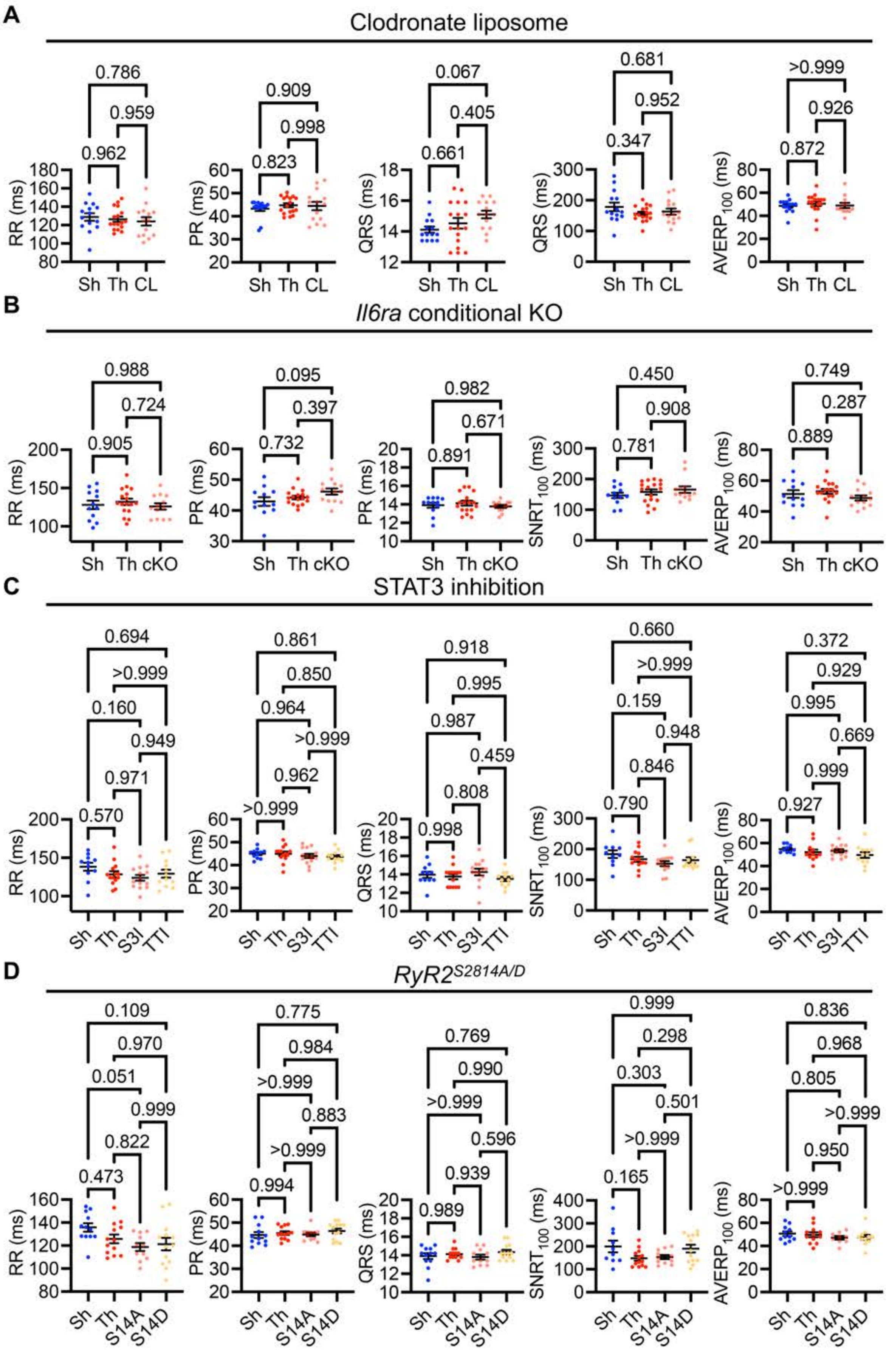
A
Clodronate liposome
0.786
0.959
0.962
RR (ms)
Sh Th CL
0.909
0.998
0.823
PR (ms)
0.067
0.405
0.661
QRS (ms)
0.681
0.952
0.347
QRS (ms)
>0.999
0.926
0.872
AVERP100 (ms)
B
Il6ra conditional KO
0.988
0.724
0.905
RR (ms)
Sh Th cKO
0.095
0.397
0.732
PR (ms)
0.982
0.671
0.891
PR (ms)
0.450
0.908
0.781
SNRT100 (ms)
0.749
0.287
0.889
AVERP100 (ms)
C
STAT3 inhibition
0.694
>0.999
0.160
0.949
0.971
0.570
RR (ms)
Sh Th S3I TTI
0.861
0.850
0.964
>0.999
0.962
>0.999
PR (ms)
0.918
0.995
0.987
0.459
0.808
0.998
QRS (ms)
0.660
>0.999
0.159
0.948
0.846
0.790
SNRT100 (ms)
0.372
0.929
0.995
0.669
0.999
0.927
AVERP100 (ms)
D
RyR2S2814A/D
0.109
0.970
0.051
0.999
0.822
0.473
RR (ms)
Sh Th S14A S14D
0.775
0.984
>0.999
0.883
>0.999
0.994
PR (ms)
0.769
0.990
>0.999
0.596
0.939
0.989
QRS (ms)
0.999
0.298
0.303
0.501
>0.999
0.165
SNRT100 (ms)
0.836
0.968
0.805
>0.999
0.950
>0.999
AVERP100 (ms)

Figure S9. Western blot and qPCR analysis of mouse atrial ADAM10 and ADAM17. Whole atria were harvested from Sh, TSR, and TAF mice (n=6 per group) on postoperative day three for RT-qPCR and western blot for (**A-B**) ADAM10 and (**C-D**) ADAM17. *P*-values in all panels were obtained from Tukey's post-hoc test after one-way ANOVA. Please note that these data show that ADAM10 and ADAM17, which are the major sheddases of IL-6Rα, are elevated in the atria of TAF versus TSR and Sh mice, indicating active IL-6 trans-signaling. *Abbreviations*: ANOVA - analysis of variance, poAF - postoperative atrial fibrillation, RT-qPCR - reverse transcription quantitative polymerase chain reaction, Sh - sham, TAF - thoracotomy atrial fibrillation, TSR - thoracotomy sinus rhythm.

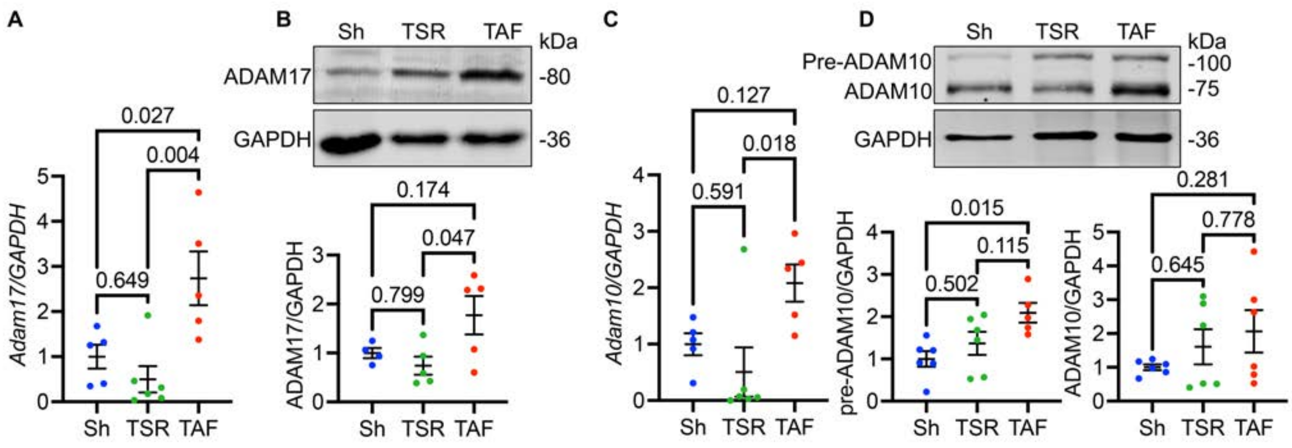

Figure S10. Western blotting and dPCR of human atrial appendages. Western blotting and dPCR were conducted on right atrial appendages collected at the time of surgery from patients that remained in sinus rhythm and those that developed postoperative atrial fibrillation with corresponding age-protein level correlations for (**A-C**) IL-6, (**D-F**) IL-6Rα, and (**G-I**) gp130. Please note that the same Revert700 blot is shown as a loading control in panels B and E. Please note that these data show that the IL-6 signaling axis demonstrates a weak elevation at the time of surgery, particularly for IL-6Rα which exhibits no change between SR and poAF patients at the time of surgery. *P*-values in (A), (B), (D), (E), (G), (H) were obtained from two-sample t-tests. *P* values in panels (C), (F), and (I) were obtained from Pearson's correlation. *Abbreviations*: dPCR - digital polymerase chain reaction, IL-6 - interleukin-6, IL-6Rα - interleukin-6 receptor alpha, poAF - postoperative atrial fibrillation, SR - sinus rhythm.

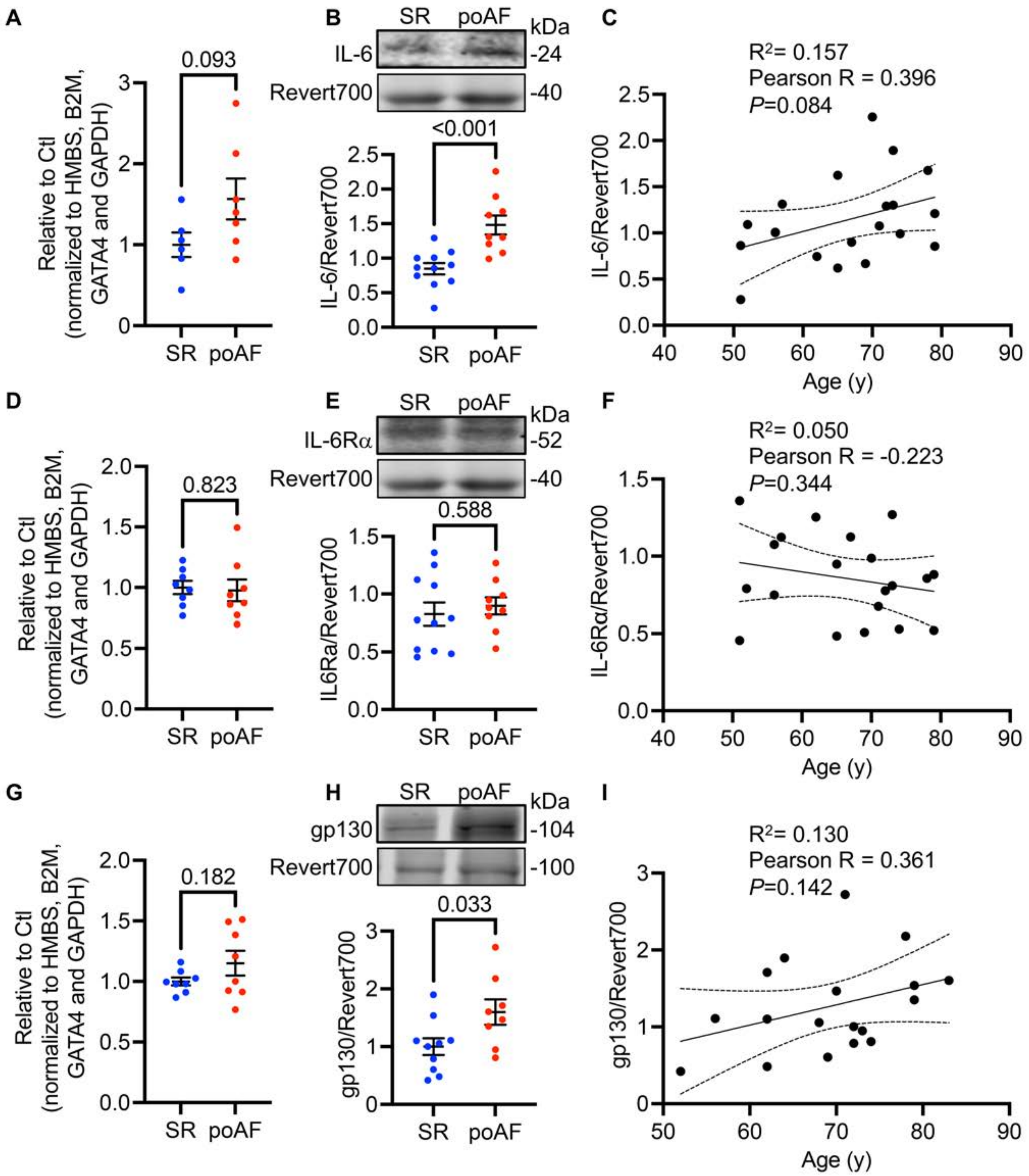
A
0.093
Relative to Ctl (normalized to HMBS, B2M, GATA4 and GAPDH)
SR
poAF
B
SR
poAF
kDa
IL-6
-24
Revert700
-40
<0.001
IL-6/Revert700
C
R²= 0.157
Pearson R = 0.396
P=0.084
IL-6/Revert700
Age (y)
D
0.823
E
IL-6Rα
-52
0.588
IL6Ra/Revert700
F
R²= 0.050
Pearson R = -0.223
P=0.344
IL-6Rα/Revert700
G
0.182
H
gp130
-104
-100
0.033
gp130/Revert700
I
R²= 0.130
Pearson R = 0.361
P=0.142

6

Figure S11. Validation of STAT3 inhibition and conditional knockout. STAT3 inhibition by S3I-201 and TTI-101 given once a day for three days after cardiac surgery to mice was validated by western blotting of whole atrial tissue harvested on postoperative day three for STAT3-Y705 phosphorylation, indicating STAT3 activation. Both (**A**) S3I-201 and (**B**) TTI-101 blunted STAT3 phosphorylation, validating treatment efficacy. **(C)** Cardiomyocyte-specific *Stat3* cKO was sufficient to decrease pSTAT3 and tSTAT3 in whole atria three days after thoracotomy. Please note that these data show that S3I-201 and TTI-101 both effectively blunt STAT3 activation by Tyr705 phosphorylation after thoracotomy. Importantly, *Stat3* cKO in cardiomyocytes was sufficient to decrease whole atrial pSTAT3, indicating that cardiomyocytes comprise the major pool of activated STAT3 after open-heart surgery. *P*-values in (A-B) were obtained from one-way ANOVA followed by Tukey's post-hoc test. *P* values in (C) were from two-sample t-test. *Abbreviations*: ANOVA - analysis of variance, cKO - conditional knockout S3I - S3I-201, Sh - sham, TAF - thoracotomy atrial fibrillation, TTI - TTI-101.

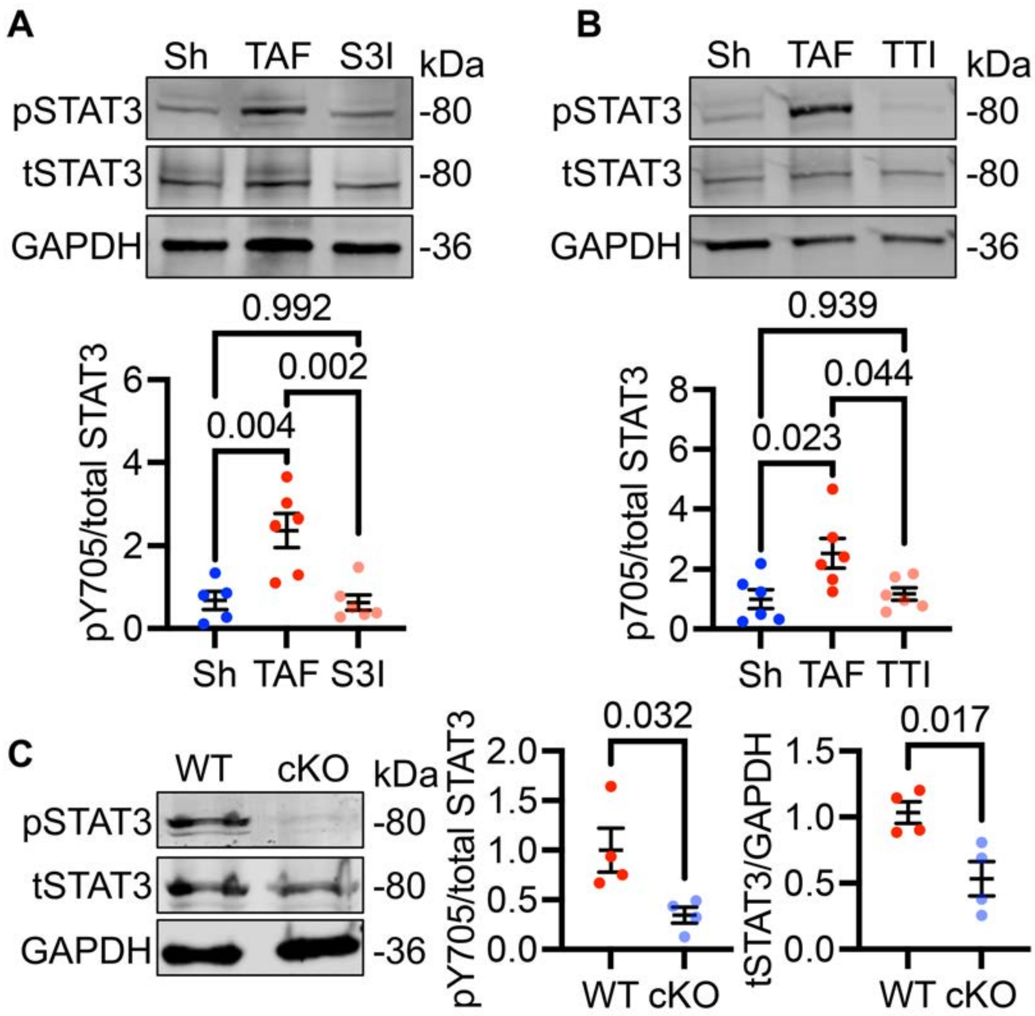
A
Sh
TAF
S3I
kDa
pSTAT3
-80
tSTAT3
-80
GAPDH
-36
0.992
0.002
0.004
pY705/total STAT3
6
4
2
0
Sh TAF S3I
B
Sh
TAF
TTI
kDa
pSTAT3
-80
tSTAT3
-80
GAPDH
-36
0.939
0.044
0.023
p705/total STAT3
8
6
4
2
0
Sh TAF TTI
C
WT
cKO
kDa
pSTAT3
-80
tSTAT3
-80
GAPDH
-36
0.032
pY705/total STAT3
2.0
1.5
1.0
0.5
0.0
WT cKO
0.017
tSTAT3/GAPDH
1.5
1.0
0.5
0.0
WT cKO

9

Figure S12. Ca^{2+} wave characteristics in IL-6/R-treated and TAF ACMs. The duration and latency of spontaneous Ca^{2+} waves, defined as >25% of the paced Ca^{2+} transient amplitude, were quantified in (**A-B**) IL-6/R versus vehicle-treated ACMs as well as (**C-D**) ACMs from Sh, TSR, TAF, and TAF+KN-93 mice. The duration of the longest Ca^{2+} wave for each ACM is plotted. Wave latency was defined as the time from the final field pacing stimulus to the first spontaneous Ca^{2+} wave. Please note that these data show that the duration of Ca^{2+} waves is greater in IL-6/R and TAF ACMs, indicative of greater arrhythmogenic potential. Each dot represents one ACM. Error bars denote mean +/- SEM. *P* values are derived from two-sample t-tests (A-B) or 1-way ANOVA followed by Tukey's at α=0.05 (C-D). *Abbreviations*: ACMs - atrial cardiomyocytes, Ca^{2+} - calcium ion, SEM - standard error of the mean, Sh - sham, TAF - thoracotomy atrial fibrillation, TSR - thoracotomy sinus rhythm, Veh - vehicle.

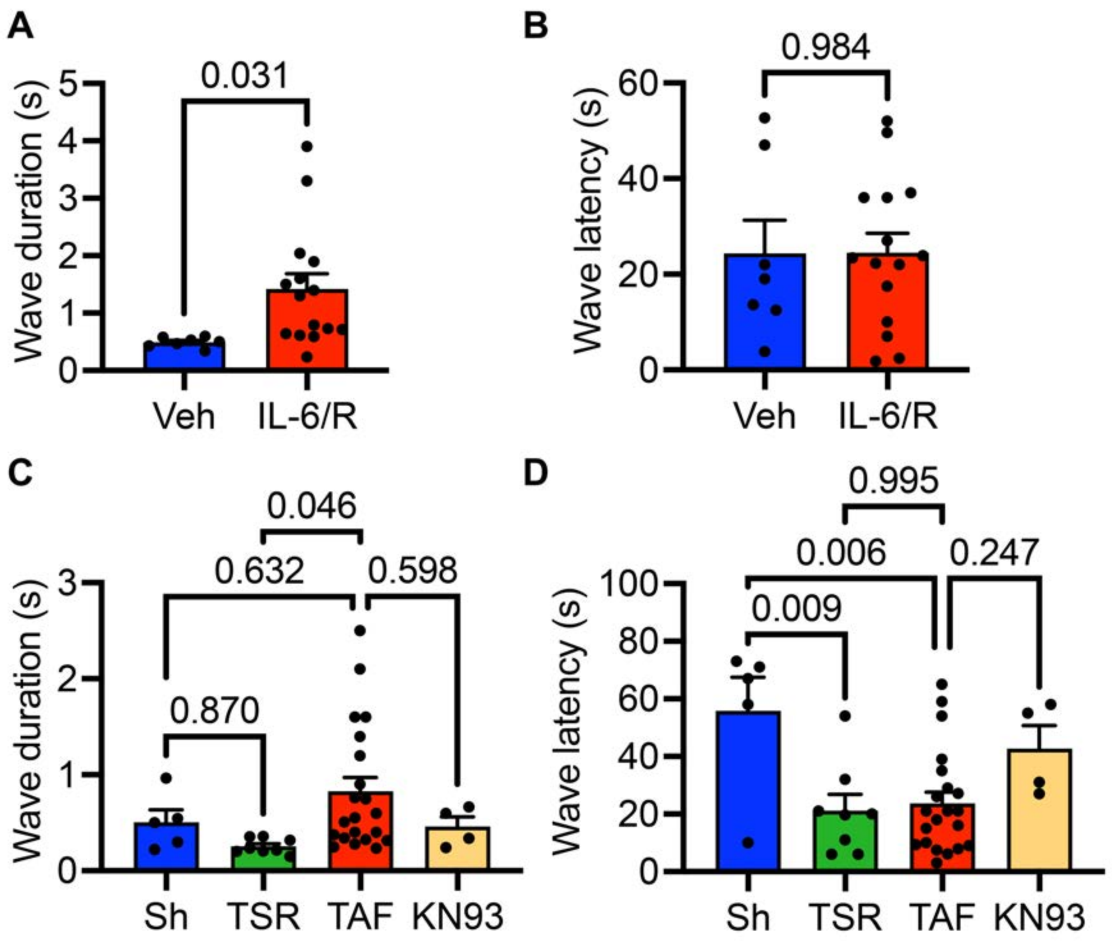

Figure S13. Paced Ca^{2+} transient amplitude and decay. (**A**) Representative paced CaT from Sh, TSR, TAF, and TAF treated with KN-93 ACMs. **(B)** Quantification of CaT amplitude and **(C)** CaT decay (1/Tau). Please note that these data show that CaT amplitude and decay did not differ among groups, suggesting that systolic function and SR Ca^{2+} reuptake, respectively, do not significantly contribute to poAF pathogenesis. *Abbreviations*: ACMs - atrial cardiomyocytes, CaT - calcium transient, Sh - sham, SR - sarcoplasmic reticulum, TAF - thoracotomy atrial fibrillation, TSR - thoracotomy sinus rhythm.

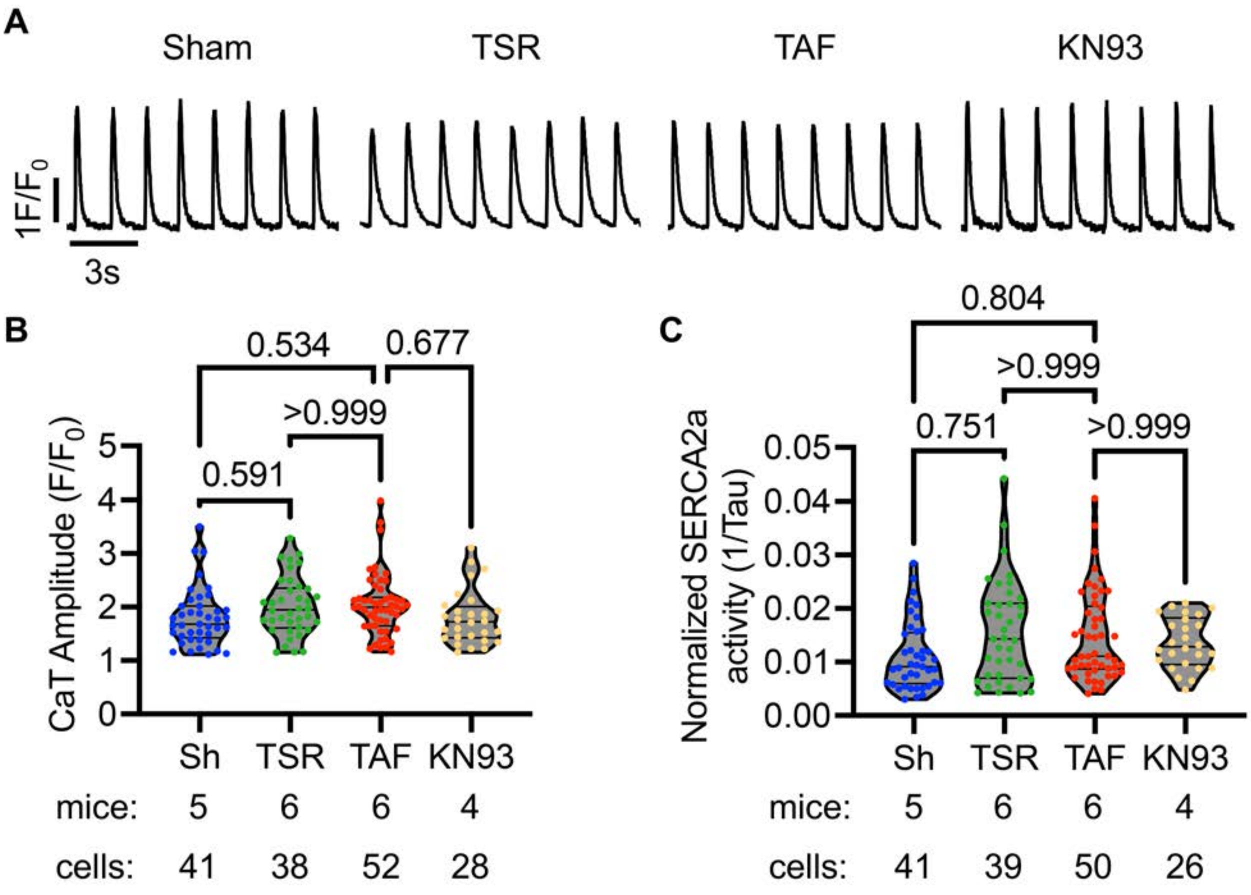

Figure S14. Effects of KN-92 and 2.5 versus 1µM KN-93 in TAF ACMs. ACMs from TAF mice were treated with vehicle versus 2.5µM KN-92 or 1 µM versus 2.5 µM KN-93, followed by assessment of **(A)** CaSpF and **(B)** SR Ca^{2+} load. Each dot represents on ACM. Please note that these data show that KN-93 attenuates arrhythmogenic Ca^{2+} sparks regardless of dose and KN-92 has no effect. *Abbreviations*: ACM - atrial cardiomyocyte, CaSpF - calcium spark frequency, SR - sarcoplasmic reticulum, TAF - thoracotomy atrial fibrillation, Veh - vehicle.

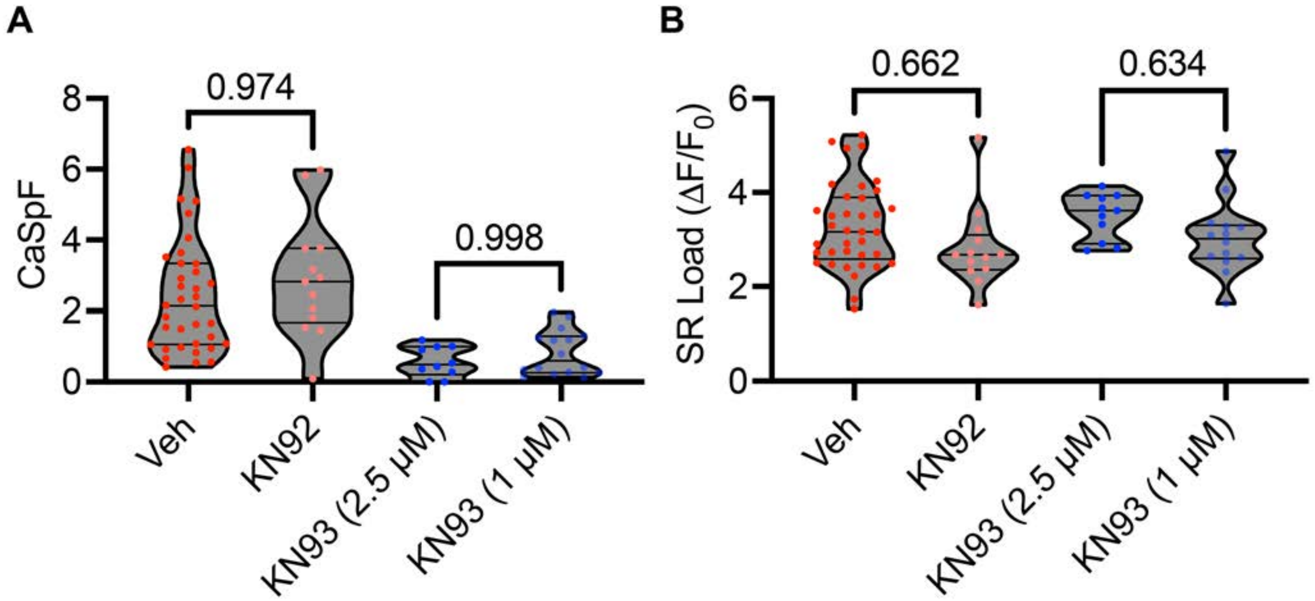

Figure S15. Ca^{2+} imaging in isolated ACMs paced at 2 Hz. Isolated ACMs from Sh and TAF mice were paced at 2 Hz by field stimulation, after which (**A**) Ca^{2+} transient amplitude and (**B**) CaSpF were quantified. ACMs paced at 1 Hz were the same ACMs used in Figures S14B for CaT amplitude and Figure 7B for CaSpF. Please note that these data show that Ca^{2+} transient amplitude did not differ in TAF versus Sham ACMs at 1 Hz and 2 Hz while CaSpF was consistently greater in TAF versus Sham ACMs at 1 Hz and 2 Hz, with an enhanced CaSpF in TAF ACMs paced at 2 Hz versus 1 Hz. *P* values in all panels were derived from one-way ANOVA followed by Tukey's post-hoc test at alpha=0.05. *Abbreviations*: CaSpF - calcium spark frequency, CaT - calcium transient, Sh - sham, TAF - thoracotomy atrial fibrillation.

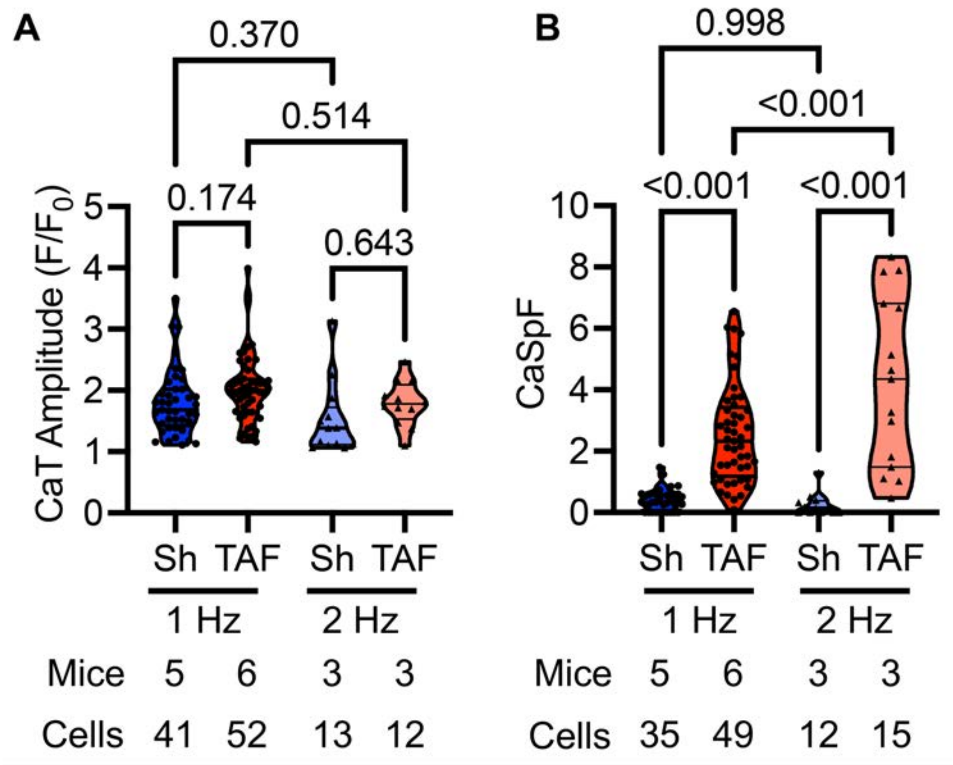

Figure S16. IL-6 levels were unchanged in RyR2-S2814 phospho-mutant mice. (A-B) Western blotting from whole atria in WT, *RyR2*^{S2814A}, and *RyR2*^{S2814D} homozygous mice three days after thoracotomy revealed no differences in IL-6 protein levels. *P* values were derived from one-way ANOVA followed by Tukey's post-hoc test at alpha=0.05. *Abbreviations*: S14A - *RyR2*^{S2814A}, S14D - *RyR2*^{S2814D}, WT - wildtype.

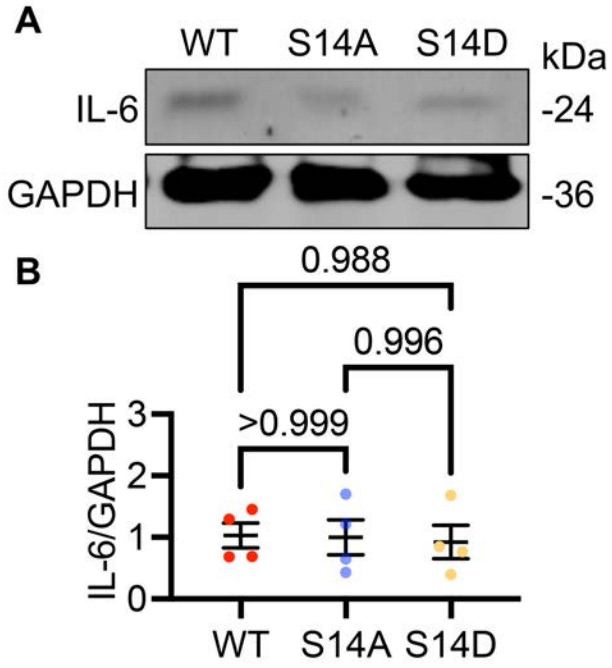

REFERENCES

1. Keefe JA, Navarro-Garcia JA, Ni L, Reilly S, Dobrev D, and Wehrens XHT. In-depth characterization of a mouse model of postoperative atrial fibrillation. *J Cardiovasc Aging.* 2022;2.
2. Siddiquee K, Zhang S, Guida WC, Blaskovich MA, Greedy B, Lawrence HR, et al. Selective chemical probe inhibitor of Stat3, identified through structure-based virtual screening, induces antitumor activity. *Proc Natl Acad Sci U S A.* 2007;104(18):7391-6.
3. Hoffman KA, Villar MJ, Poveda C, Bottazzi ME, Hotez PJ, Tweardy DJ, et al. Signal Transducer and Activator of Transcription-3 Modulation of Cardiac Pathology in Chronic Chagasic Cardiomyopathy. *Front Cell Infect Microbiol.* 2021;11:708325.
4. Li N, and Wehrens XH. Programmed electrical stimulation in mice. *J Vis Exp.* 2010(39):e1730.
5. Navarro-Garcia JA, Bruns F, Moore OM, Tekook MA, Dobrev D, Miyake CY, et al. In Vivo Cardiac Electrophysiology in Mice: Determination of Atrial and Ventricular Arrhythmic Substrates. *Curr Protoc.* 2024;4(2):e994.
6. Luo W, Wang Y, Zhang L, Ren P, Zhang C, Li Y, et al. Critical Role of Cytosolic DNA and Its Sensing Adaptor STING in Aortic Degeneration, Dissection, and Rupture. *Circulation.* 2020;141(1):42-66.
7. Li Y, Ren P, Dawson A, Vasquez HG, Ageedi W, Zhang C, et al. Single-Cell Transcriptome Analysis Reveals Dynamic Cell Populations and Differential Gene Expression Patterns in Control and Aneurysmal Human Aortic Tissue. *Circulation.* 2020;142(14):1374-88.
8. Trapnell C, Cacchiarelli D, Grimsby J, Pokharel P, Li S, Morse M, et al. The dynamics and regulators of cell fate decisions are revealed by pseudotemporal ordering of single cells. *Nature Biotechnology.* 2014;32(4):381-6.
9. Jin S, Guerrero-Juarez CF, Zhang L, Chang I, Ramos R, Kuan CH, et al. Inference and analysis of cell-cell communication using CellChat. *Nat Commun.* 2021;12(1):1088.
10. Jensen EC. Quantitative analysis of histological staining and fluorescence using ImageJ. *Anat Rec (Hoboken).* 2013;296(3):378-81.
11. Yao C, Veleva T, Scott L, Jr., Cao S, Li L, Chen G, et al. Enhanced Cardiomyocyte NLRP3 Inflammasome Signaling Promotes Atrial Fibrillation. *Circulation.* 2018;138(20):2227-42.
12. Wang X, Song J, Yuan Y, Li L, Abu-Taha I, Heijman J, et al. Downregulation of FKBP5 Promotes Atrial Arrhythmogenesis. *Circulation Research.* 2023;133(1):e1-e16.
13. Quick AP, Wang Q, Philippen LE, Barreto-Torres G, Chiang DY, Beavers D, et al. SPEG (Striated Muscle Preferentially Expressed Protein Kinase) Is Essential for Cardiac Function by Regulating Junctional Membrane Complex Activity. *Circ Res.* 2017;120(1):110-9.
14. Campbell HM, Quick AP, Abu-Taha I, Chiang DY, Kramm CF, Word TA, et al. Loss of SPEG Inhibitory Phosphorylation of Ryanodine Receptor Type-2 Promotes Atrial Fibrillation. *Circulation.* 2020;142(12):1159-72.
15. Landstrom AP, Yang Q, Sun B, Perelli RM, Bidzimou M-T, Zhang Z, et al. Reduction in Junctophilin 2 Expression in Cardiac Nodal Tissue Results in Intracellular Calcium-Driven Increase in Nodal Cell Automaticity. *Circulation: Arrhythmia and Electrophysiology.* 2023;16(2):e010858.
16. Chelu MG, Sarma S, Sood S, Wang S, van Oort RJ, Skapura DG, et al. Calmodulin kinase II-mediated sarcoplasmic reticulum Ca2+ leak promotes atrial fibrillation in mice. *J Clin Invest.* 2009;119(7):1940-51.
17. Picht E, Zima AV, Blatter LA, and Bers DM. SparkMaster: automated calcium spark analysis with ImageJ. *Am J Physiol Cell Physiol.* 2007;293(3):C1073-81.

18. Motulsky HJ, and Brown RE. Detecting outliers when fitting data with nonlinear regression - a new method based on robust nonlinear regression and the false discovery rate. *BMC Bioinformatics.* 2006;7:123.
19. Huang Z, Chen XJ, Qian C, Dong Q, Ding D, Wu QF, et al. Signal Transducer and Activator of Transcription 3/MicroRNA-21 Feedback Loop Contributes to Atrial Fibrillation by Promoting Atrial Fibrosis in a Rat Sterile Pericarditis Model. *Circ Arrhythm Electrophysiol.* 2016;9(7):e003396.
20. Liao J, Zhang S, Yang S, Lu Y, Lu K, Wu Y, et al. Interleukin-6-Mediated-Ca(2+) Handling Abnormalities Contributes to Atrial Fibrillation in Sterile Pericarditis Rats. *Front Immunol.* 2021;12:758157.
21. Liu Y, Wu F, Wu Y, Elliott M, Zhou W, Deng Y, et al. Mechanism of IL-6-related spontaneous atrial fibrillation after coronary artery grafting surgery: IL-6 knockout mouse study and human observation. *Transl Res.* 2021;233:16-31.
22. Li X, Wu X, Chen X, Peng S, Chen S, Zhou G, et al. Selective blockade of interleukin 6 trans-signaling depresses atrial fibrillation. *Heart Rhythm.* 2023.
23. Lin AE, Bapat AC, Xiao L, Niroula A, Ye J, Wong WJ, et al. Clonal Hematopoiesis of Indeterminate Potential With Loss of Tet2 Enhances Risk for Atrial Fibrillation Through Nlrp3 Inflammasome Activation. *Circulation.* 2024.
24. Chin CG, Chen YC, Lin YK, Lu YY, Cheng WL, Chung CC, et al. Effect of macrophage migration inhibitory factor on pulmonary vein arrhythmogenesis through late sodium current. *Europace.* 2023;25(2):698-706.
25. Heijman J, Muna AP, Veleva T, Molina CE, Sutanto H, Tekook M, et al. Atrial Myocyte NLRP3/CaMKII Nexus Forms a Substrate for Postoperative Atrial Fibrillation. *Circ Res.* 2020;127(8):1036-55.
