# Supplementary material for: Macrophage-mediated IL-6 signaling drives ryanodine receptor–2 calcium leak in postoperative atrial fibrillation
Source: J Clin Invest. 2025 Mar 6;135(9):e187711. doi: 10.1172/JCI187711 (PMC12043083; doi:10.1172/JCI187711)

Unedited gels

→ denotes band of interest

▭ denotes representative blot used in figure

X denotes lanes excluded from the analyses

········ denotes where blots were cut

L denote ladder

Full unedited gel for Figure 3A

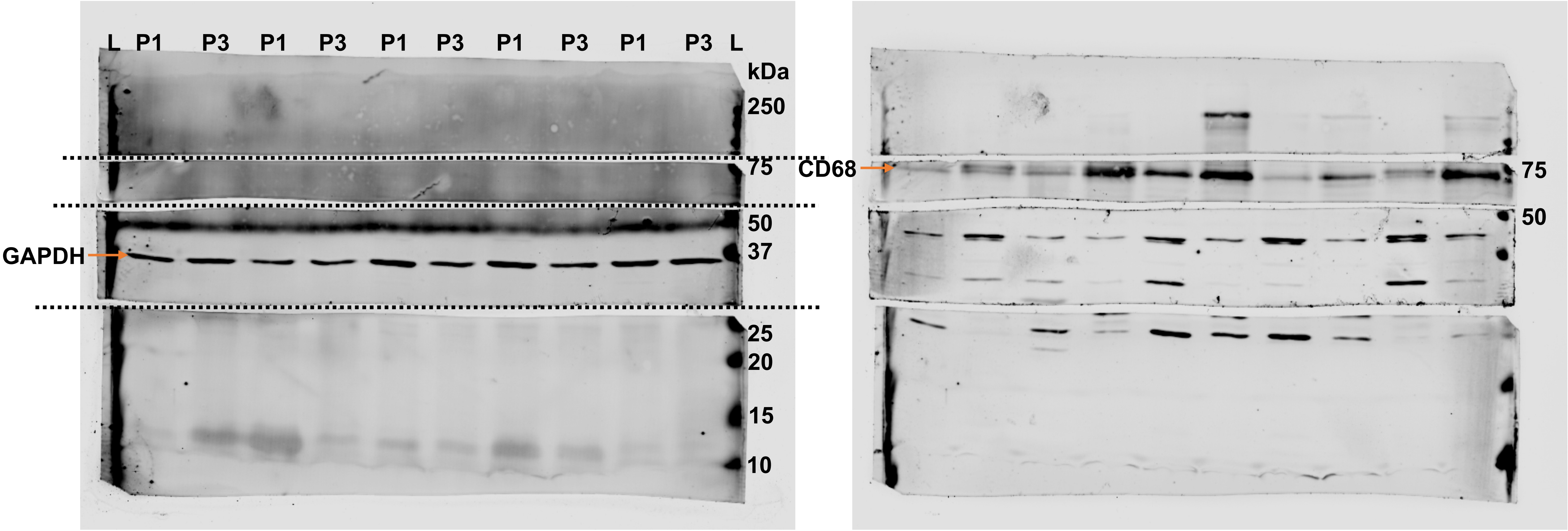

P1 = postoperative day 1
P3 = postoperative day 3

Full unedited gel for Figure 3A

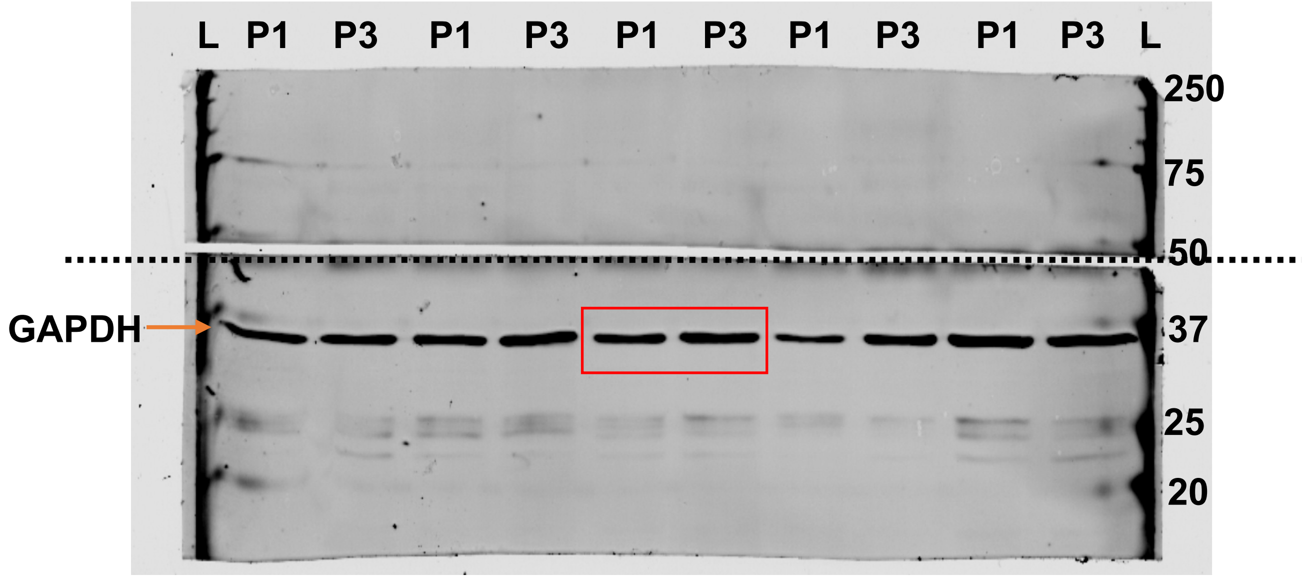

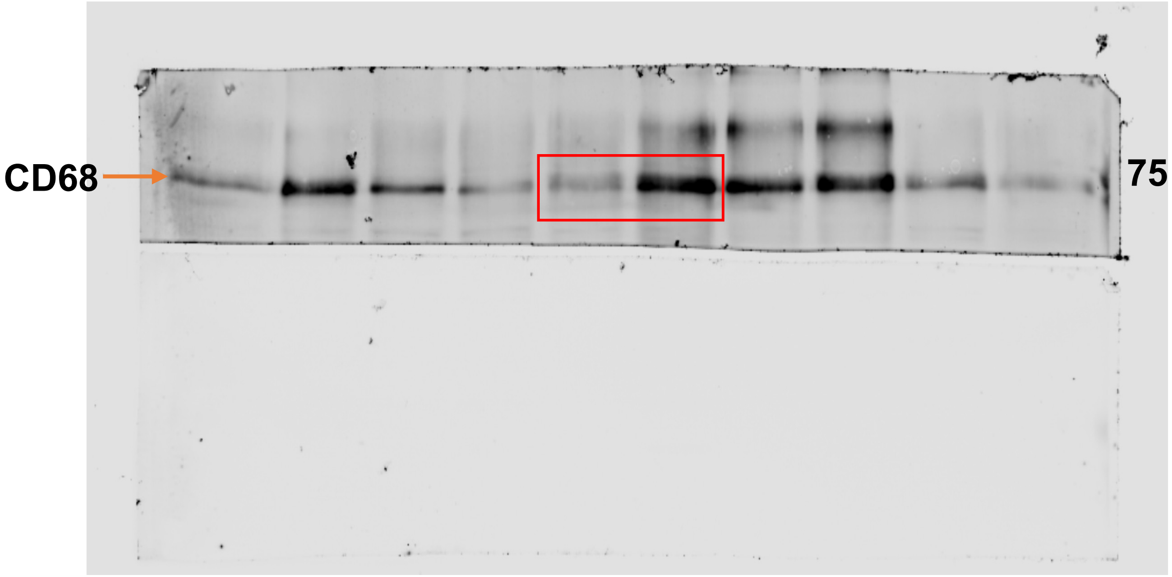

P1 = postoperative day 1
P3 = postoperative day 3

Full unedited gel for Figure 3B

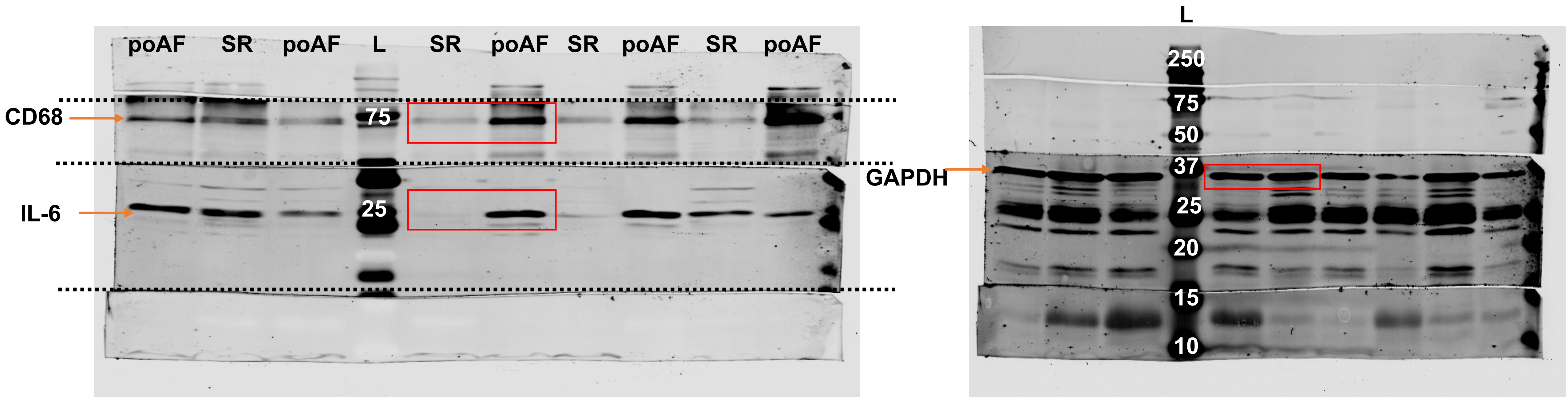

Full unedited gel for Figure 3B

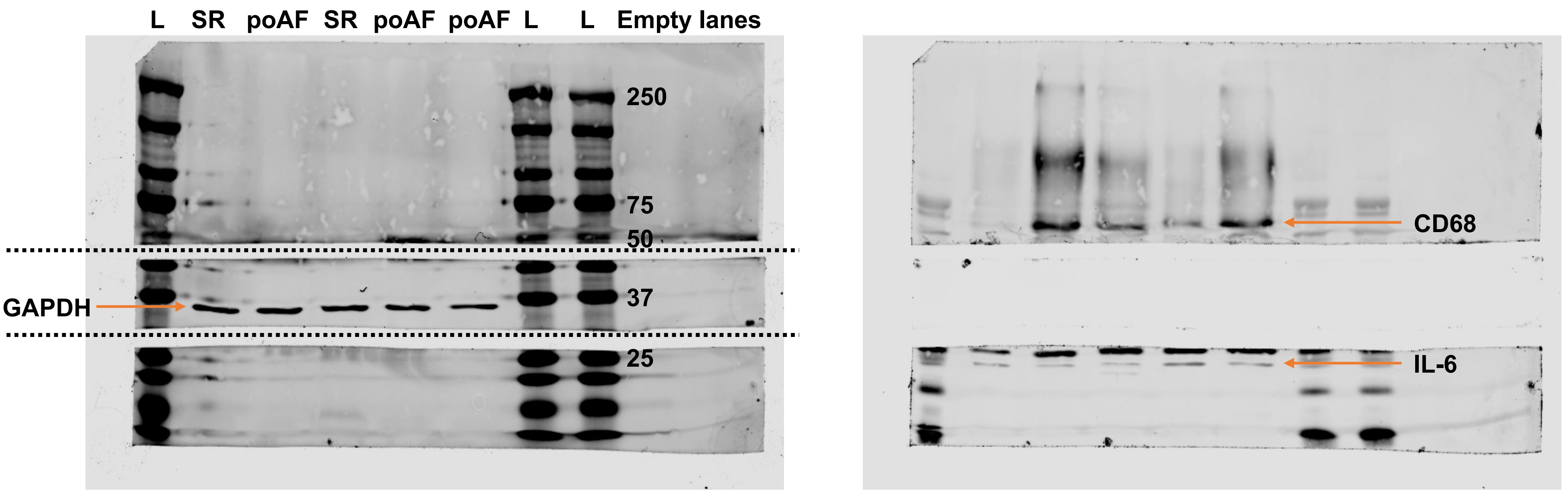

Full unedited gel for Figure 3F

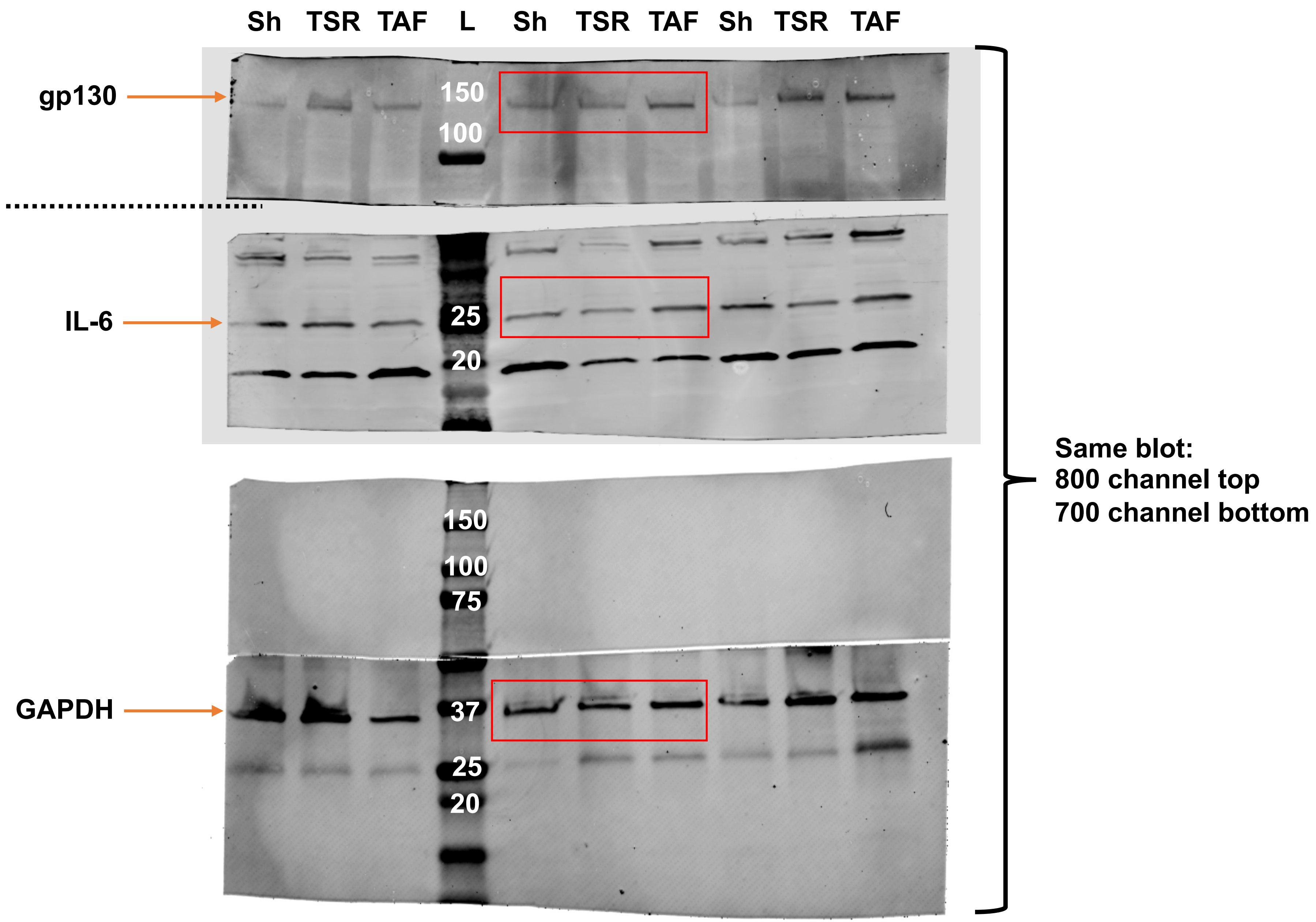

Full unedited gel for Figure 3F

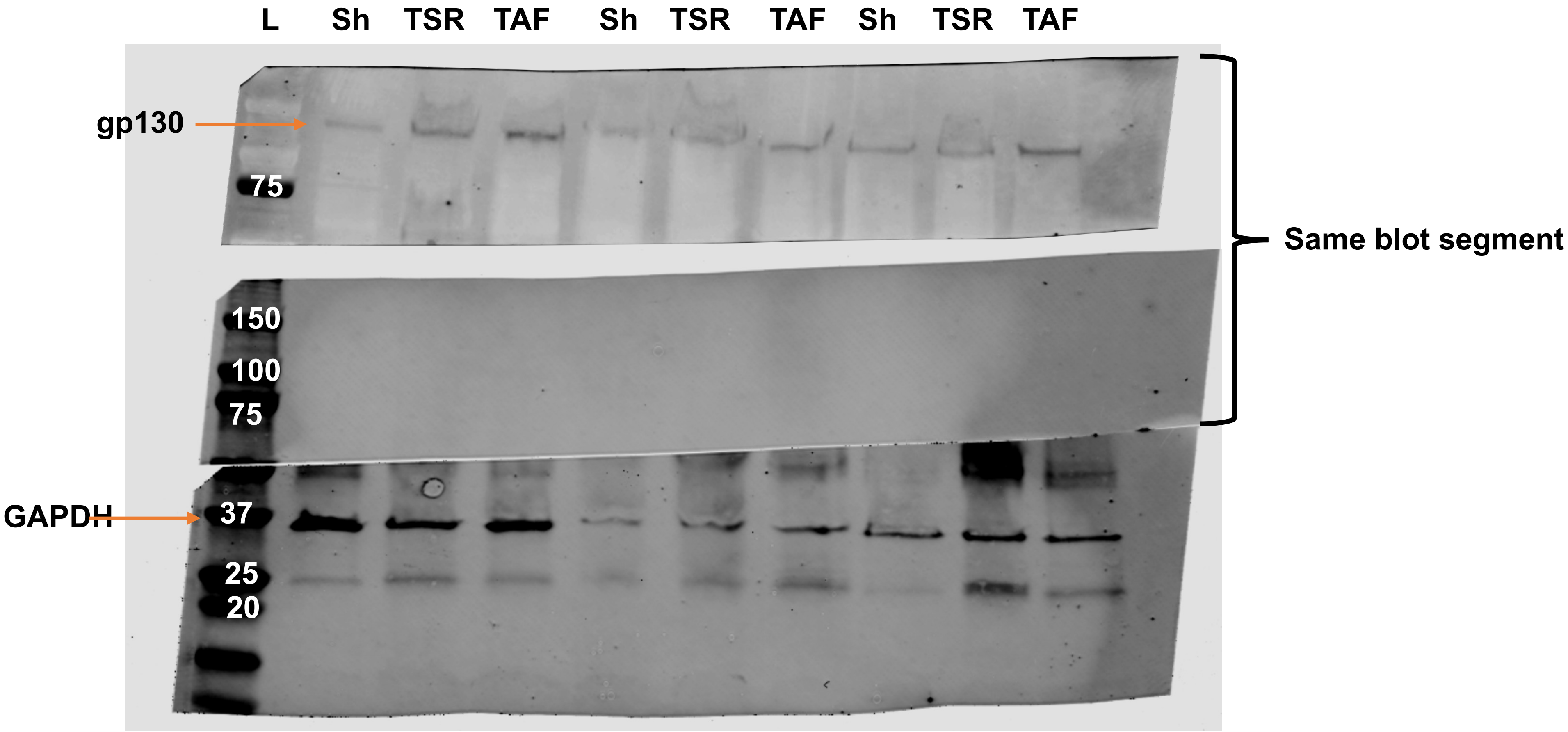

Full unedited gel for Figure 3F

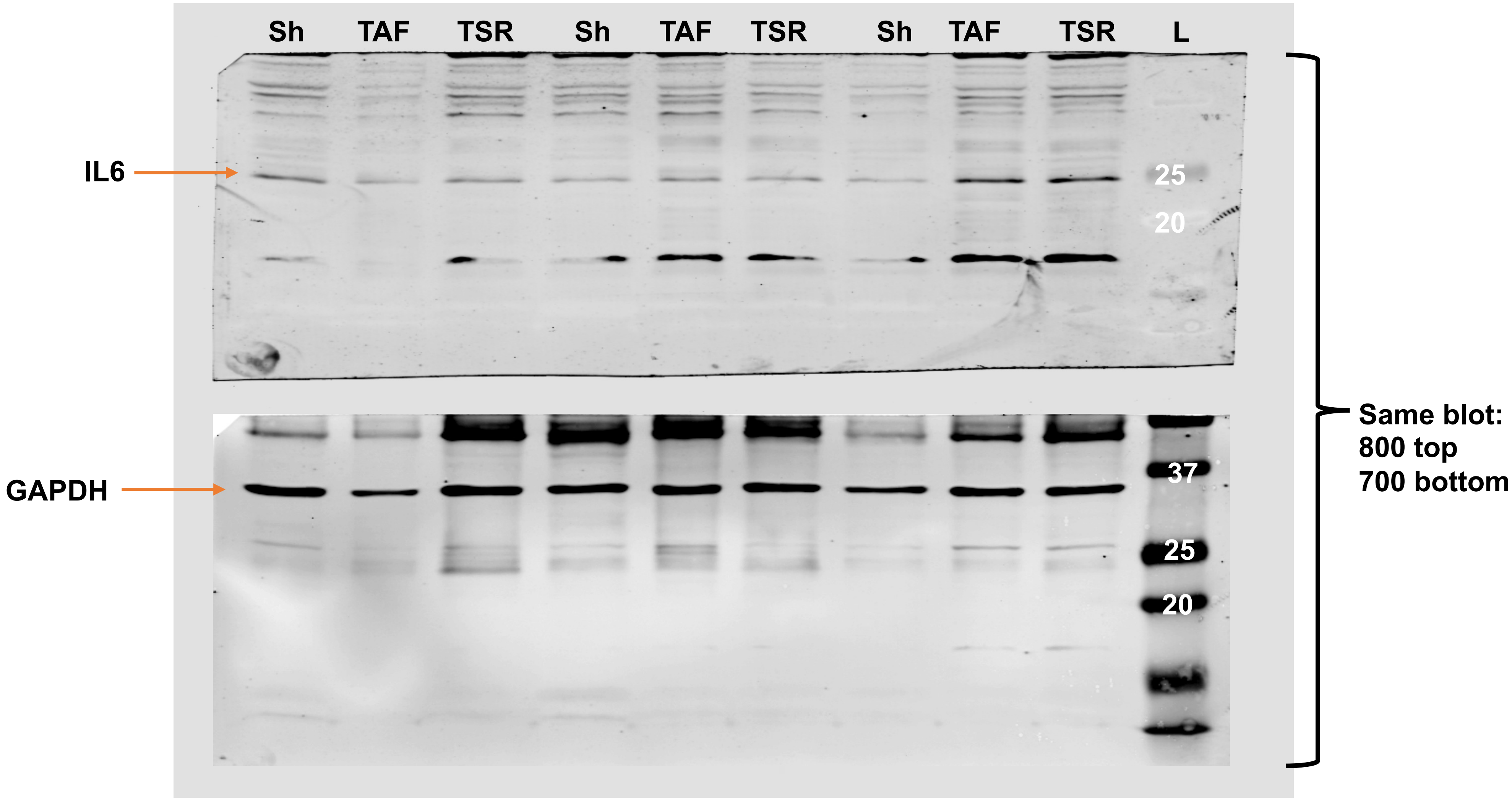

Full unedited gel for Figure 3G

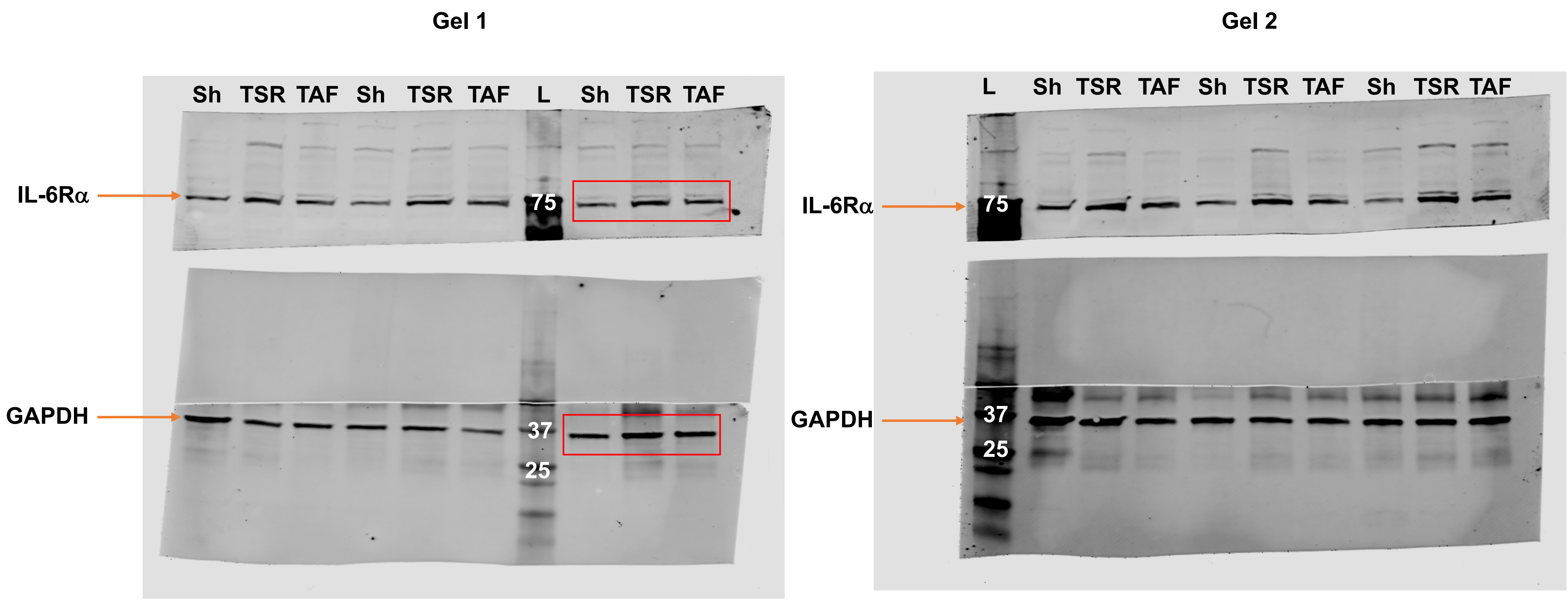

Full unedited gel for 4F: gel 1

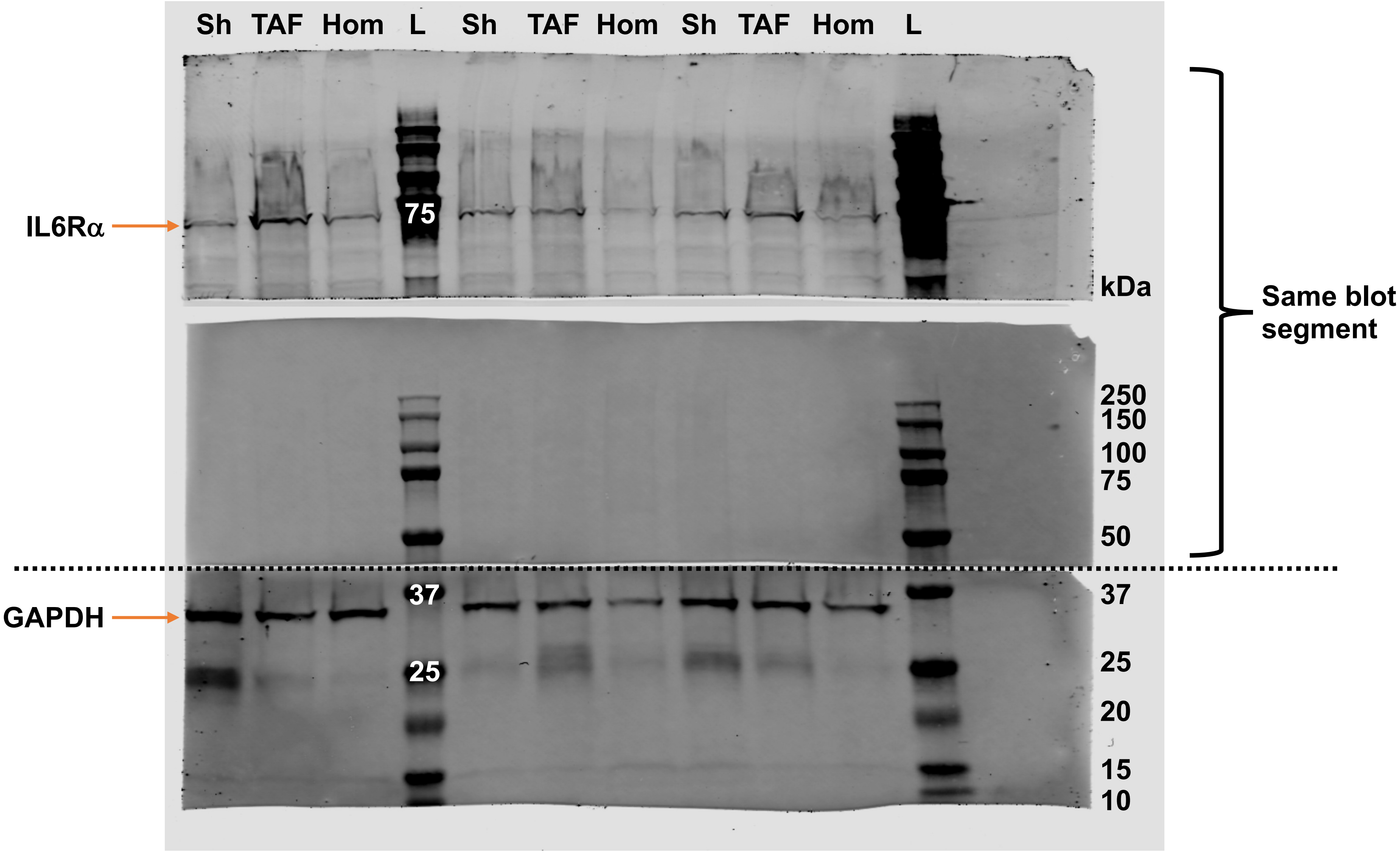

Full unedited gel for 4F: gel 2

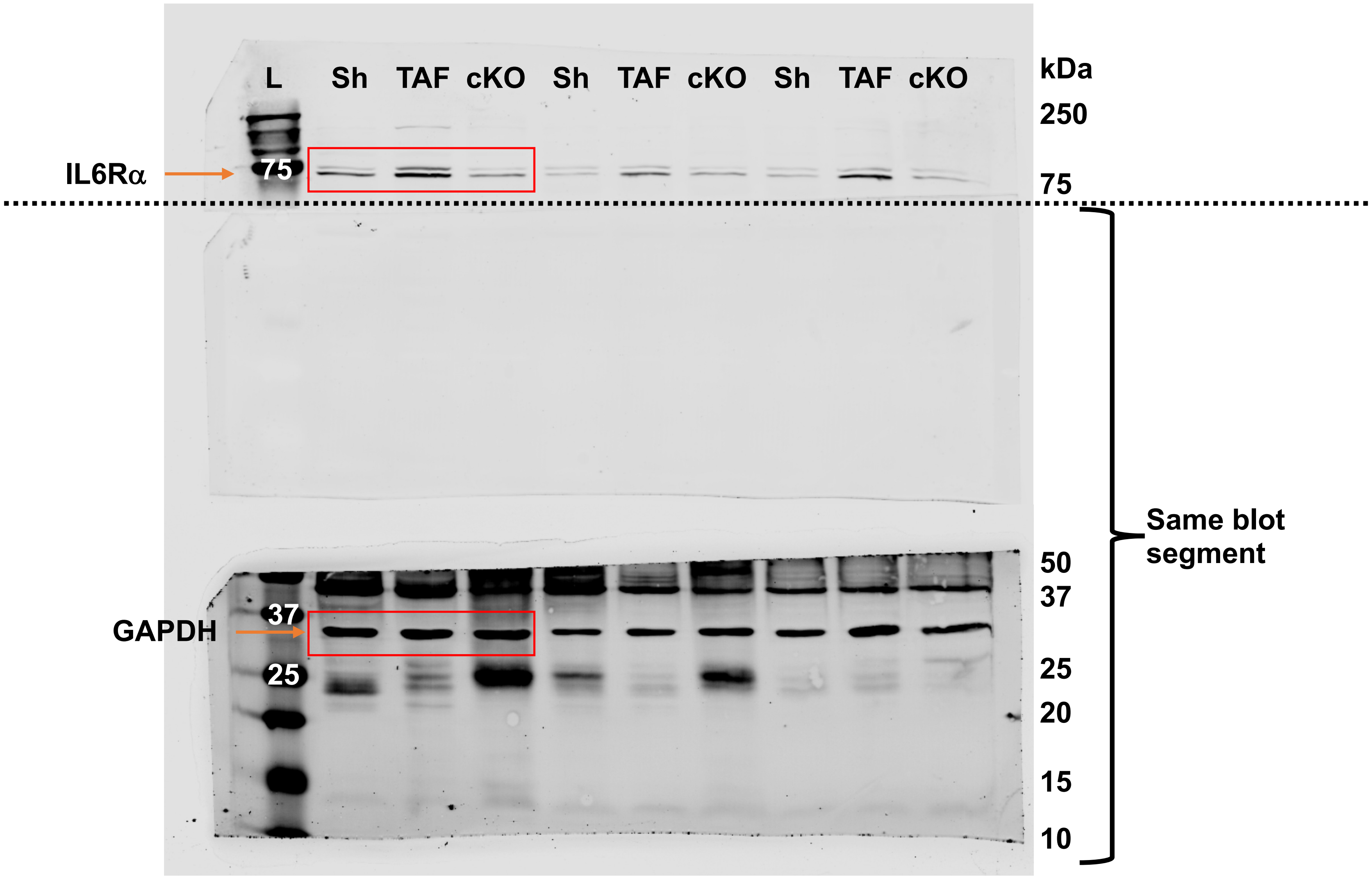

Full unedited gel for Figure 4H

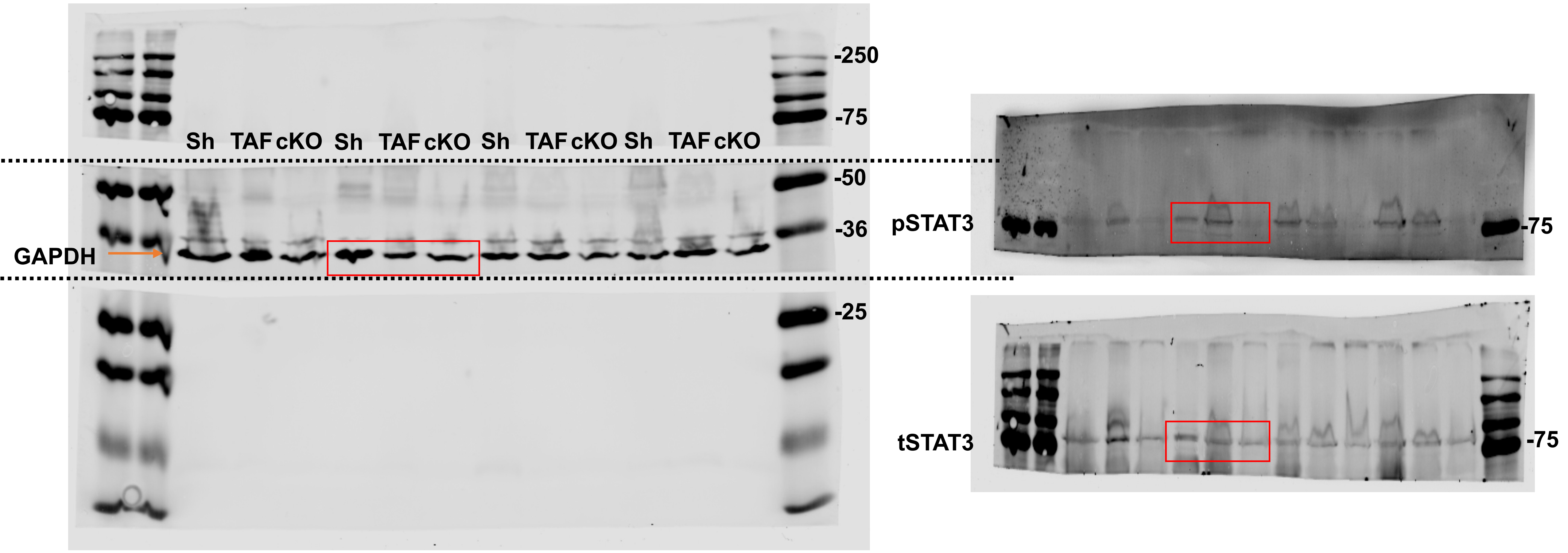

Full unedited gel for Figure 5A: gel 1

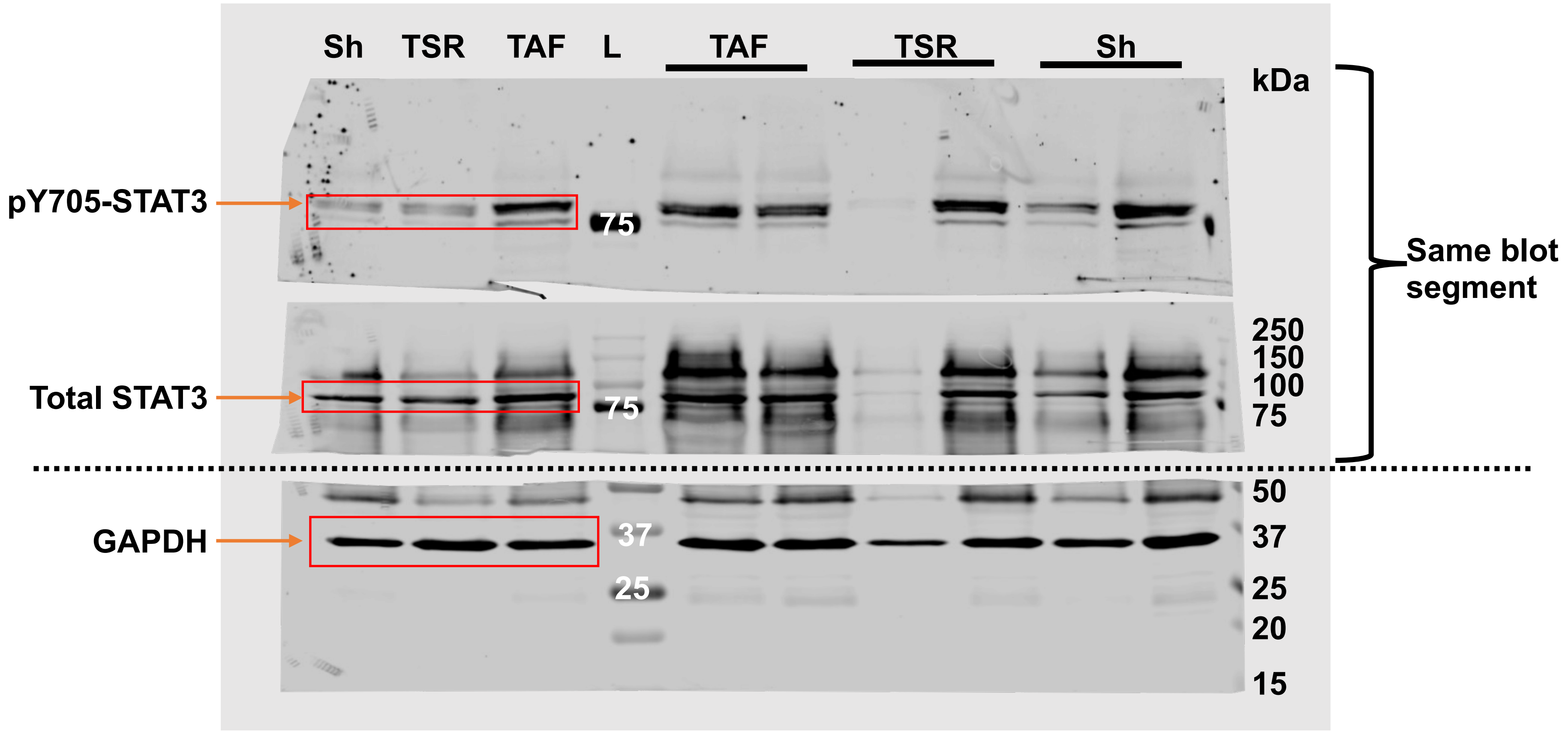

Full unedited gel for Figure 5A: gel 2

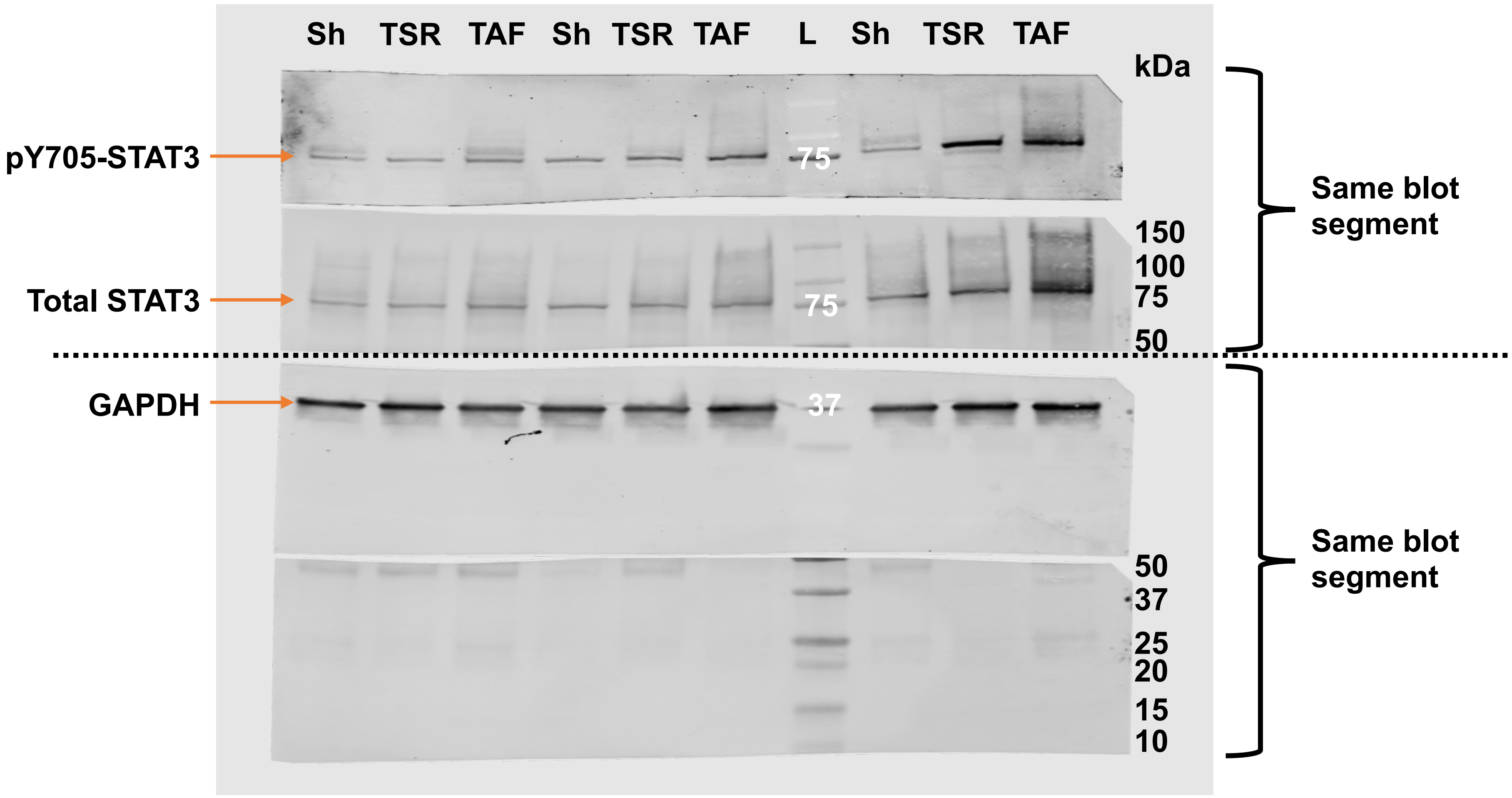

Full unedited gel for Figure 5B: gel 1

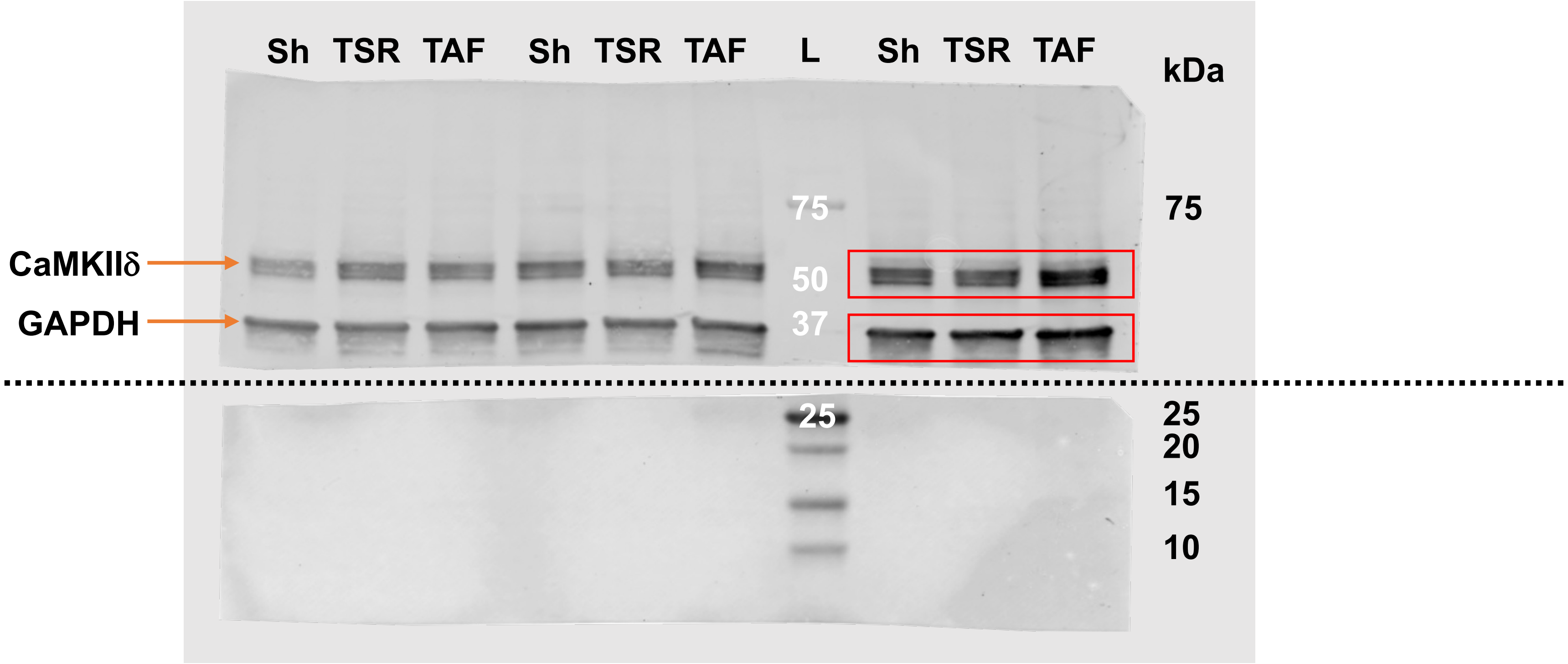

Full unedited gel for Figure 5B: gel 2

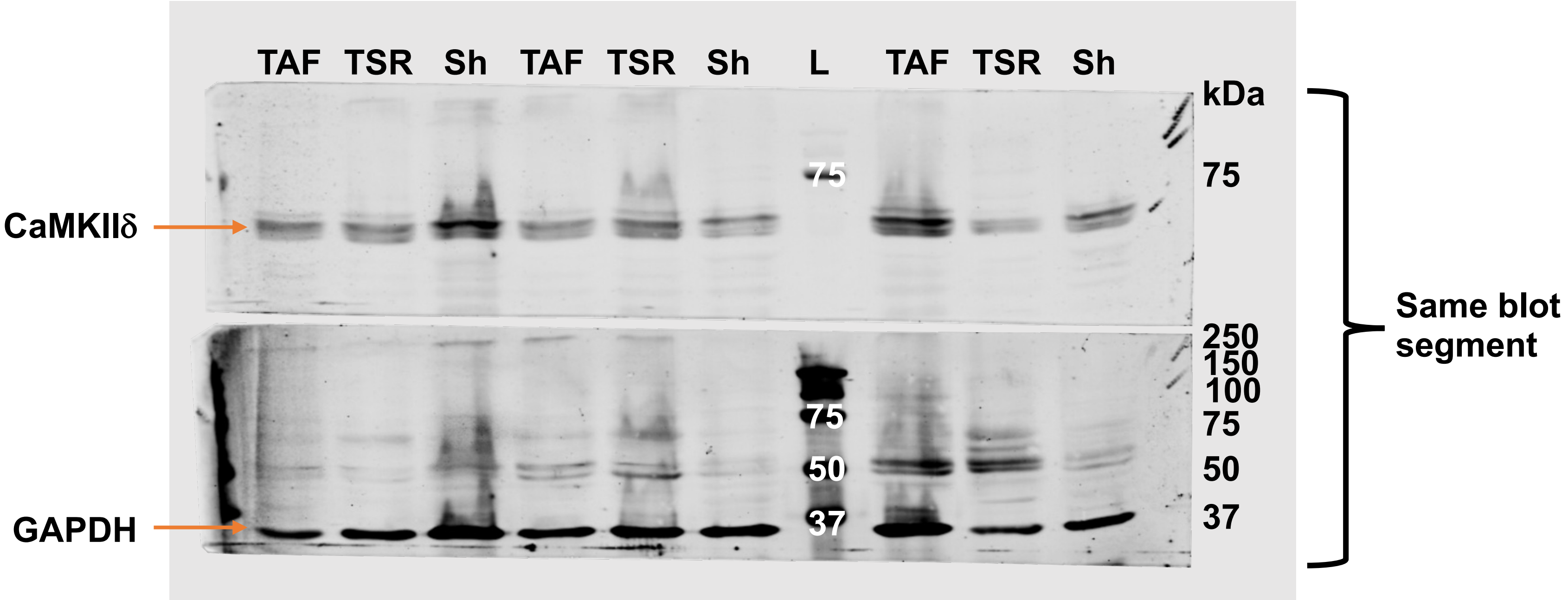

Full unedited gel for Figure 5C: gel 1

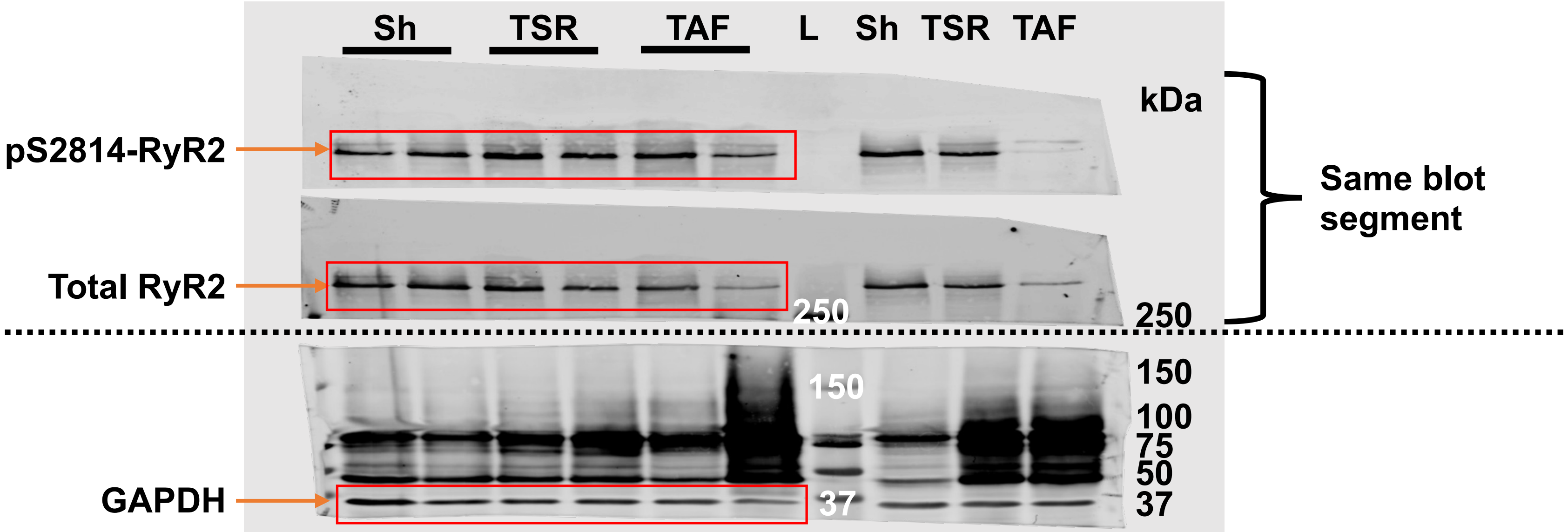

Full unedited gel for Figure 5C: gel 2

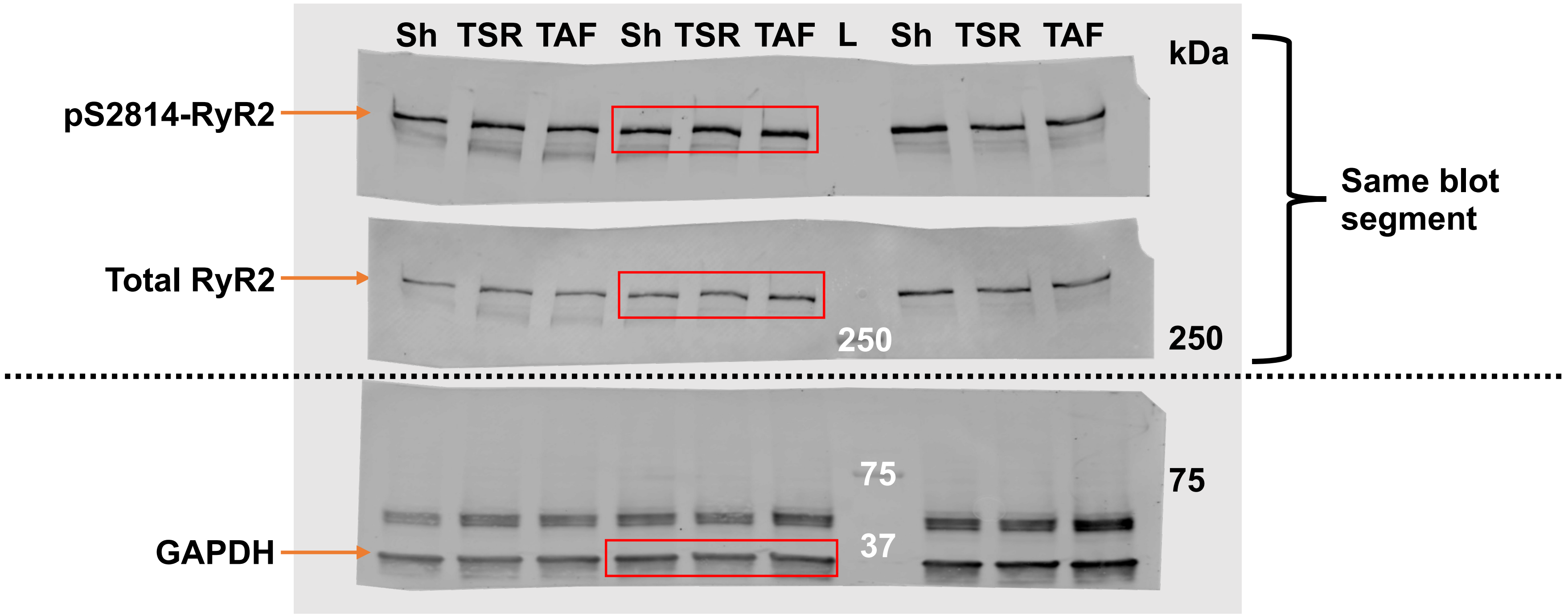

Full unedited gel for Figure 7B

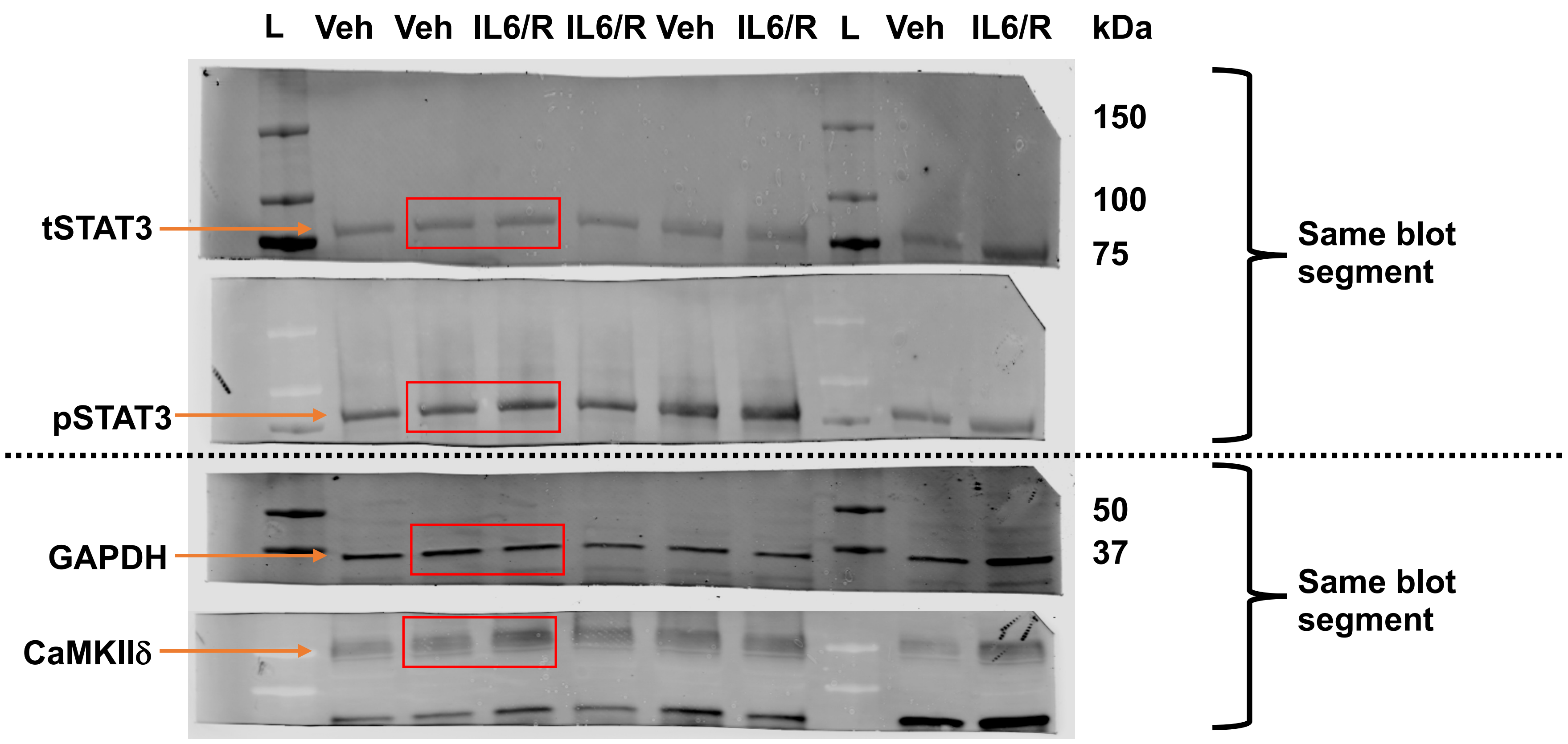

Full unedited gels for Figure S9A: gel 1

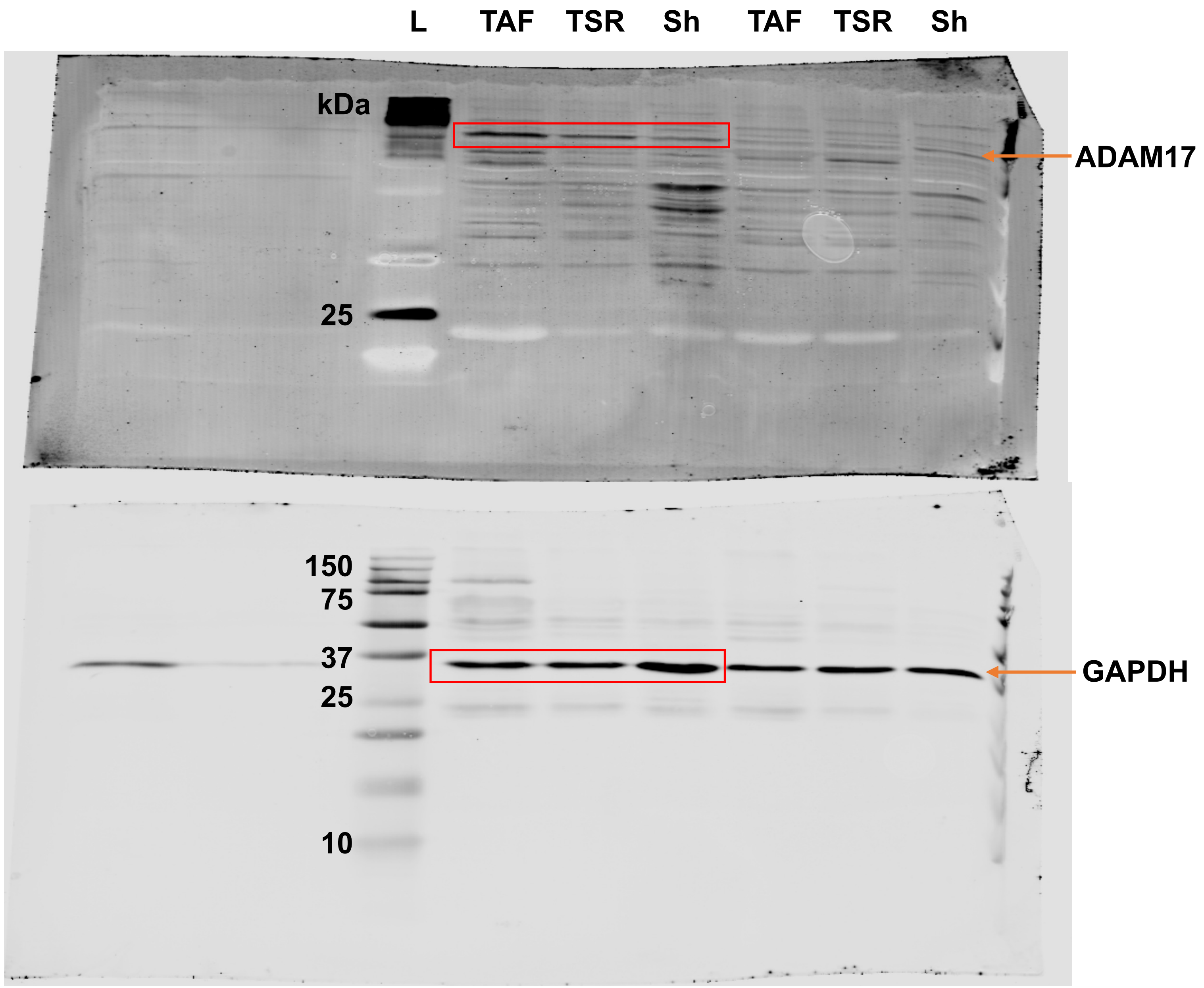

Full unedited gels for Figure S9A: gel 2

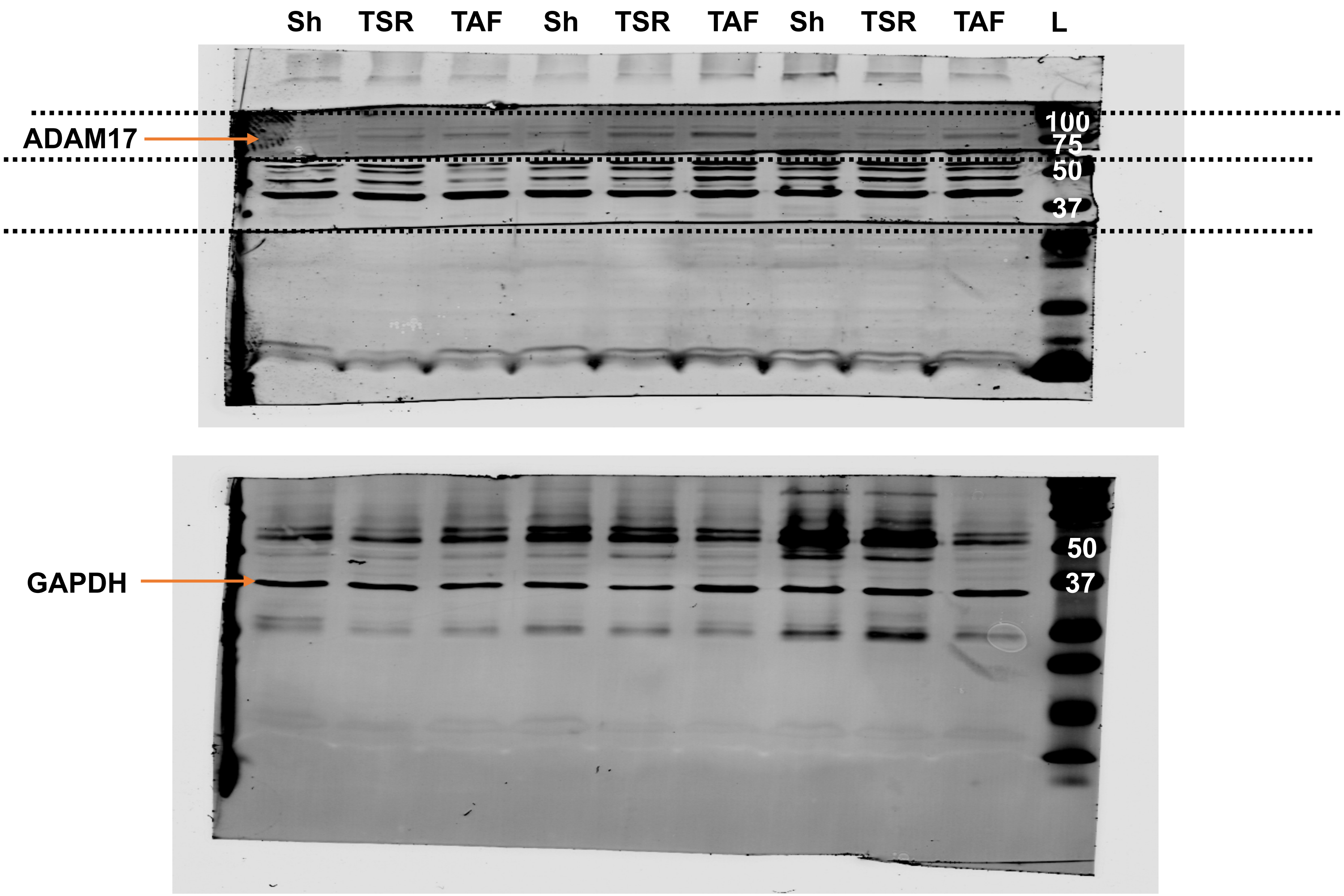

Full unedited gels for Figure S9D: gel 1

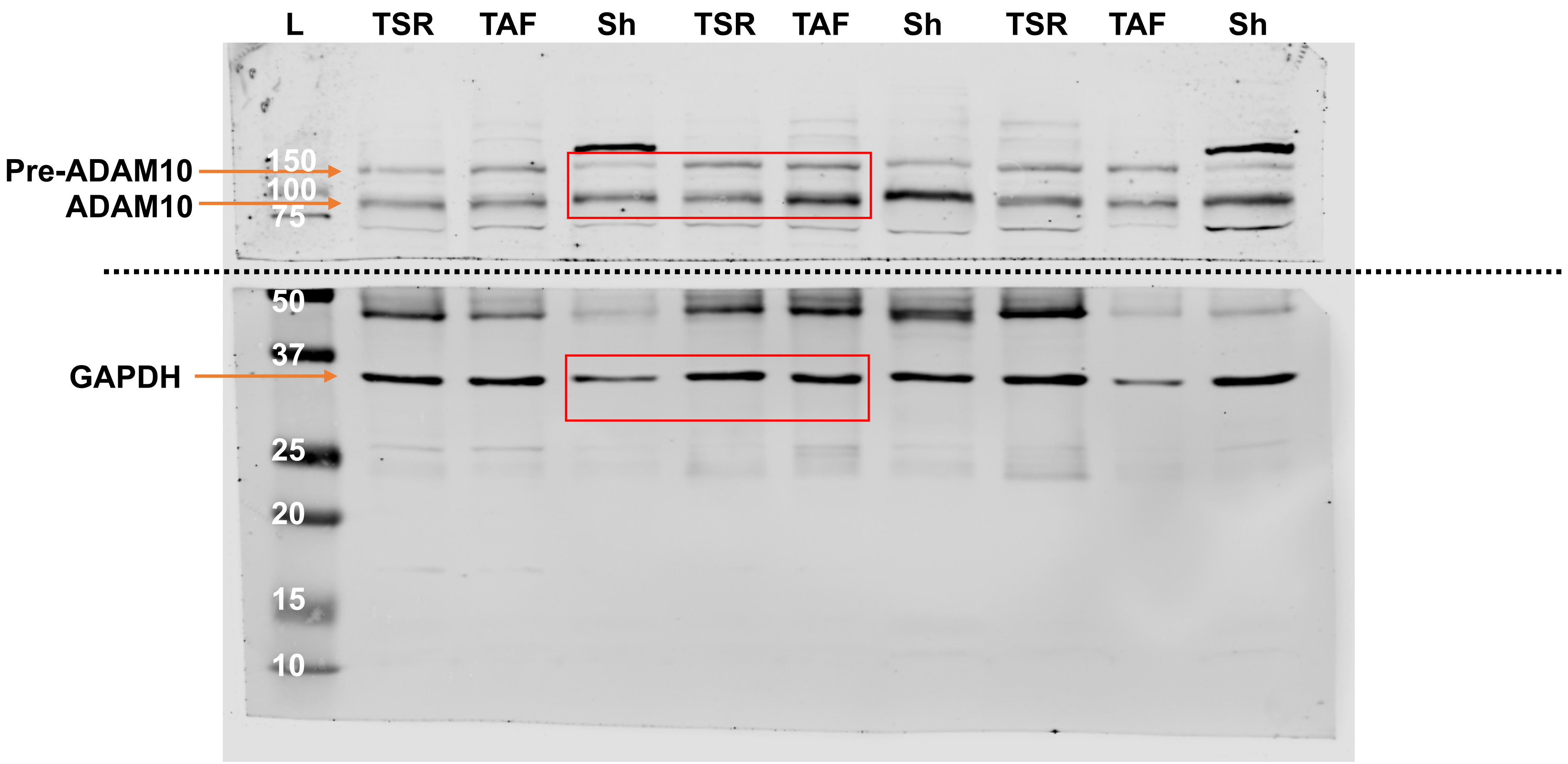

Full unedited gels for Figure S9D: gel 2

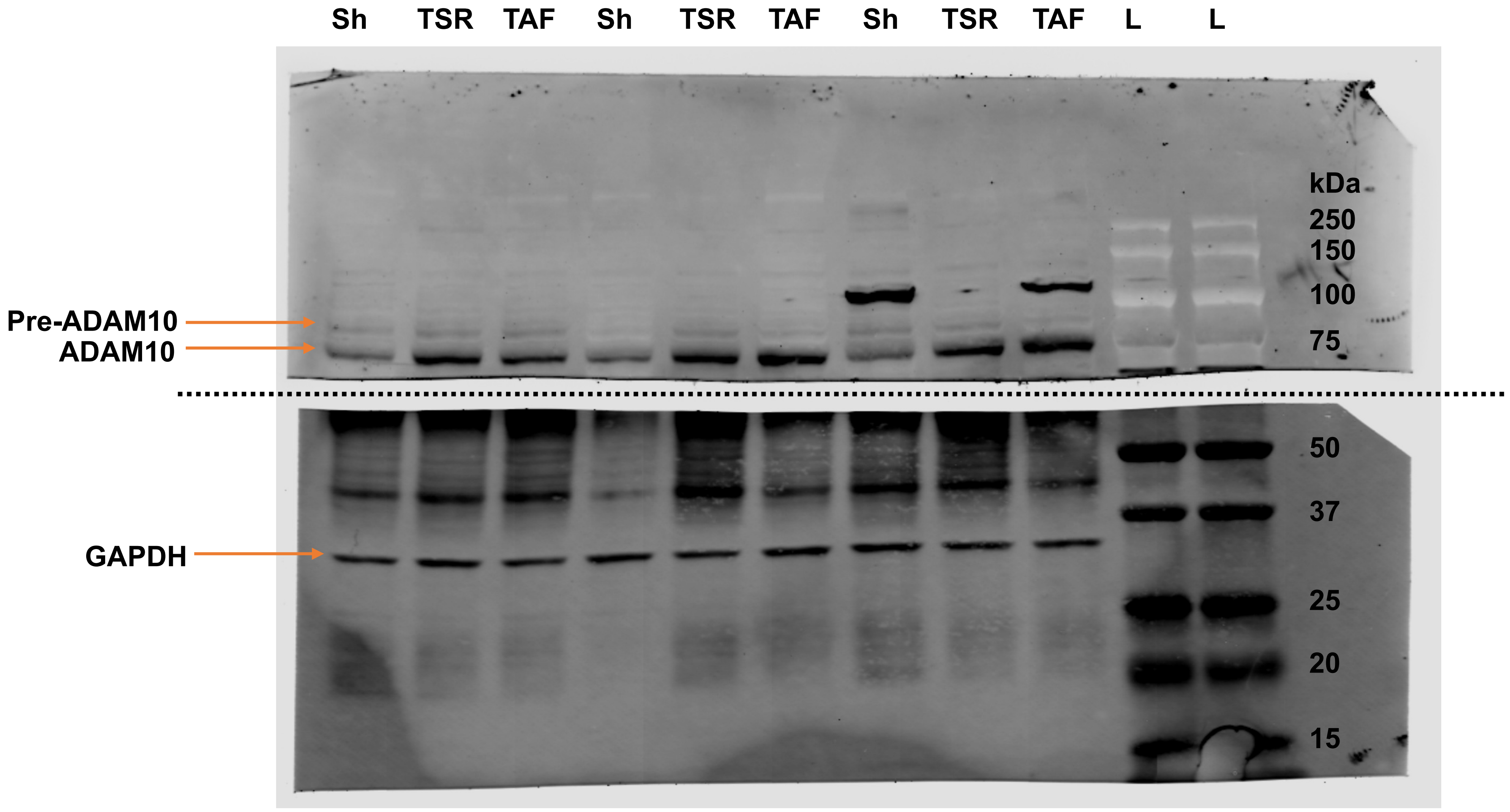

Full unedited gel for Figure S10B

IL-6

Gel 1

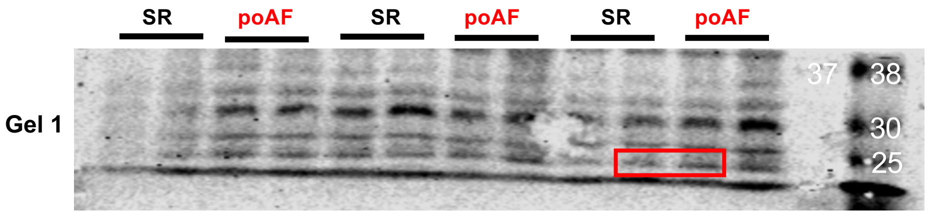

Gel 2

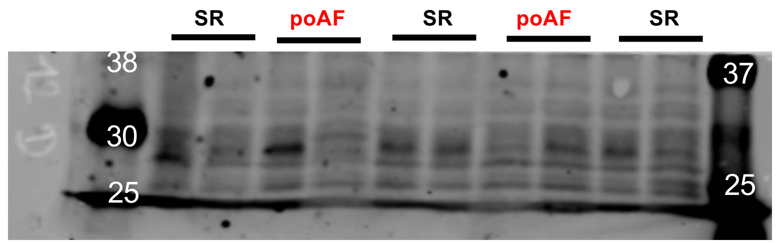

Full unedited gel for Figure S10E

IL-6Rα

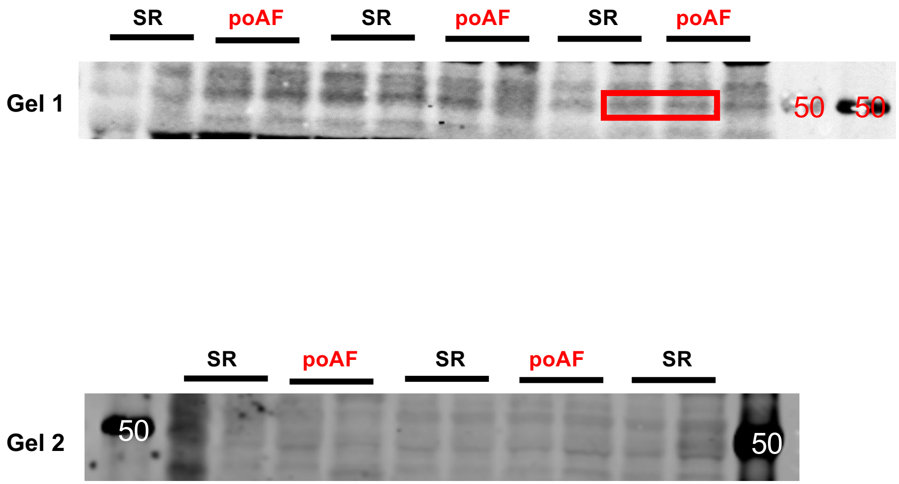

Full unedited gel for Figure S10B, S10E

Revert 700 for IL-6 and IL-6Rα

Gel 1

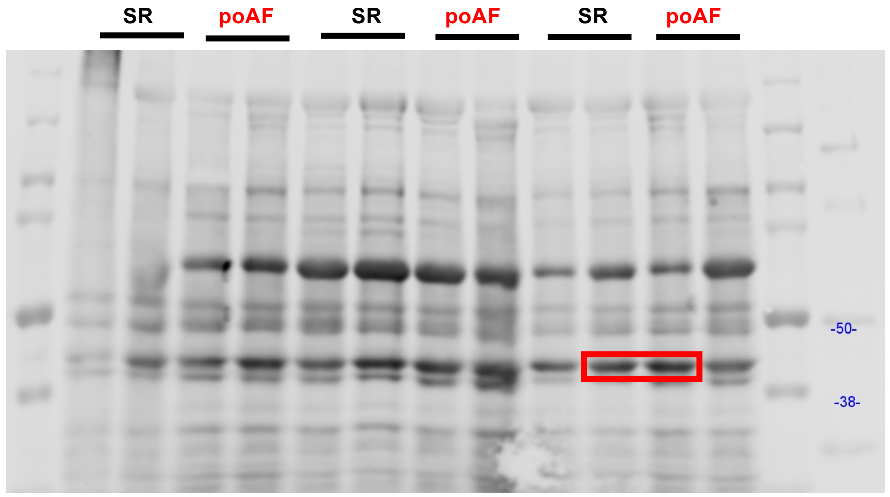

Gel 2

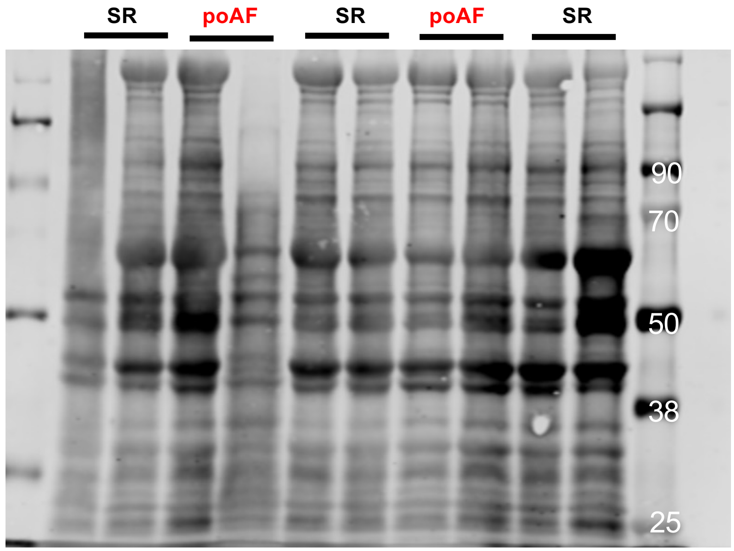

Full unedited gel for Figure S10H

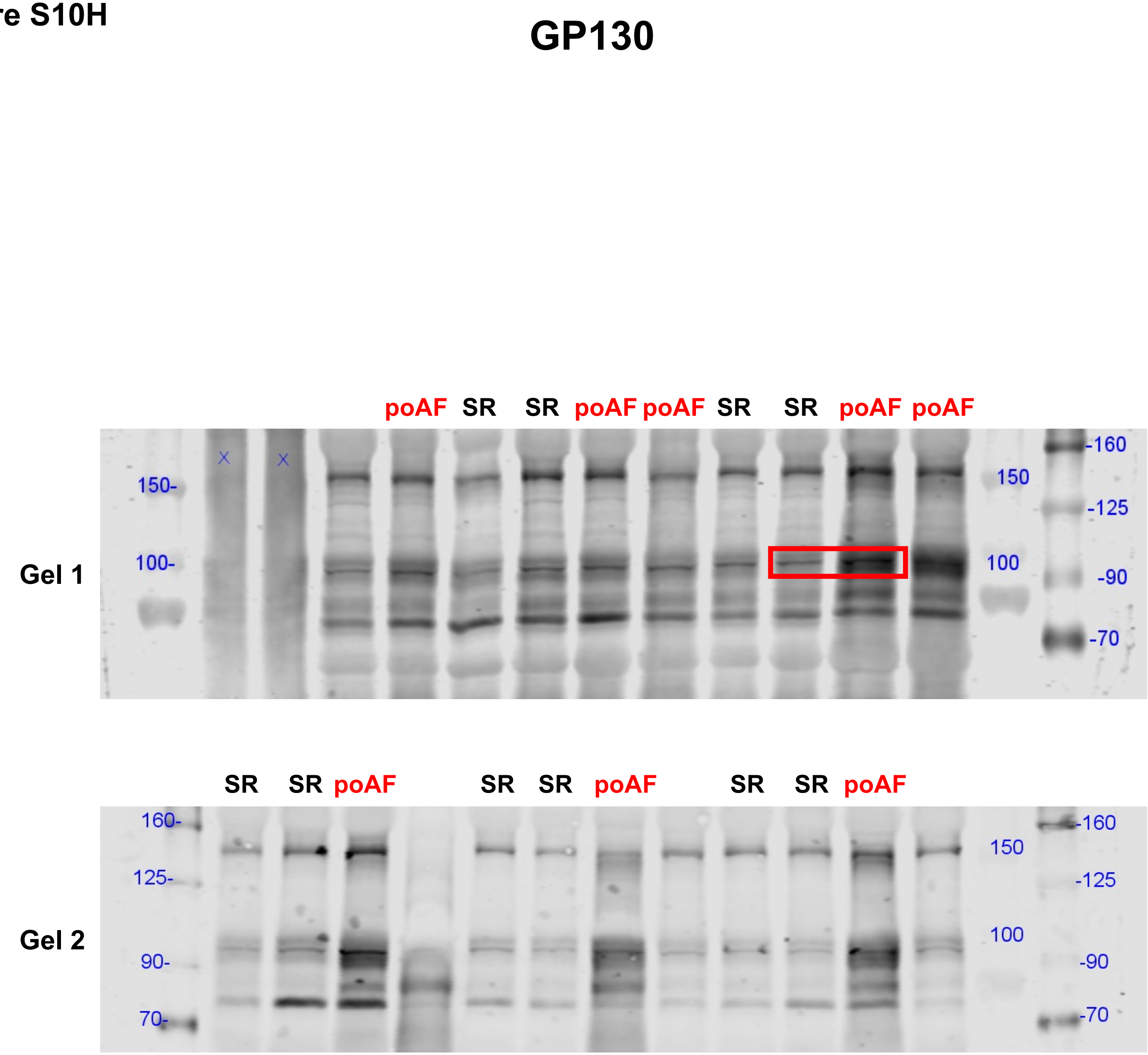

Full unedited gel for Figure S10H

Revert 700 for GP130

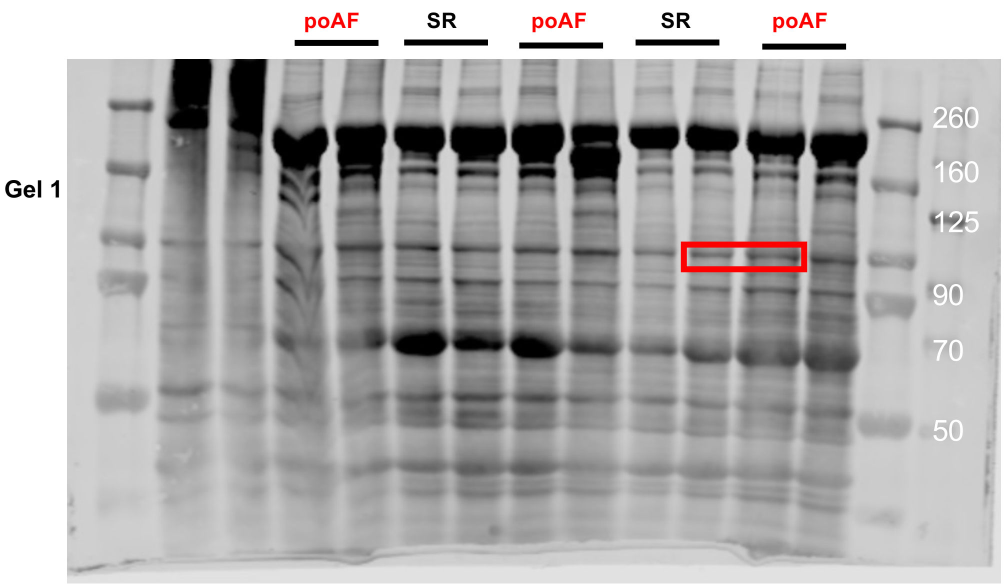

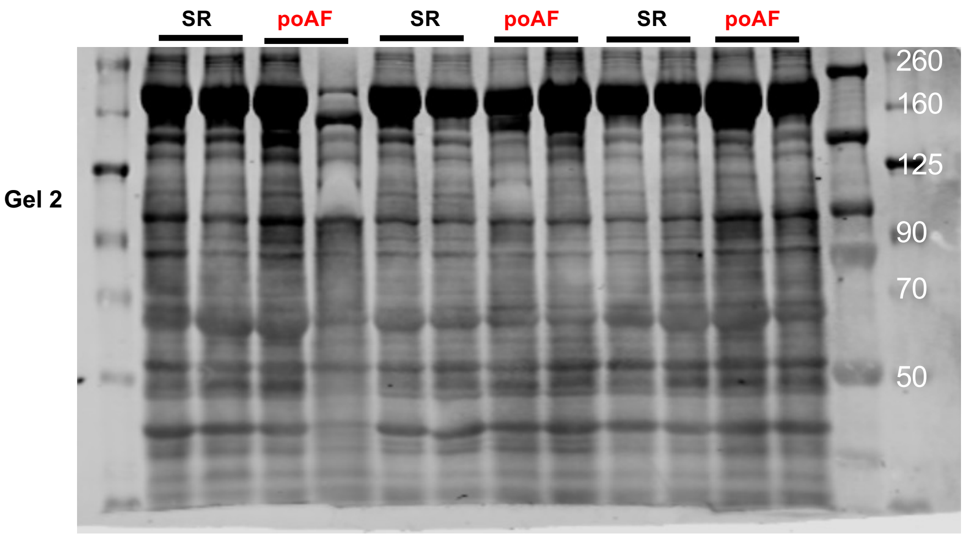

Full unedited gel for Figure S11A

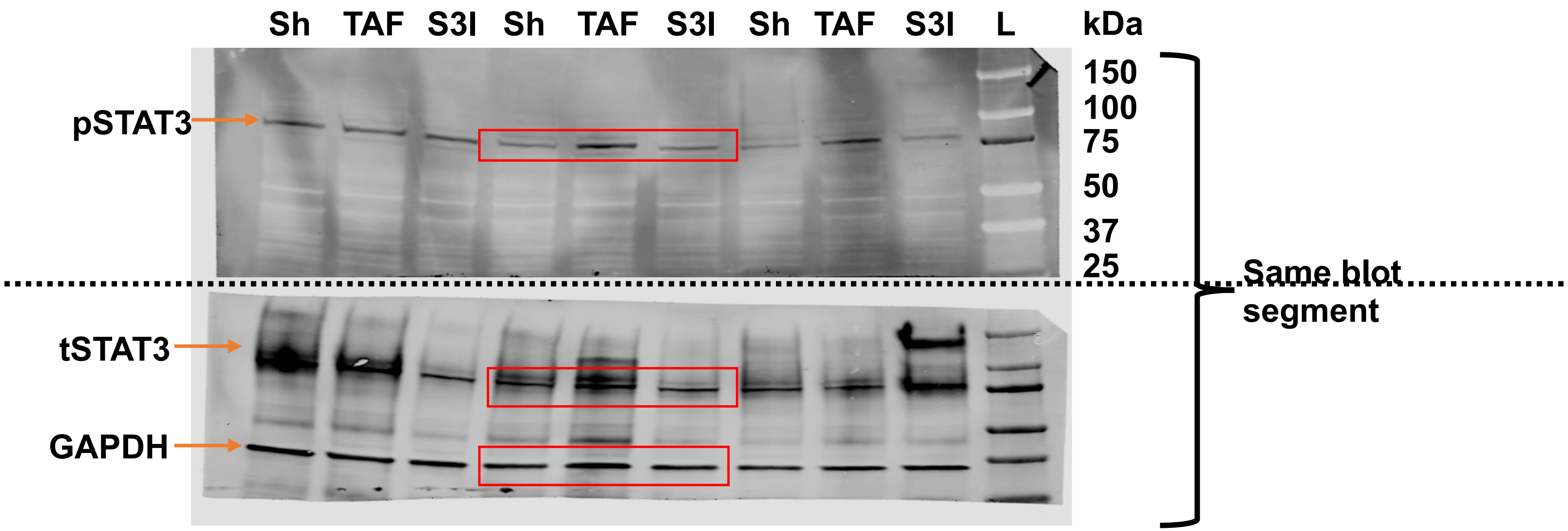

Full unedited gel for Figure S11A

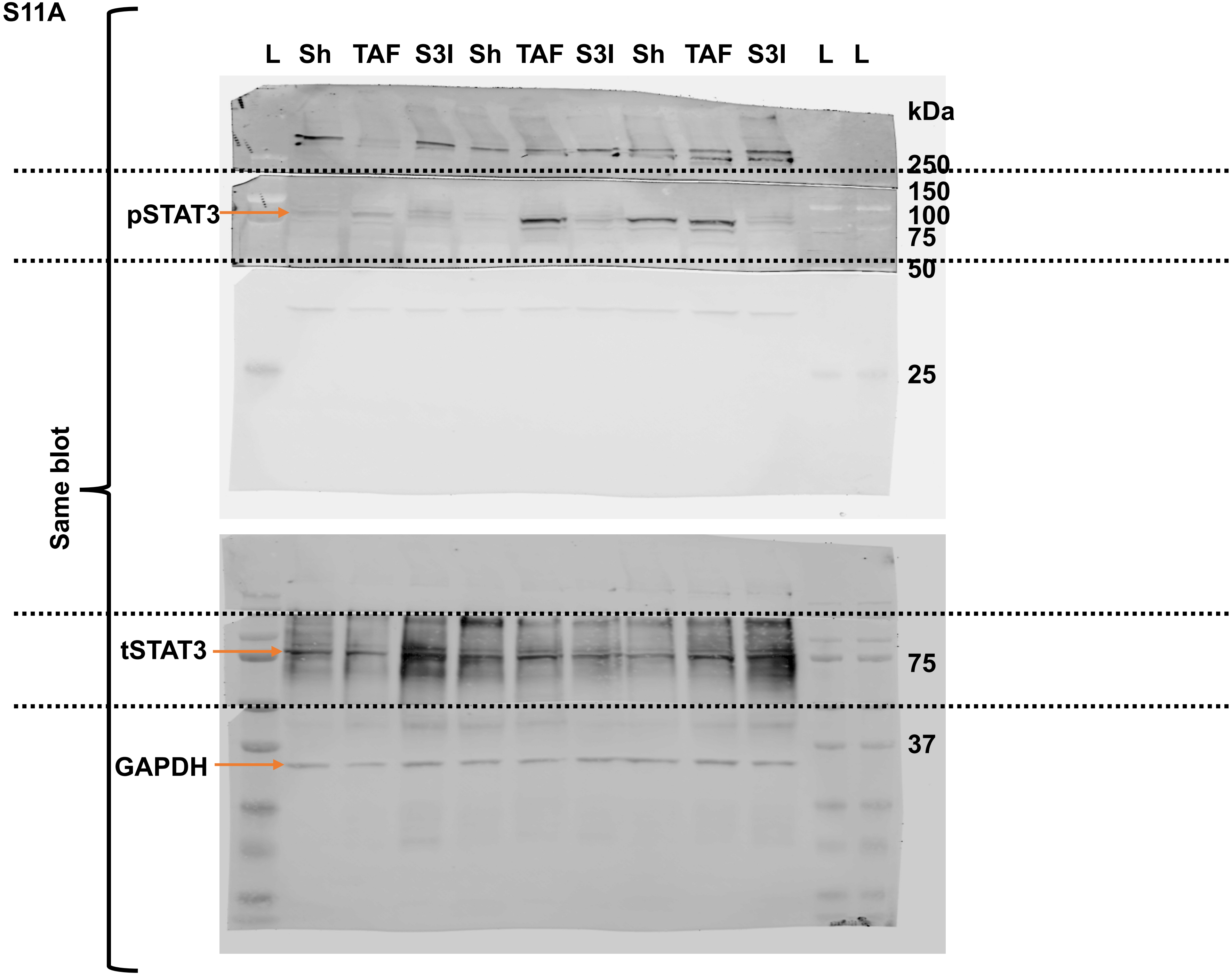

Full unedited gel for Figure S11B

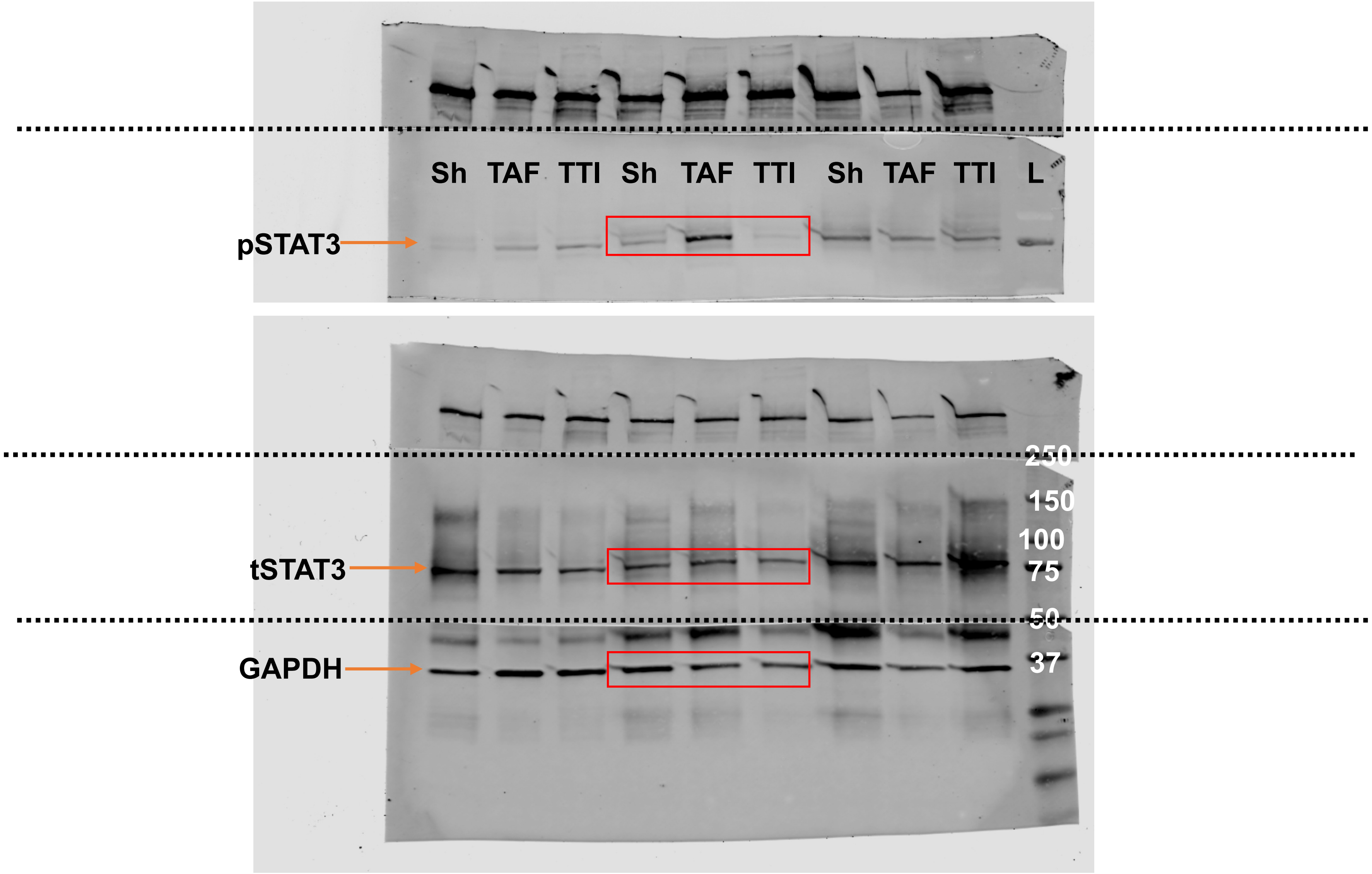

Full unedited gel for Figure S11B

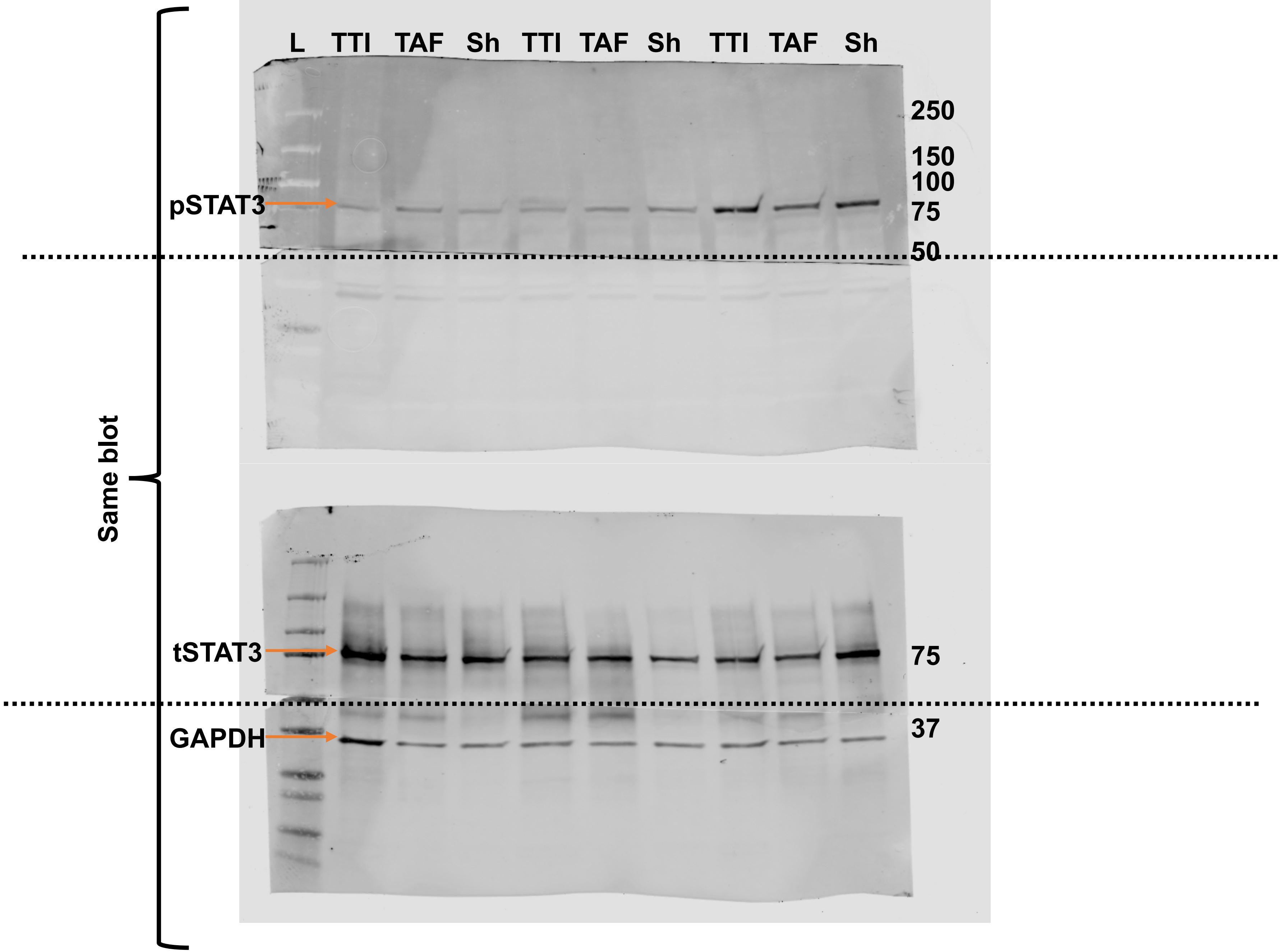

Full unedited gel for Figure S11C

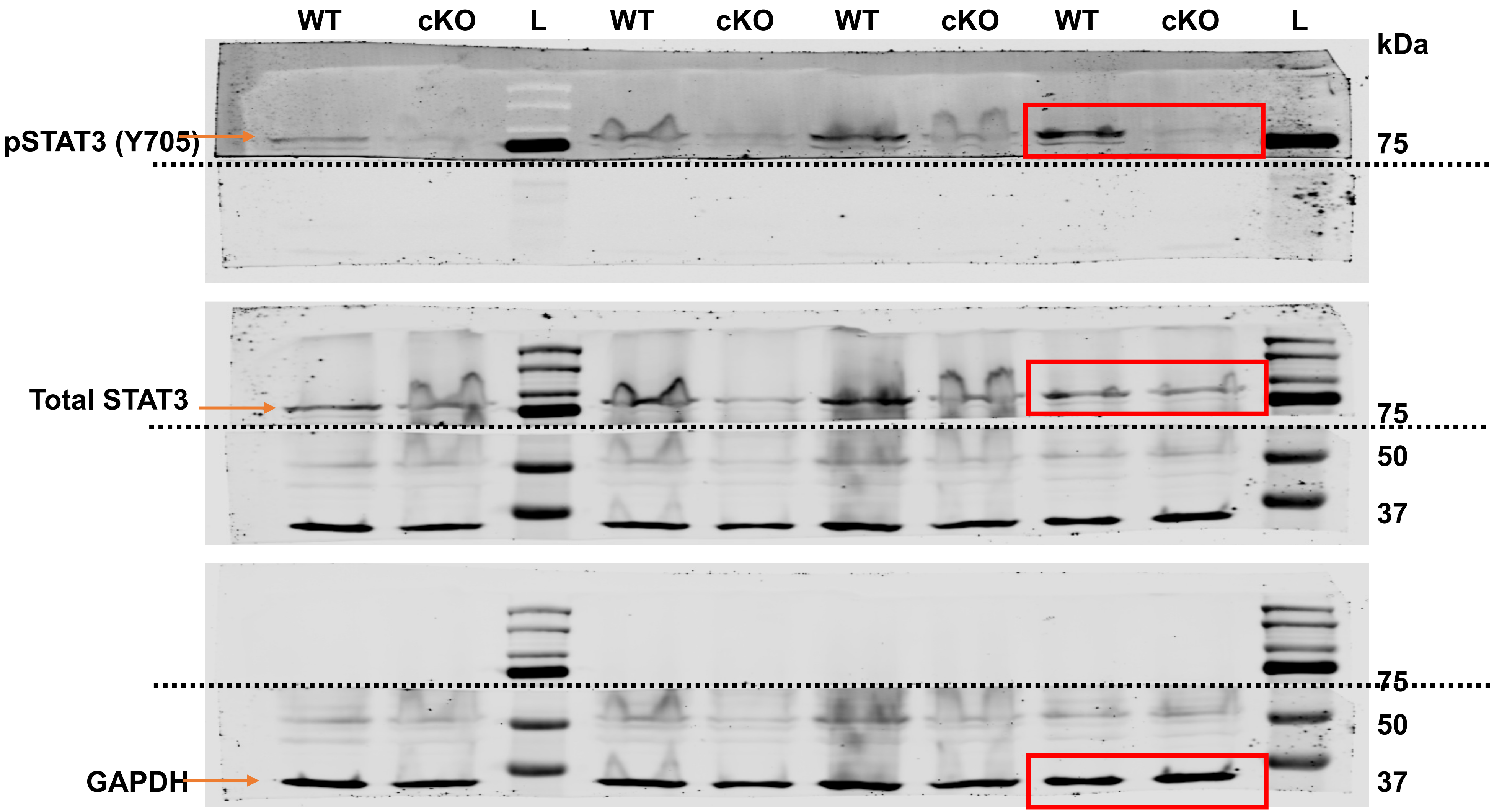

Full unedited gel for Figure S16

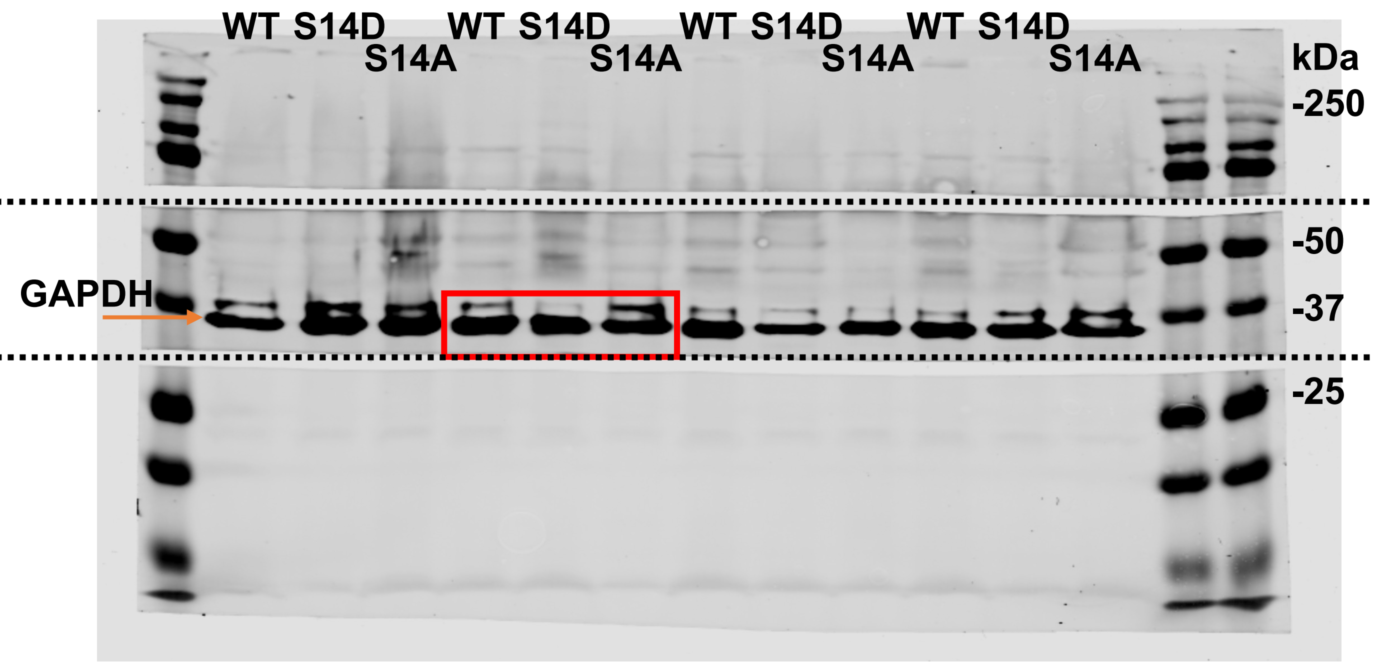

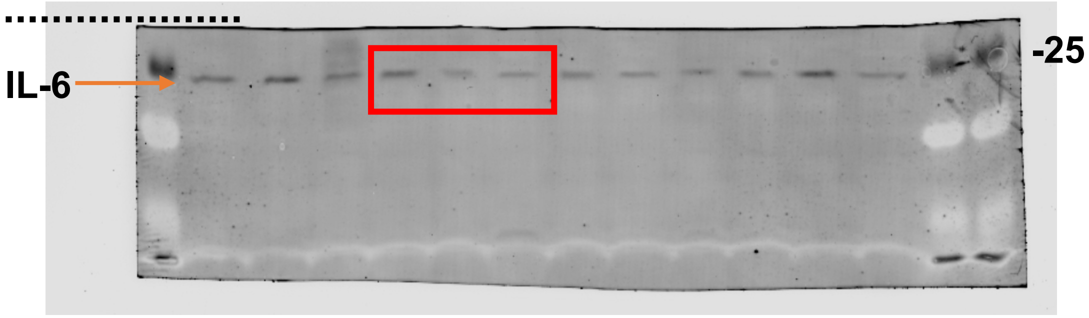

Supplement: Unedited blot and gel images [file jci-135-187711-s154.pdf]
